# Rewriting History in Integrable Stochastic Particle Systems

**DOI:** 10.1007/s00220-024-05189-y

**Published:** 2024-11-29

**Authors:** Leonid Petrov, Axel Saenz

**Affiliations:** 1 University of Virginia, Charlottesville, VA 22904 USA; 2 Oregon State University, Corvallis, OR 97331 USA

## Abstract

Many integrable stochastic particle systems in one space dimension (such as TASEP—Totally Asymmetric Simple Exclusion Process—and its *q*-deformation, the *q*-TASEP) remain integrable if we equip each particle with its own speed parameter. In this work, we present intertwining relations between Markov transition operators of particle systems which differ by a permutation of the speed parameters. These relations generalize our previous works (Petrov and Saenz in Probab Theory Relat Fields 182:481–530, 2022), (Petrov in SIGMA 17(021):34, 2021), but here we employ a novel approach based on the Yang-Baxter equation for the higher spin stochastic six vertex model. Our intertwiners are Markov transition operators, which leads to interesting probabilistic consequences. First, we obtain a new Lax-type differential equation for the Markov transition semigroups of homogeneous, continuous-time versions of our particle systems. Our Lax equation encodes the time evolution of multipoint observables of the *q*-TASEP and TASEP in a unified way, which may be of interest for the asymptotic analysis of multipoint observables of these systems. Second, we show that our intertwining relations lead to couplings between probability measures on trajectories of particle systems which differ by a permutation of the speed parameters. The conditional distribution for such a coupling is realized as a “rewriting history” random walk which randomly resamples the trajectory of a particle in a chamber determined by the trajectories of the neighboring particles. As a byproduct, we construct a new coupling for standard Poisson processes on the positive real half-line with different rates.

## Introduction

### Overview

Integrable (also called “exactly solvable”) stochastic interacting particle systems on the line are Markov chains on configurations of particles on $\mathbb {Z}$, which feature exact formulas governing their distributions at any given time. These formulas lead to precise control of the asymptotic behavior of these Markov chains in the limit to large scale and long times. In the past two decades, integrable particle systems have been instrumental in uncovering new universal asymptotic phenomena, including those present in the Kardar-Parisi-Zhang universality class. See Corwin [Cor12, Cor], Halpin-Healy–Takeuchi [HT15], and Quastel–Spohn [QS15].

Initial progress for integrable stochastic particle systems was achieved through the use of determinantal techniques, e.g., see Johansson [Joh00] for the asymptotic fluctuations of TASEP (Totally Asymmetric Simple Exclusion Process). More recently, new tools arising from quantum integrability, Bethe ansatz, and symmetric functions were applied to deformations of TASEP and related models. These deformations include ASEP, where particles may jump in both directions with asymmetric rates (Tracy–Widom [TW08, TW09]); random polymers (O’Connell [O’C12], Corwin–O’Connell–Seppäläinen–Zygouras [COSZ14], O’Connell–Seppäläinen–Zygouras [OSZ14], Seppäläinen [Sep12], Barraquand–Corwin [BC16]); and various *q*-deformations of the TASEP which modify its jump rates. Among the latter, in this paper, we consider the *q*-TASEP introduced by Borodin–Corwin [BC14] (see also Sasamoto–Wadati [SW98]), and the *q*-Hahn TASEP introduced and studied in Povolotsky [Pov13] and Corwin [Cor14].

One of the most recent achievements in the study of the structure of integrable stochastic particle systems is their unification under the umbrella of *integrable stochastic vertex models* initiated in Borodin–Corwin–Gorin [BCG16], Corwin–Petrov [CP16a], and Borodin–Petrov [BP18a, BP16a]. The integrability of the stochastic vertex models is powered by the *Yang-Baxter equation*, which is a local symmetry of the models arising from the underlying algebraic structure. This is the central starting point for studying the stochastic vertex models.

Ever since the original works on TASEP, it was clear that integrability in particle systems like TASEP is preserved when we introduce countably many extra parameters; see Gravner–Tracy–Widom [GTW02] and Its–Tracy–Widom [ITW01]. A typical example is when each particle has its own jump rate. One can trace the ability to perform such a multiparameter deformation to the underlying algebraic structure of the model, which connects it to a particular family of symmetric polynomials (e.g., the probability distribution of the TASEP may be written in terms of the Schur polynomials). In the framework of symmetric functions, interacting particle systems with different particle speeds already appeared in Vershik–Kerov [VK86] in connection with the Robinson–Schensted–Knuth (RSK) correspondence; see also O’Connell [O’C03a, O’C03b] for further probabilistic properties of the RSK.

The multiparameter deformation of integrable stochastic particle systems should be contrasted with *q*-deformations, like the one turning TASEP into the *q*-TASEP. The latter introduces just one extra parameter while at the same time deforming the underlying symmetric functions in a nontrivial way (for *q*-TASEP, passing from the Schur functions to *q*-Whittaker functions). On the other hand, our multiparameter deformations rely on the presence of symmetry itself and can be readily combined with *q*-deformations.

Let us remark that TASEP in inhomogeneous space (when the jump rate of a particle depends on its location) does not seem to be integrable; see Costin–Lebowitz–Speer–Troiani [CLST13], Janowsky–Lebowitz [JL92], and Seppäläinen [Sep01]. For this reason, control of the asymptotic fluctuations in this process requires very delicate asymptotic analysis; see Basu–Sidoravicius–Sly, [BSS14], Basu–Sarkar–Sly [BSS17]. Moreover, it is not known whether ASEP has any integrable multiparameter deformations. The stochastic six vertex model introduced and studied by Gwa–Spohn [GS92] and Borodin–Corwin–Gorin [BCG16] scales to ASEP and admits such a multiparameter deformation, see Borodin–Petrov [BP18a]. However, the scaling to ASEP destroys this structure. Recently other families of spatially inhomogeneous integrable stochastic particle systems in one and two space dimensions were studied by Assiotis [Ass20], Borodin–Petrov [BP18b], Knizel–Petrov–Saenz [KPS19], and Petrov [Pet20].

Due to the underlying algebraic structure powered by symmetric functions, certain joint distributions in integrable stochastic particle systems are *symmetric* under (suitably restricted classes of) permutations of their speed parameters. This symmetry is far from being evident from the definition of a particle system and is often observed only as a consequence of explicit formulas. In our previous works (Petrov–Saenz [PS22], Petrov [Pet21], Petrov–Tikhonov [PT20]), we explored various probabilistic consequences of these distributional symmetries. In particular, we constructed natural monotone couplings between fixed-time distributions in particle systems which differ by a permutation of the speed parameters.

However, the analysis in our previous works was severely restricted to the case of the distinguished *step* initial configuration in the particle systems and only to couplings of fixed-time distributions. This paper presents a new approach based on the Yang-Baxter equation and widely extends the scope of distributional symmetries and monotone couplings in integrable stochastic particle systems. In particular, we extend the previous results to both the general initial conditions and to couplings of measures on whole trajectories (and not only fixed-time distributions).

In the rest of the Introduction, we formulate the paper’s main results. We use a simplified notation for some Markov operators that slightly differs from the notation later used in the rest of the text. We begin by presenting concrete new probabilistic results in the well-known setting of the two-particle continuous time TASEP and the Poisson processes on $(0,+\infty )$ in Sects. 1.2 and 1.3.

### Coupling in the two-particle TASEP with different particle speeds

Before presenting our general results in Sect. 1.6 below, let us illustrate them in the simplest nontrivial case, the continuous time TASEP with two particles. Think of them as two cars, one fast and one slow, driving on a one-lane road evolving in continuous time. The speeds of the cars are $\alpha _0>\alpha _1>0$. These are the rates of independent exponential clocks associated with the cars. When a clock rings, the car jumps by 1 to the right if the destination is not occupied by a car in front of it. In particular, the car in front performs a simple continuous time Poisson random walk, while the motion of the second car is more complicated than that of the first one due to blocking from the first car.

We consider two systems, the fast-slow (FS) and the slow-fast (SF), depending on which car is in front.

#### Proposition 1.1

If the cars start next to each other in both the FS and SF systems, then the probability law of the whole trajectory $\{ x_2(t) \}_{ t\in \mathbb {R}_{\ge 0} }$ of the car in the back is the same in both systems.

**Fig. 1:**
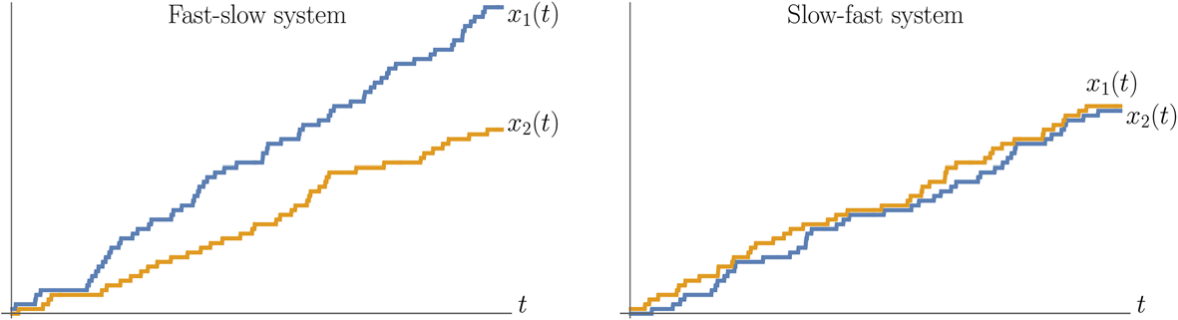
FS (left) and SF (right) systems of two cars started from the step initial configuration. In the FS system, the first car quickly runs to the front, and the evolution of the second (slow) car becomes an independent Poisson walk. In the SF system, the slow car in front often blocks the fast car in the back. However, in both systems, the trajectory of the car in the back, $\{x_2(t)\}_{t\in \mathbb {R}_{\ge 0}}$, is the same in distribution

In other words, the trajectory of the car in the back, $x_2(t)$, depends on the parameters $\alpha _0,\alpha _1$ in a symmetric way. See Fig. 1 for an illustration.

#### Idea of proof of Proposition 1.1

This statement can be traced back to the RSK construction of TASEP (presented, e.g., in Vershik–Kerov [VK86] or O’Connell [O’C03a, O’C03b]). The RSK shows that $(x_1(t),x_2(t))$ is a deterministic function of the system of two independent continuous time Poisson random walks with rates $\alpha _0$ and $\alpha _1$. While these deterministic functions are different in the SF and the FS systems, one can readily verify (for example, via the Bender–Knuth involution [BK72]) that they produce the same distribution of the trajectory $\{x_2(t)\}_{t\in \mathbb {R}_{\ge 0}}$. $\square$

**Fig. 2:**
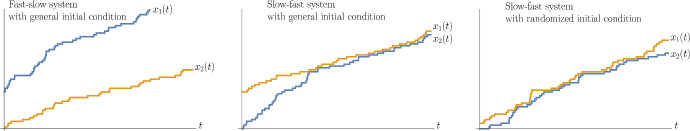
Left: The FS system started from a general fixed initial condition $(x_1^\circ ,x_2^\circ )$. Center: The SF system started from the same initial condition $(x_1^\circ ,x_2^\circ )$. Right: The SF system started from a randomized initial condition $(y_1^\circ ,x_2^\circ )$ depending on $(x_1^\circ ,x_2^\circ )$. The trajectories of the car in the back, $\{x_2(t)\}_{t\in \mathbb {R}_{\ge 0}}$, are the same in distribution in the left and the right systems but are different from the trajectory in the center system

The assumption that the cars start next to each other is crucial for Proposition 1.1. Indeed, consider the initial condition $x_1^\circ >x_2^\circ$ for the FS and SF systems so that $x_1^\circ -x_2^\circ$ is large. In the SF system, the trajectory of the car in the back first evolves as a random walk with the faster slope $\alpha _0$ and, then, has slope $\alpha _1$ after catching up with the slow car. This is very different from the behavior of the car in the back in the FS system, where the slope is $\alpha _1$ the whole time. So, Proposition 1.1 fails for an arbitrary initial condition $(x_1^\circ ,x_2^\circ )$. See Fig. 2, left and center, for an illustration.

In this paper, we suitably modify Proposition 1.1 to generalize it to arbitrary initial conditions $(x_1^\circ ,x_2^\circ )$. The modification involves a randomization of the initial condition in the SF system. Define

$$\begin{aligned} y_1^\circ :=x_2^\circ +1+\min (G, x_1^\circ -x_2^\circ -1), \end{aligned}$$

where *G* is an independent geometric random variable with parameter $\alpha _1/\alpha _0$, that is,

1.1 $$\begin{aligned} {\mathbb {P}}(G=k)=\left( 1-\alpha _1/\alpha _0 \right) (\alpha _1/\alpha _0)^{k}, \qquad k\in \mathbb {Z}_{\ge 0}. \end{aligned}$$

The Markov map which turns $(x_1^\circ ,x_2^\circ )$ into $(y_1^\circ ,x_2^\circ )$ is an instance of the Markov swap operator $P^{(n)}$ (with $n=1$ here) entering Proposition 1.6 below. For the next statement, the gap $x_1^\circ -x_2^\circ$ can be arbitrary, not necessarily large. Denote the FS and SF systems with the corresponding initial conditions by $\textrm{FS}_{x_1^\circ ,x_2^\circ }$ and $\textrm{SF}_{y_1^\circ ,x_2^\circ }$.

#### Theorem 1.2

The trajectory of the car in the back, $\{x_2(t)\}_{t\in \mathbb {R}_{\ge 0}}$, is the same in distribution for $\textrm{FS}_{x_1^\circ ,x_2^\circ }$ and $\textrm{SF}_{y_1^\circ ,x_2^\circ }$, where $y_1^\circ$ (the initial condition for the car in the front in SF) is random and given by (1.1).

See Fig. 2 for an illustration. When $x_1^\circ =x_2^\circ +1$, from (1.1) we almost surely have $y_1^\circ =x_1^\circ$, and so Theorem 1.2 reduces to Proposition 1.1. For general initial conditions, the intertwining result, i.e. Proposition 1.6 introduced in Sect. 1.5 below, is not enough to conclude the equality in distribution of the whole trajectories. Namely, Proposition 1.6 only implies the equality in distribution of $x_2(t)$ in $\textrm{FS}_{x_1^\circ ,x_2^\circ }$ and $\textrm{SF}_{y_1^\circ ,x_2^\circ }$ at each fixed time *t*, but not *jointly* for all times. We need a stronger coupling between measures briefly described in Sect. 1.6 below. To point to the relevant results in the main text, Theorem 1.2 follows from the general Theorem 7.9 and its continuous-time corollary, Proposition 9.2 (in particular, see Remark 9.3 for the TASEP case).

Let us describe the coupling between the two-particle systems $\textrm{FS}_{x_1^\circ ,x_2^\circ }$ and $\textrm{SF}_{y_1^\circ ,x_2^\circ }$ which leads to Theorem 1.2. Fix a terminal time $M\in \mathbb {R}_{\ge 0}$. The coupling (*rewriting history operator from future to past*, in our terminology) replaces the trajectory $\{x_1(t)\}_{0\le t\le M}$ in $\textrm{FS}_{x_1^\circ ,x_2^\circ }$ by a new trajectory $\{y_1(t)\}_{0\le t\le M}$ such that $x_2(t)<y_1(t)\le x_1(t)$ for all *t* (so that the coupling is *monotone*). The construction of $y_1(t)$ proceeds in two steps:

- First, at time $t=M$, set $y_1(M)=x_2(M)+1+\min (G,x_1(M)-x_2(M)-1)$, where *G* is an independent geometric random variable with parameter $\alpha _1/\alpha _0$, as in (1.1).
- Then, start a continuous time Poisson random walk $y_1(t)$ in reverse time from *M* to 0 in the chamber $x_2(t)<y_1(t)\le x_1(t)$, under which the car $y_1$ jumps down by 1 at rate $\alpha _0$ if $y_1(t)<x_1(t)$ and at rate $\alpha _0-\alpha _1$ if $y_1(t)=x_1(t)$. If $y_1(t)=x_2(t)+1$, the jump down is blocked. Also, if the top boundary of the chamber goes down by 1, the walk $y_1(t)$ is deterministically pushed down.

**Fig. 3:**
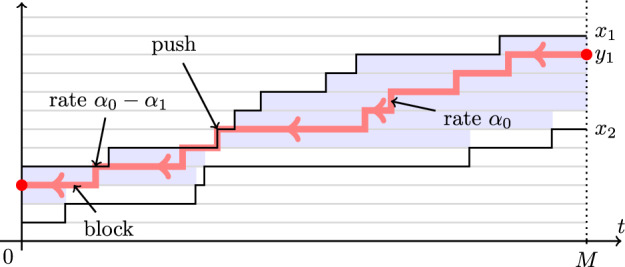
Construction of the coupling from FS to SF systems. The random walk $y_1(t)$ in reverse time is confined to the highlighted chamber. In the bulk of the chamber, its rate of jumping down is $\alpha _0$, and on the top boundary the rate is $\alpha _0-\alpha _1$. On the bottom boundary, the jumps down are blocked

See Fig. 3 for an illustration. Proposition 9.2 implies that the distribution of the two-particle trajectory $\{y_1(t),x_2(t)\}_{0\le t\le M}$ is the same as the trajectory of the whole two-particle system $\textrm{SF}_{y_1^\circ ,x_2^\circ }$. In particular, the position $y_1(0)$ has the distribution $y_1^\circ$ given by (1.1), and Theorem 1.2 follows.

The resampling of a trajectory as a random walk in a chamber is reminiscent of the Brownian Gibbs property from Corwin–Hammond [CH14]. However, there are several notable differences. First, our resampling does not preserve the distribution but interchanges the rates $\alpha _0 \leftrightarrow \alpha _1$ (though in a continuous time limit as in Sect. 1.5, this issue disappears). Second, the resampled trajectory is not a simple random walk *conditioned* to stay in the chamber like in the Brownian Gibbs property, but rather a random walk *reflected* from the bottom wall and *sticking* to the top wall. In the Brownian setting, processes sticking to one of the walls appeared in Warren [War97] and Howitt–Warren [HW09] in a setting similar to ours, see Section 1.3 in Petrov–Tikhonov [PT20] for a related discussion. Finally, our resampling changes the trajectory and its endpoints, while under the Brownian Gibbs property, the endpoints stay fixed. We plan to explore Brownian limits of our constructions in future work.

#### Remark 1.3

The Yang-Baxter equation proof of Theorem 1.2 implies a more general fact. Namely, one can *condition* on a fixed non-random movement of the first car, and the distributions of the car in the back in two systems are still the same. Conditioning on a fixed motion of the first car appeared (under the name of the “moving wall”) in Borodin–Bufetov–Ferrari [BBF24].

### Coupling of Poisson processes

In the previous Sect. 1.6, we described a monotone coupling from FS to SF, which turns the trajectory $x_1(t)$, $0\le t\le M$, in FS having speed $\alpha _0$ into a trajectory $y_1(t)$ which lies below $x_1(t)$ and has lower speed $\alpha _1<\alpha _0$. The law of $y_1(t)$ depends on the trajectory $x_2(t)$ of the particle in the back.

Along with this monotone coupling, there is a monotone coupling in another direction, *rewriting history operator from past to future*, which turns the particle’s trajectory in front of speed $\alpha _1$ into a trajectory with a higher speed $\alpha _0>\alpha _1$. Recall that in TASEP, the trajectories of the particles in front are continuous time Poisson simple random walks, that is, they are *counting functions of the standard Poisson point processes* on $(0,+\infty )$. Remarkably, when FS and SF start from the step initial configuration $x_1(0)=y_1(0)=0$, $x_2(0)=-1$, the operator for rewriting history from past to future does not depend on the trajectory $x_2(t)$. Thus, it produces a monotone coupling between two Poisson simple random walks of rates $\alpha _1$ and $\alpha _0>\alpha _1$, respectively. Let us describe this coupling.

**Fig. 4:**
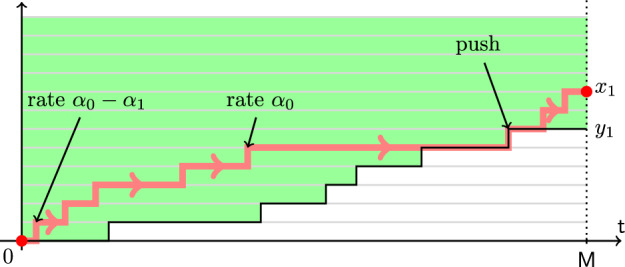
Monotone coupling from a Poisson random walk $y_1(t)$ of rate $\alpha _1$ to a Poisson random walk $x_1(t)$ of rate $\alpha _0>\alpha _1$. The new process must lie in the highlighted chamber. In the bulk of the chamber, it has jump rate $\alpha _0$, and on the bottom boundary the jump rate is $\alpha _0-\alpha _1$

Let $y_1(t)$, $t\in \mathbb {R}_{\ge 0}$, be a Poisson simple random walk of rate $\alpha _1$ started from $y_1(0)=0$. Fix $\alpha _0>\alpha _1$. Start another random walk $x_1(t)$ from $x_1(0)=0$ which lives in the chamber $x_1(t)\ge y_1(t)$ for all *t*, and jumps up by 1 at rate $\alpha _0$ if $x_1(t)>y_1(t)$, and at rate $\alpha _0-\alpha _1$ if $x_1(t)=y_1(t)$. When the bottom boundary of the chamber goes up by 1 and $x_1(t)=y_1(t)$, the walk $x_1(t)$ is deterministically pushed up. See Fig. 4 for an illustration.

#### Theorem 1.4

The process $x_1(t)$ defined above (right before the Theorem) is a continuous time Poisson simple random walk with rate $\alpha _0$.

Theorem 1.4 follows from our main result on coupling, Theorem 7.9, and its corollary for rewriting history from past to future in continuous time, Proposition 9.4 (in particular, see Remark 9.5 for the TASEP case).

#### Remark 1.5

We note that there is a well-known and simple randomized coupling between two Poisson processes on $(0,+\infty )$ (from a rate $\alpha _1$ to the higher rate $\alpha _0>\alpha _1$). It is called *thickening* and consists of simply adding to the process of rate $\alpha _1$ an extra independent Poisson point process configuration of rate $\alpha _0-\alpha _1$. The union of the two point configurations is a Poisson process of rate $\alpha _0$. The construction in Theorem 1.4 is very different from such an independent thickening: it does not preserve all the points from the original process of rate $\alpha _1$, and has a Markov (and not independent) nature.

Surprisingly, there also exist *deterministic* couplings between Poisson processes on the entire line $\mathbb {R}$ from higher to lower rates which are translation invariant (constructed by Ball [Bal05]), and also non-translation invariant ones on an arbitrary set in both $\alpha _0$ to $\alpha _1$ and the reverse directions, see Angel–Holroyd–Soo [AHS11] and Gurel-Gurevich–Peled [GGP13].

It is possible to make the Poisson rates $\alpha _0$ and $\alpha _1$ equal to each other by taking a continuous time Poisson type limit. This limit produces an interesting (and, to the best of our knowledge, new) continuous time coupling between Poisson processes on $(0,+\infty )$ of all possible rates. This coupling is also monotone; that is, it increases the rate while almost surely increasing the trajectory of the Poisson process’ counting function. This continuous time coupling is described in Proposition 10.13.

### Intertwining relations for stochastic vertex models

After highlighting two concrete applications in Sects. 1.2 and 1.3, let us present our results in a more general setting.

In the setting of the fully fused higher spin stochastic six vertex model, we prove an *intertwining* (also called *quasi-commutation*) relation between the transfer matrices of two models, which differ by a permutation of the speed parameters. This vertex model is defined in Corwin–Petrov [Cor14] and Borodin–Petrov [BP18a]; we recall it in Sects. 2 and 3 below. We formulate the intertwining as follows. Let us denote by *T* and $T_{\sigma _{n-1}}$ the one-step Markov operators (transfer matrices) on the space $\left\{ 0,1 \right\} ^{\mathbb {Z}}$ of particle configurations on $\mathbb {Z}$. The parameter sequences in *T* and $T_{\sigma _{n-1}}$ differ by the elementary transposition $\sigma _{n-1}=(n-1,n)$ which permutes the parameters associated with two neighboring particles $x_n > x_{n+1}$.^1^

#### Proposition 1.6

(Proposition 4.2 in the text). There exists a one-step Markov transition operator denoted by $P^{(n)}$ such that

1.2 $$\begin{aligned} T\hspace{1pt}P^{(n)}=P^{(n)}\hspace{1pt}T_{\sigma _{n-1}}, \end{aligned}$$

under a certain restriction on the parameters associated with the particles $x_n$ and $x_{n+1}$.

The action of the Markov operator $P^{(n)}$ only moves the particle $x_n$ while preserving the locations of all other particles. Here and throughout the paper, we interpret the product of Markov operators as acting on measures from the right. That is, (1.2) states that if we start from a fixed particle configuration, apply a random Markov step according to *T*, and then apply a Markov step according to $P^{(n)}$, then the resulting random particle configuration has the same distribution as the random particle configuration obtained by the action of $P^{(n)}$ followed by $T_{\sigma _{n-1}}$.

The intertwining relation (1.2) is a consequence of the Yang-Baxter equation for the higher spin stochastic six vertex model. Our crucial observation is that under certain restrictions on the parameters, the intertwiner $P^{(n)}$ (coming from the corresponding R-matrix for the vertex model) is itself a one-step Markov transition operator. The restrictions on the parameters are required to make the transition probabilities of $P^{(n)}$ nonnegative.

Furthermore, we show that a sequential application of the operators $P^{(n)}$ over all $n=1,2,3,\ldots$ (denoted by *B*) intertwines the transfer matrix *T* with another transfer matrix $T_{\textrm{shift}}$ obtained from *T* by the one-sided shift of the parameter sequence (which eliminates the first of the parameters with index 0):

1.3 $$\begin{aligned} TB=B\hspace{1pt}T_{\textrm{shift}}. \end{aligned}$$

See Theorem 4.7 in the text. We call *B* the *Markov shift operator*. See Sect. 4 for detailed formulations and proofs of the general intertwining relations (1.2), (1.3).

### Intertwining and Lax equation for the continuous time *q*-TASEP

In Sect. 1.4, the transfer matrices $T,T_{\textrm{shift}}$ and the intertwiner *B* are one-step Markov transition operators. It is well-known that the vertex model transfer matrices *T* admit a Poisson type limit to the *q*-TASEP.

Recall that the *q*-TASEP, introduced in Borodin–Corwin [BC14], is a continuous time Markov chain on particle configurations $\textbf{x}=(x_1>x_2>x_3>\ldots )$ in $\mathbb {Z}$. Each particle $x_n$ has an independent exponential clock of rate^2^$\alpha _{n-1}(1-q^{x_n-x_{n+1}-1})$. When the clock attached to $x_n$ rings, this particle jumps by 1 to the right. Note that the jump rate of $x_n$ is zero when $x_n=x_{n+1}+1$, meaning that a particle cannot jump into an occupied location. When $q=0$, the *q*-TASEP turns into the usual TASEP in which each particle jumps to the right at rate 1 unless the destination is occupied.

Now, take the particle speeds in the *q*-TASEP to be the geometric progression, $\alpha _j=r^j$, $j\in \mathbb {Z}_{\ge 0}$, where $0<r<1$. Sending $r\rightarrow 1$ leads to the *q*-TASEP with homogeneous speeds. Let $\{T(t)\}_{t\in \mathbb {R}_{\ge 0}}$ denote the corresponding Markov transition semigroup. Before taking the limit $r \rightarrow 1$, the application of the Markov shift operator *B* as in (1.3) turns the sequence of speeds $(1,r,r^2,\ldots )$ into $(r,r^2,r^3,\ldots )$. This shift is the same as multiplying all particle speeds by *r* or, equivalently, turning the time parameter *t* into *rt*. Taking a second Poisson-type limit in *B* as $r\rightarrow 1$, we obtain an intertwining relation for the continuous time *q*-TASEP with homogeneous speeds.

Let us now describe the continuous time limit of the Markov shift operators. This is a Markov semigroup $\{B(\tau )\}_{\tau \in \mathbb {R}_{\ge 0}}$ on the space of *left-packed* particle configurations $\textbf{x}=(x_1>x_2>\ldots )$, i.e., configurations with $x_n=-n$ for all sufficiently large *n*. Under $B(\tau )$, each particle $x_n$ has an independent exponential clock with rate $n(x_n-x_{n+1}-1)$. When a clock rings, the corresponding particle $x_n$ instantaneously jumps backwards to a new location $x_n'$, $x_{n+1}< x_n'<x_n$, with probability

1.4 $$\begin{aligned} \frac{1}{(x_n-x_{n+1}-1)(1-q^{x_n-x_n'})} \frac{\prod _{i=1}^{x_n-x_{n+1}-1}(1-q^i)}{\prod _{i=1}^{x_n'-x_{n+1}-1}(1-q^i)}. \end{aligned}$$

**Fig. 5:**
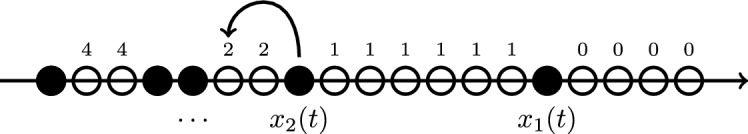
The $q=0$ version of the backwards process $B(\tau )$ may be alternatively defined as follows. Each hole in $\mathbb {Z}$ has an independent exponential clock with a rate equal to the number of particles to the right of this hole. When the clock at a hole rings, the leftmost of the particles to the right of the hole instantaneously jumps into this hole

In particular, the particles almost surely jump to the left. For left-packed configurations, the sum of the jump rates of all possible particle jumps is finite, meaning that $B(\tau )$ is well-defined. The process $B(\tau )$ was introduced in Petrov [Pet21], and its $q=0$ version (for which the probabilities (1.4) become uniform) appeared under the name *backwards Hammersley process* in Petrov–Saenz [PS22]. See Fig. 5 for an illustration of the latter process.

We prove the following intertwining relation between the *q*-TASEP and the backwards *q*-TASEP processes:

#### Theorem 1.7

(Theorem 5.6 in the text). For any $t,\tau \in \mathbb {R}_{\ge 0}$, we have

1.5 $$\begin{aligned} T(t)\hspace{1pt}B(\tau ) = B(\tau )\hspace{1pt}T(e^{-\tau }t). \end{aligned}$$

Let us reformulate the intertwining relation (1.5) in probabilistic terms. Fix a left-packed configuration $\textbf{y}$, and let $\delta _{\textbf{y}} B(\tau )$ be a *random* configuration obtained from $\textbf{y}$ by running the backwards *q*-TASEP dynamics for time $\tau$. Then, denote by $\textbf{x}(t)$ the configuration of the *q*-TASEP at time *t* started with initial condition $\textbf{x}(0)=\textbf{y}$. Now, fix $\tau$, and run the backwards *q*-TASEP dynamics from the configuration $\textbf{x}(\textsf{t})$ for time $\tau$. Then, the distribution of the resulting configuration is *the same* as the distribution of the *q*-TASEP at time $e^{-\tau }\textsf{t}$ but started from the *random* initial configuration $\delta _{\textbf{y}} B(\tau )$.

Identity (1.5) reduces to the result obtained earlier in Petrov–Saenz [PS22] and Petrov [Pet21] when applied to the process started from the distinguished *step* initial configuration $\textbf{y}=\textbf{x}_{step}$ for which $x_n(0)=-n$ for all $n\in \mathbb {Z}_{\ge 1}$. That is,

1.6 $$\begin{aligned} \delta _{\textbf{x}_{step}} \hspace{1pt}T(t)\hspace{1pt}B(\tau ) = \delta _{\textbf{x}_{step}} \hspace{1pt}T(e^{-\tau }t). \end{aligned}$$

Indeed, the action of $B(\tau )$ preserves the step configuration since it moves all the particles left. In probabilistic terms, (1.6) shows that the backwards process $B(\tau )$ produces a coupling in the reverse time direction of the fixed-time distributions of the *q*-TASEP with the step initial configuration.

We arrive at the following *Lax equation* for the *q*-TASEP semigroup:

1.7 $$\begin{aligned} t\hspace{1pt}\frac{d}{dt}\hspace{1pt}T(t)=\left[ \textsf{B},T(t) \right] , \end{aligned}$$

where $\textsf{B}$ is the infinitesimal Markov generator of the backwards *q*-TASEP. This follows by differentiating (1.5) in $\tau$ at $\tau =0$ and slightly rewriting the result using the Kolmogorov (a.k.a the Fokker–Planck) equation. Equivalently, in terms of expectations, for any left-packed initial configuration $\textbf{y}$ and a generic function *F* of the configuration, we have the following evolution equation for the observables:

1.8 $$\begin{aligned} t\hspace{1pt}\frac{d}{dt} \hspace{1pt}\mathbb {E}_{\textbf{y}}\left( F(\textbf{x}(t)) \right) = \textsf{B} \hspace{1pt}\mathbb {E}_{\textbf{y}} \left[ F(\textbf{x}(t)) \right] - \mathbb {E}_{\textbf{y}}\left[ (\textsf{B}\hspace{1pt}F)(\textbf{x}(t)) \right] . \end{aligned}$$

We use the notation $\mathbb {E}_{\textbf{y}}$ to denote the expectation with respect to the *q*-TASEP started from $\textbf{y}$. The first generator $\textsf{B}$ in the right-hand side of (1.8) acts on $\mathbb {E}_{\textbf{y}} \left[ F(\textbf{x}(t)) \right]$ as a function of $\textbf{y}$, while the second one acts on the function *F*.

It is intriguing that while $\textsf{B}$ and *T*(*t*) depend on *q*, the form of the Lax equations (1.7)–(1.8) is *the same* for the *q*-TASEP and its $q=0$ specialization, the TASEP. Though the structure and asymptotics of multipoint observables of TASEP is well-studied by now (e.g., see Liu [Liu22], and Johansson–Rahman [JR21]), its extension to *q*-TASEP is mostly conjectural at this point, see Dotsenko [Dot13], Prolhac–Spohn [PS11], Imamura–Sasamoto–Spohn [ISS13], and Dimitrov [Dim20] for related results.

We believe that our Lax equation could be employed to study multipoint asymptotics of the *q*-TASEP and, in a scaling limit, lead to Kadomtsev-Petviashvili (KP) or Korteweg-de Vries (KdV) type equations for limits of the observables (1.8). The KP and KdV equations were recently derived by Quastel–Remenik [QR22] for the KPZ fixed point process introduced earlier by Matetski–Quastel–Remekin [MQR21]. We leave the asymptotic analysis of the Lax equation to future work.

### Coupling of measures on trajectories

Let us briefly outline the scope of couplings between trajectories of various integrable stochastic particle systems obtained in the present paper. All these couplings, including the examples from Sects. 1.2 and 1.3, are obtained from the intertwining relations like (1.2), (1.5) through the *bijectivisation* procedure. This idea originated in Diaconis–Fill [DF90] and was later developed in the context of integrable stochastic particle systems in Borodin–Ferrari [BF14], Borodin–Gorin [BG09], Borodin–Petrov [BP16b], and Bufetov–Petrov [BP19]. The building block of all the intertwining relations is the Yang-Baxter equation. We first apply the bijectivisation procedure to the Yang-Baxter equation and obtain elementary Markov steps. They are conditional distributions corresponding to a coupling between two marginal distributions coming from two sides of the Yang-Baxter equation. The bijectivisation of the Yang-Baxter equation is not unique because the coupling is not unique. We focus on the simplest case, which has the “maximal noise”. This is the case that introduces the most randomness and independence. We recall these constructions in Sect. 7. Let us now describe the couplings we obtain.

First, we begin with the intertwining relation (1.2) for the fully general fused stochastic higher spin six vertex model. Graphically it is represented as follows: 
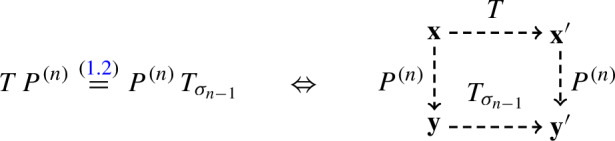
 In words, fix a particle configuration $\textbf{x}$, apply the Markov operator *T*, and then $P^{(n)}$. Relation (1.2) implies that the distribution of the resulting random configuration $\textbf{y}'$ is the same as if we first applied $P^{(n)}$ and then $T_{\sigma _{n-1}}$. Section 7.3 defines two couplings based on the intertwining (1.2). The coupling $D^{(n)}$ from the left- to the right-hand side of (1.2) (*future to past* in our terminology) samples $\textbf{y}$ given $\textbf{x},\textbf{x}',\textbf{y}'$. The coupling $U^{(n)}$ in the opposite direction (*past to future* in our terminology) samples $\mathbf {x'}$ given $\textbf{x},\textbf{y},\textbf{y}'$. Both couplings are compatible with (1.2) in the sense that they satisfy a *detailed balance equation*; see (7.8) in the text.

Iterating the couplings $D^{(n)},U^{(n)}$ over time, we obtain a bijectivisation of the relation $T^k P^{(n)}=P^{(n)}\hspace{1pt}T_{\sigma _{n-1}}^k$ for any time $k\in \mathbb {Z}_{\ge 1}$. This leads to two discrete-time Markov operators for rewriting history (in two directions). Their general construction is given in Definition 7.10 after Theorem 7.9, and the general definitions are expanded in terms of particle systems in Sect. 8.

We write down concrete operators for rewriting history in *q*-TASEP in Sects. 9.3 and 9.4 by keeping *n* fixed, taking the Poisson type limit as $k\rightarrow \infty$ to continuous time, and specializing to *q*-TASEP. For $q=0$ and $n=1$, these rewriting history operators give rise to the coupling results for the two-particle TASEP and Poisson processes presented in Sects. 1.2 and 1.3 above.

Furthermore, we get a bijectivisation of the intertwining relation $T^kB=B\hspace{1pt}T_{\textrm{shift}}^k$ for any time $k\in \mathbb {Z}_{\ge 1}$. This relation is an iteration of (1.3) over *k*. Namely, a bijectivisation of $T^kB=B\hspace{1pt}T_{\textrm{shift}}^k$ is obtained by iterating $D^{(n)}, U^{(n)}$ over $n=1,2,\ldots$ and then iterating over time *k*, respectively.

Next, we specialize to *q*-TASEP, take the Poisson type limit as $k\rightarrow \infty$ to continuous time, and further take another Poisson type limit in the particle speeds $\alpha _j=r^j$ as $r\rightarrow 1$ as explained in Sect. 1.5. This leads to a bijectivisation of the continuous time relation $T(t)\hspace{1pt}B(\tau )=B(\tau )\hspace{1pt}T(e^{-\tau }t)$. The latter bijectivisation is a pair of continuous time Markov processes on the space of *q*-TASEP trajectories, which either speeds up or slows down the time in the process with homogeneous particle speeds. These rewriting history processes in continuous time are constructed and described in Sect. 9. See Propositions 10.7 and 10.10 for the main results.

While our exploration of couplings is extensive, in this paper we only describe some of the possible constructions. One can continue our methods in the following directions:

- Colored stochastic higher spin vertex models, introduced and studied in Borodin–Wheeler [BW22] and further works, also possess stochastic Yang-Baxter equations leading to intertwining, couplings, and corresponding Lax equations.
- Within uncolored systems (the setting of the present paper), there are two natural directions. First, taking different bijectivisations of the Yang-Baxter equation (which are not maximally independent) could produce more couplings of particle system trajectories and Poisson processes with other nontrivial properties.
- We mainly restricted our couplings to the *q*-TASEP, for which the intertwiner *B* preserves the distinguished step configuration. In a second natural direction within uncolored systems, focusing on the Schur vertex model (discussed in Sect. 6 below), we see that the intertwiner *does not* preserve the step configuration. Thus, the resulting couplings would not be monotone. It would be interesting to see which probabilistic properties these couplings still satisfy.

We plan to address these directions in future work.

### Outline

The paper consists of two parts. In the first part, we derive intertwining relations and study their consequences. In more detail, in Sections 2, we recall the stochastic higher spin six vertex models, and in Sect. 3, write down a “vertical” Yang-Baxter equation for them. We also investigate conditions under which the cross vertex weights (that is, the R-matrix) are nonnegative and thus lead to Markov transition operators. Then, in Sect. 4, we prove our main intertwining results which in full generality follow directly from the Yang-Baxter equation. In Sect. 5 and Sect. 6, we specialize the general intertwining relations to concrete particle systems such as the *q*-Hahn TASEP, the *q*-TASEP, the TASEP, and the Schur vertex model.

In the second part, we use the intertwining relations to construct couplings between probability measures on trajectories of particle systems which differ by a permutation of the speed parameters. The couplings are based on the bijectivisation procedure for the Yang-Baxter equation, which we review in Sect. 7. The conditional distribution for such a coupling is realized as a “rewriting history” process that randomly resamples a particle system’s trajectory. In Sect. 8, we construct rewriting history processes for general discrete time integrable stochastic interacting particle systems. In Section 9, we specialize our constructions to concrete rewriting history dynamics for the continuous time *q*-TASEP and TASEP. Finally, in Sect. 10, we take a limit of our couplings to the case of homogeneous particle speeds, making the rewriting history dynamics evolve in continuous time. As a byproduct, in Sect. 10.5, we construct a new coupling of the standard Poisson processes on the positive real half-line with different rates.

**Part I Intertwining Relations for Integrable Stochastic Systems**

In the first part, we obtain new intertwining relations between transfer matrices (viewed as one-step Markov transition operators) of the stochastic higher spin six vertex model with different sequences of parameters. The intertwining operators come from the R-matrix in the vertical Yang-Baxter equation, and are also Markov transition operators.

## Stochastic Higher Spin Six Vertex Model and Exclusion Process

Here we recall the most general integrable stochastic particle system considered in the paper, in both vertex model and exclusion process settings. This material is well-developed in several works on stochastic vertex models. In our exposition we follow [CP16b] and [BP18a].

### The *q*-deformed beta-binomial distribution

We need the *q-deformed beta-binomial distribution* $\varphi _{q,\mu ,\nu }$ from [Pov13, Cor14]. Let $q\in [0,1)$. Throughout the paper, we use the following notation for the *q*-Pochhammer symbols

2.1 $$\begin{aligned} &  (a;q)_k:=(1-a)(1-aq)\ldots (1-aq^{k-1}),\quad k\ge 1; \nonumber \\ &  \qquad (a;q)_0:=1, \qquad (a;q)_{\infty }:=\prod _{i=0}^{\infty }(1-aq^i). \end{aligned}$$

For $k\le -1$, we use the standard convention

2.2 $$\begin{aligned} (a;q)_k=\frac{1}{(a/q;1/q)_{-k}}. \end{aligned}$$

For $m\in \mathbb {Z}_{\ge 0}$, consider the following distribution on $\left\{ 0,1,\ldots ,m \right\}$:

2.3 $$\begin{aligned} \varphi _{q,\mu ,\nu }(j\mid m)= \mu ^j\,\frac{(\nu /\mu ;q)_j(\mu ;q)_{m-j}}{(\nu ;q)_m} \frac{(q;q)_m}{(q;q)_j(q;q)_{m-j}}, \qquad 0\le j\le m. \end{aligned}$$

Throughout the paper, we sometimes write $\varphi _{q,\mu ,\nu }(j\mid m)$ when $j>m$ or $j<0$, and agree that this expression equals zero in those cases.

When $m=+\infty$, extend the definition as

2.4 $$\begin{aligned} \varphi _{q,\mu ,\nu }(j\mid \infty )= \mu ^j\frac{(\nu /\mu ;q)_j}{(q;q)_j}\frac{(\mu ;q)_\infty }{(\nu ;q)_\infty }, \qquad j\in \mathbb {Z}_{\ge 0}. \end{aligned}$$

The quantities (2.3) and (2.4) sum to one:

$$\begin{aligned} \sum _{j=0}^{m}\varphi _{q,\mu ,\nu }(j\mid m)=1,\qquad m \in \left\{ 0,1,\ldots \right\} \cup \left\{ +\infty \right\} . \end{aligned}$$

The distribution $\varphi _{q,\mu ,\nu }$ depends on $q\in [0,1)$ and two other parameters $\mu ,\nu$. We will use the following two cases in which the weights $\varphi _{q,\mu ,\nu }(j\mid m)$ are nonnegative (and hence define a probability distribution)^3^

2.5 $$\begin{aligned}&0\le \mu \le \,\,1 \,\, {\hbox {and}}\,\, \nu \le \mu ; \end{aligned}$$

2.6 $$\begin{aligned}&\nu \le 0 \,\,{\hbox { and}}\,\, \mu =q^J\nu \,\, {\hbox {for some}}\,\, J\in \mathbb {Z}_{\ge 0}. \end{aligned}$$

### Stochastic vertex weights

We consider the *stochastic higher spin six vertex weights* $L^{(J)}_{u,s}$ which depend on the following parameters:

2.7 $$\begin{aligned} q\in [0,1), \qquad u\in [0,+\infty ), \qquad s\in (-1,0], \qquad J\in \mathbb {Z}_{\ge 1}. \end{aligned}$$

Here *q* is the main “quantum” parameter, fixed throughout the paper, and all other parameters may vary from vertex to vertex. The weights $L_{u,s}^{(J)}(i_1,j_1;i_2,j_2)$ are indexed by a quadruple of integers, where $i_1,i_2 \in \mathbb {Z}_{\ge 0}$ and $j_1,j_2\in \left\{ 0,1,\ldots ,J \right\}$, and are defined as

2.8 $$\begin{aligned} \begin{aligned} L^{(J)}_{u,s}(i_1,j_1;i_2,j_2)&:=\textbf{1}_{i_1+j_1=i_2+j_2} \frac{(-1)^{i_1}q^{ \frac{1}{2} i_1(i_1+2j_1-1)} u^{i_1}s^{j_1+j_2-i_2}(u s^{-1};q)_{j_2-i_1}}{(q;q)_{i_2} (s u;q)_{i_2+j_2} (q^{J+1-j_1};q)_{j_1-j_2}} \\ &\times _{4}\bar{\phi }_3\left( \begin{matrix} q^{-i_2};q^{-i_1},s u q^{J},q s/u\\ s^{2},q^{1+j_2-i_1},q^{J+1-i_2-j_2}\end{matrix} \bigg |\, q,q\right) . \end{aligned} \end{aligned}$$

Here and throughout the paper the notation $\textbf{1}_{A}$ means the indicator of an event or a condition *A*, and $_4\bar{\phi }_3$ is the regularized (terminating) *q*-hypergeometric series, where

2.9 $$\begin{aligned} \begin{aligned} _{r+1}\bar{\phi }_r \left( \begin{matrix} q^{-n};a_1,\ldots ,a_r \\ b_1, \ldots ,b_r \end{matrix} \bigg |\, q,z\right)&:= _{r+1}{\phi }_r \left( \begin{matrix} q^{-n},a_1,\ldots ,a_r\\ b_1,\ldots ,b_r \end{matrix} \bigg |\, q,z\right) \prod _{i=1}^{r}(b_i;q)_n \\&= \sum _{k=0}^{n} \frac{z^k (q^{-n};q)_k}{(q;q)_k} \prod _{i=1}^{r}(a_i;q)_k(b_iq^k;q)_{n-k}. \end{aligned} \end{aligned}$$

**Fig. 6:**
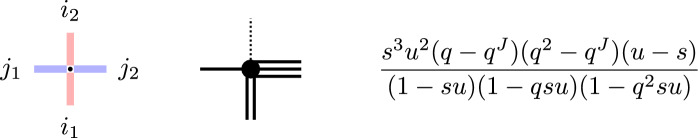
Left: Notation of incoming and outgoing path counts at a vertex. Center: The vertex of type (2, 1; 0, 3). Right: The weight $L^{(J)}_{u,s}(2,1;0,3)$

The condition that $L^{(J)}_{u,s}(i_1,j_1;i_2,j_2)$ vanishes unless $i_1+j_1=i_2+j_2$ is the *path conservation property*: the total number of incoming paths (from below and from the left) is equal to the total number of outgoing paths (to the right and upwards) at a vertex; see Fig. 6 for an illustration.

The vertex weights $L^{(J)}_{u,s}$ are called *stochastic* because they satisfy the following properties:

#### Proposition 2.1

If the parameters *q*, *u*, *s*, *J* satisfy (2.7), then

**1.**: We have $0\le L^{(J)}_{u,s}(i_1,j_1;i_2,j_2)\le 1$ for all $i_1,i_2 \in \mathbb {Z}_{\ge 0}$ and $j_1,j_2\in \left\{ 0,1,\ldots ,J \right\}$.
**2.**: For any fixed $i_1\in \mathbb {Z}_{\ge 0}$ and $j_1 \in \left\{ 0,1,\ldots ,J \right\}$, we have
  2.10 $$\begin{aligned} \sum _{i_2=0}^{\infty }\sum _{j_2=0}^{J} L^{(J)}_{u,s}(i_1,j_1;i_2,j_2) =1. \end{aligned}$$
  Note that due to the path conservation, this sum is always finite.

#### Idea of proof

First, observe that for $J=1$ the sum in (2.9) reduces to at most one term, and the vertex weights $L^{(1)}_{u,s}$ become the following explicit rational functions:

2.11 $$\begin{aligned} \begin{array}{ll} L^{(1)}_{u,s}(g,0;g,0)=\dfrac{1-q^g s u}{1-s u},& \hspace{30pt} L^{(1)}_{u,s}(g,0;g-1,1)= \dfrac{-su(1-q^g)}{1-su}; \\[10pt] L^{(1)}_{u,s}(g,1;g,1)= \dfrac{-su+q^g s^2}{1-su},& \hspace{30pt} L^{(1)}_{u,s}(g,1;g+1,0)= \dfrac{1-q^g s^2}{1-su}. \end{array} \end{aligned}$$

Then, both statements of the proposition are immediate for $J=1$. The case of arbitrary *J* follows from the $J=1$ case using the *stochastic fusion procedure*. This involves stacking *J* vertices with weights $L^{(1)}_{u,s}, L^{(1)}_{qu,s}, \ldots , L^{(1)}_{q^{J-1}u,s}$, on top of each other, and summing over all possible combinations of outgoing paths. That is, the vertex weight $L^{(J)}_{u,s}(i_1,j_1;i_2,j_2)$ can be represented as a convex combination of products of the $J=1$ vertex weights with varying spectral parameters. We refer to [CP16b, Theorem 3.15], [BP18a, Section 5], or [BW22, Appendix B] for details. We also remark that formula (2.8) for the fused weights is essentially due to [Man14], and the fusion itself dates back to [KRS81].

Thanks to Proposition 2.1, we can view each vertex weight $L^{(J)}_{u,s}(i_1,j_1;i_2,j_2)$ with fixed incoming path counts $(i_1,j_1)$ as a probability distribution on all possible combinations of outgoing paths $(i_2,j_2)$. In Sect. 2.3 below, we use this probabilistic interpretation to build stochastic particle systems out of the vertex weights $L^{(J)}_{u,s}$.

Let us also make two remarks on the fusion procedure which was mentioned in the proof of Proposition 2.1.

#### Remark 2.2

*(Fusion)* **1.** The fused weights $L^{(J)}_{u,s}$ (2.8) are manifestly rational in $q^J$. Therefore, $q^J$ may be treated as an independent parameter and, moreover, may be specialized to a complex number not necessarily from the set $q^{\mathbb {Z}_{\ge 1}}=\left\{ q,q^2,q^3,\ldots \right\}$. This analytic continuation preserves the sum to one property (2.10) for the vertex weights $L^{(J)}_{u,s}(i_1,j_1;i_2,j_2)$ when summed over $i_2,j_2\ge 0$ (the path counts $i_1,j_1,i_2,j_2$ are always assumed to be nonnegative integers). Note however that the nonnegativity of the vertex weights $L^{(J)}_{u,s}$ has to be checked separately after such a continuation.

**2.**: For $J=1$, the weights $L_{u,s}^{(1)}$ may also be viewed as a stochastic fusion of the stochastic six vertex weights along the vertical edges. The latter arise from $L^{(1)}_{u,s}(i_1,j_1;i_2,j_2)$ by taking the specialization $s=q^{-\frac{1}{2}}$, which forces the path counts $i_1,i_2 \in \left\{ 0,1 \right\}$ (in addition to the constraint $j_1,j_2 \in \left\{ 0,1 \right\}$ due to $J=1$). Note that $s=q^{-\frac{1}{2}}$ falls outside of $(-1,0]$, contradicting the assumption (2.7), but one readily checks that the vertex weights $L^{(1)}_{u,\hspace{1pt}q^{-1/2}}$ are also nonnegative for $q\in [0,1)$ and $u\ge q^{-\frac{1}{2}}$.

The vertex weights $L^{(J)}_{u,s}$ generalize the *q*-beta-binomial distribution $\varphi _{q,\mu ,\nu }$ described in Sect. 2.1, and reduce to it in two cases. First, setting $u=s$, we have [Bor17, Proposition 6.7]:

2.12 $$\begin{aligned} L^{(J)}_{s,s}(i_1,j_1;i_2,j_2)= \textbf{1}_{i_1+j_1=i_2+j_2} \cdot \textbf{1}_{j_2\le i_1} \cdot \varphi _{q,q^Js^2,s^2}(j_2\mid i_1). \end{aligned}$$

In order to make (2.12) nonnegative, we should treat $\mu =q^Js^2$ as a parameter independent of $\nu$ (with $q^J$ not from $q^{\mathbb {Z}_{\ge 1}}$), and require that $0\le \mu \le 1$ and $\nu \le \mu$, as in the first case in (2.5). This analytic continuation is necessary since the substitution $u=s$ falls outside of the parameter range (2.7).

Second, in the limit as $i_1,i_2\rightarrow +\infty$, we have (e.g., see [BMP21, Appendix A.2]):

2.13 $$\begin{aligned} L^{(J)}_{u,s}(\infty ,j_1;\infty ,j_2)= \varphi _{q,suq^J,su}(j_2\mid \infty ). \end{aligned}$$

Here $j_2\in \mathbb {Z}_{\ge 0}$ is arbitrary, (2.13) does not depend on $j_1$, and the path conservation property disappears. The weights (2.13) are nonnegative for $J\in \mathbb {Z}_{\ge 1}$; see (2.6). Another choice to make (2.13) nonnegative is to take an analytic continuation with $su\ge 0$ and $\mu =q^Jsu$ treated as an independent parameter with $q^J\notin q^{\mathbb {Z}_{\ge 1}}$.

### Particle systems

We define two state spaces for two versions of our Markov dynamics.

#### Definition 2.3

The *vertex model state space* is

2.14 $$\begin{aligned} \mathscr {G}:=\biggl \{ \textbf{g}=(g_1,g_2,\ldots ):g_i\in \mathbb {Z}_{\ge 0},\ \sum _{i=1}^{\infty }g_i<\infty \biggr \}. \end{aligned}$$

The last condition means that only finitely many of the $g_i$’s are nonzero.

The *exclusion process state space* is

2.15 $$\begin{aligned} \mathscr {X}:=\Bigl \{ \textbf{x}= \left( x_1>x_2>x_3>\ldots \right) :x_i\in \mathbb {Z}, \ \, x_n=-n \ \text {for all sufficiently large { n}} \Bigr \}.\nonumber \\ \end{aligned}$$

We view $\textbf{x}$ as a particle configuration in $\mathbb {Z}$, which is empty far to the right and densely packed far to the left. In other words, every $\textbf{x}\in \mathscr {X}$ differs from the distinguished *step configuration* $\textbf{x}_{step}:=\left\{ -1>-2>-3>\ldots \right\}$ by finitely many particle jumps to the right by one, when a particle may only jump to an unoccupied location.

#### Definition 2.4

*(Gap-particle transformation).* Let the (well-known) bijection $\mathscr {X} \rightarrow \mathscr {G}$ with $\textbf{x} \mapsto \textbf{g}$ be defined as

**Fig. 7:**
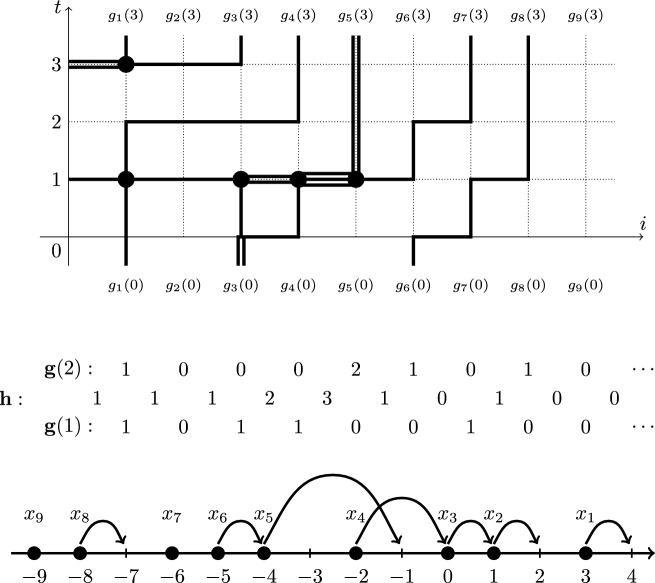
Top: A path ensemble corresponding to the time evolution of $\textbf{g}(t)$ for $t\in \left\{ 0,1,2,3 \right\}$. Middle: Update $\textbf{g}(1)\rightarrow \textbf{g}(2)$ and the corresponding quantities $h_i$, $i\ge 0$. Bottom: the corresponding exclusion process update $\textbf{x}(1)\rightarrow \textbf{x}(2)$, where the particle configuration $\textbf{x}(1)$ is shown, and the arrows represent sequential particle jumps

2.16 $$\begin{aligned} g_i=x_{i}-x_{i+1}-1,\qquad i\ge 1; \end{aligned}$$

see Fig. 7 for an illustration. Additionally, we use the convention $g_0=x_0=+\infty$, which extends (2.16) to $i=0$.

We refer to (2.16) as the *gap-particle transformation*. Note, in particular, that the distinguished step configuration of particles, $\textbf{x}_{step}$, corresponds to the empty configuration $\textbf{g}_{step}:=(0,0,\ldots )$. In the special case, when the updates are parallel and not sequential, this is the same as the zero range process (ZRP) / ASEP transformation, e.g., see [Pov13].

We are now in a position to describe the *fused stochastic higher spin six vertex* (*FS6V*) model $\textbf{g}(t)$ and its *exclusion process* counterpart, $\textbf{x}(t)$. Both models were introduced in [CP16b] for homogeneous parameters $u_i\equiv u$, $s_i\equiv s$, and their inhomogeneous versions were considered in [BP18a]. Here and throughout the rest of the section, $t\in \mathbb {Z}_{\ge 0}$ stands for discrete time in $\textbf{g}(t)$.

The time-homogeneous Markov process $\{\textbf{g}(t)\}_{t\in \mathbb {Z}_{\ge 0}}$ on $\mathscr {G}$ depends on two sequences of parameters

2.17 $$\begin{aligned} \textbf{s}=(s_0,s_1,s_2,\ldots ),\quad s_i\in (-1,0]; \qquad \textbf{u}=(u_0,u_1,u_2,\ldots ),\quad u_i\in [0,+\infty ), \nonumber \\ \end{aligned}$$

as well as on the parameters $q\in [0,1)$ and $J \in \mathbb {Z}_{\ge 1}$, as in (2.7). For convergence reasons discussed in Lemma 2.6 below, we assume that

2.18 $$\begin{aligned} \frac{(-s_i)(u_i-s_i)}{1-u_is_i}<1-\varepsilon <1 \end{aligned}$$

for some fixed $\varepsilon >0$ and all *i* large enough. For future use, let us write $(\textbf{u},\textbf{s})\in \mathcal {T}$ if the parameters satisfy the conditions (2.17)–(2.18).

#### Remark 2.5

One readily sees that if $-1<s_i< 0$ and $u_i>0$, then $\frac{(-s_i)(s_i-u_i)}{1-s_iu_i}< 1$. The condition (2.18) is stronger than (2.17) in that it does not allow these ratios to get arbitrarily close to 1 as *i* goes to infinity.

Let us describe how to randomly update the FS6V model $\textbf{g}(t)$ in (discrete) time *t*. Fix time $t\in \mathbb {Z}_{\ge 0}$, set $\textbf{g}=\textbf{g}(t)\in \mathscr {G}$, and let $\textbf{g}'=\textbf{g}(t+1)\in \mathscr {G}$ be the random update. The update is independent of time *t* and occurs as follows:

2.19 $$\begin{aligned} g_i' = g_i+h_{i-1}-h_i, \qquad i=1,2,\ldots , \end{aligned}$$

so that $h_i \in \left\{ 0,1,\ldots ,J \right\}$, with $i\in \mathbb {Z}_{\ge 0}$, are random variables that are sampled sequentially using the stochastic vertex weights $L^{(J)}_{u_i,s_i}$ for $i=0,1,2,\ldots$. Namely, $h_0$ is sampled from the probability distribution $L^{(J)}_{u_0,s_0}(\infty ,0;\infty ,h_0)$ (2.13). Then sequentially for $i=1,2,\ldots$, given $h_{i-1}$ and $g_i$, we sample the pair $(g_i',h_i)$ with $g_i'+h_i=g_i+h_{i-1}$ from the probability distribution $L^{(J)}_{u_i,s_i}(g_i,h_{i-1};g_i',h_{i})$ (2.8). Below, in Lemma 2.6, we show that eventually the update terminates, making it well-defined.

#### Lemma 2.6

We have $h_i=0$ for all *i* large enough for the update (2.19) with probability 1.

#### Proof

We know that $g_i=0$ for all *i* large enough. Since $L^{(J)}_{u,s}(0,0;0,0)=1$, it suffices to note that all the probabilities of the form $L^{(J)}_{u_i,s_i}(0,j;0,j)=\frac{s_i^{2j}(u_i/s_i;q)_j}{(s_iu_i;q)_j}$, where $j \in \left\{ 0,1,\ldots ,J \right\}$, are bounded away from 1 uniformly in $j\ge 1$ and sufficiently large *i*, due to (2.18). This means that once *i* becomes large enough so that all further $g_i$’s are zero, then with probability 1 the quantities $h_i$ eventually decrease to zero, and the update terminates. $\square$

After the sequential update over $i=0,1,\ldots$ terminates according to Lemma 2.6, we have reached the next state $\textbf{g}'=\textbf{g}(t+1)$.

The trajectory of the Markov process $\left\{ \textbf{g}(t) \right\} _{t\in \mathbb {Z}_{\ge 0}}$ may be viewed as a random path ensemble in the quadrant $\mathbb {Z}_{\ge 1}^{2}$. Namely, the initial condition $\textbf{g}(0)$ corresponds to the paths entering the quadrant from below, and the quantities $h_0$ sampled at each time moment $t\ge 1$ determine the paths entering from the left. The configuration $\textbf{g}(t)\in \mathscr {G}$ describes the paths crossing the horizontal line at height $t+\frac{1}{2}$. The random update from $\textbf{g}(t)$ to $\textbf{g}(t+1)$ determines the horizontal path counts at height $t+1$. See Fig. 7 for an illustration.

Denote by $T_{\textbf{u},\textbf{s}}$ the one-step Markov transition operator for the FS6V process $\{\textbf{g}(t)\}_{t\in \mathbb {Z}_{\ge 0}}$ on $\mathscr {G}$. This operator also depends on the parameters *q* and *J*, but we suppress this in the notation. In the literature on solvable lattice models (for instance, [Bax07]), $T_{\textbf{u},\textbf{s}}$ is often referred to as the transfer matrix. Our transfer matrix is a Markov transition operator since the model is stochastic. Similar stochasticity of transfer matrices was first observed in [GS92].

Finally, let us describe the Markov process $\{\textbf{x}(t)\}_{t\in \mathbb {Z}_{\ge 0}}$ on the space $\mathscr {X}$ induced by the FS6V process $\left\{ \textbf{g}(t) \right\}$ via the gap-particle transformation (2.16). The random update from $\textbf{x}(t)$ to $\textbf{x}(t+1)$ is described as follows. First, the rightmost particle $x_1$ jumps to the right by a random distance $h_0$ sampled from $L^{(J)}_{u_0,s_0}(\infty ,0;\infty ,h_0)$ (2.13). Then, sequentially for $i=1,2,\ldots$, the particle $x_{i+1}$ jumps to the right by a random distance $h_i$ sampled from

$$\begin{aligned} L^{(J)}_{u_i,s_i}(x_i(t)-x_{i+1}(t)-1,h_{i-1};x_{i}(t+1)-x_{i+1}(t+1)-1,h_i), \end{aligned}$$

given that $x_{i}$ has jumped by the distance $h_{i-1}$. Note that the upper bound for the distance of each particle’s jump is equal to the parameter *J*.

Due to the path conservation, we see that the dynamics of $\textbf{x}(t)$ satisfies the exclusion rule, that is, a particle may only jump to an unoccupied location. Consequently, the strict order of the particles $x_1>x_2>\ldots$ is preserved throughout the dynamics. We also note that the jump of each particle $x_i$ at time *t* is governed by the parameters $(u_{i-1},s_{i-1})$ as well as the locations $x_i(t)$, $x_{i-1}(t)$, and $x_{i-1}(t+1)$.

We denote the one-step Markov transition operator of the process $\{\textbf{x}(t)\}_{t\in \mathbb {Z}_{\ge 0}}$ on $\mathscr {X}$ by $\tilde{T}_{\textbf{u},\textbf{s}}$. It is the image of the FS6V model operator $T_{\textbf{u},\textbf{s}}$ on $\mathscr {G}$ under the gap-particle transformation (2.16). Throughout the paper we adopt the same convention for all Markov transition operators: *A* on $\mathscr {G}$ corresponds to $\tilde{A}$ on $\mathscr {X}$.

## Yang-Baxter Equation and Cross Vertex Weights

The vertex weights $L^{(J)}_{u,s}$ (2.8) satisfy the Yang-Baxter equation, and this makes the stochastic processes from Sections 2 very special, i.e., integrable. In short, the Yang-Baxter equation determines the (local) action of swapping two consecutive vertex weights in the FS6V model. This swapping action is represented by introducing a cross-vertex that is dragged across the vertex weights; see Fig. 9.

### Vertical Yang-Baxter equation

There are several possible Yang-Baxter equations that the vertex weights $L^{(J)}_{u,s}$’s may satisfy. For our purposes, we focus on the Yang-Baxter equation that may be represented by vertically dragging a cross vertex through two consecutive horizontal vertex weights.

Let $s_1,s_2\in (-1,0]$ and $z\ge 0$ be three parameters. Define the cross vertex weights as follows:

3.1 $$\begin{aligned} R_{z,s_1,s_2}(i_1,i_2;j_1,j_2) :=L^{(I_1)}_{s_1z,s_2}(j_1,i_2;i_1,j_2), \end{aligned}$$

with the right side given by (2.8) so that $I_1$ is determined by the identity$s_1=q^{-I_1/2}$. Here, we treat $q^{-I_1/2}$ as an independent parameter which enters $R_{z,s_1,s_2}$ in a rational manner, according to Remark 2.2. Explicitly, we have

**Fig. 8:**
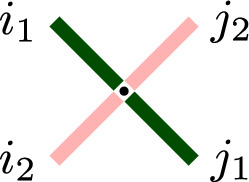
The weights $R_{z,s_1,s_2}$ (3.1) are attached to the cross vertices, i.e., vertices drawn on the lattice rotated by $45^\circ$. The path counts $i_1,i_2,j_1,j_2$ are as shown in the figure

3.2 $$\begin{aligned} R_{z,s_1,s_2}(i_1,i_2;j_1,j_2)= &  \textbf{1}_{j_1+i_2=i_1+j_2} \frac{(-s_1z)^{j_1}q^{ \frac{1}{2} j_1(j_1+2i_2-1)} s_2^{i_2+j_2-i_1}(zs_1 s_2^{-1};q)_{j_2- j_1}}{(q;q)_{i_1} (zs_1s_2;q)_{i_1+j_2} (s_1^{-2}q^{1-i_2};q)_{i_2-j_2}}\nonumber \\ &  \times _{4}\bar{\phi }_3\left( \begin{matrix} q^{-i_1};q^{-j_1},zs_1^{-1}s_2,q z^{-1}s_1^{-1}s_2\\ s_2^{2},q^{1+j_2-j_1},s_1^{-2}q^{1-i_1-j_2}\end{matrix} \bigg |\, q,q\right) , \end{aligned}$$

where $i_1,i_2,j_1,j_2$ are arbitrary nonnegative integers. The path conservation property in (3.1) means that $R_{z,s_1,s_2}(i_1,i_2;j_1,j_2)$ vanishes unless $i_1+j_2=i_2+j_1$. See Fig. 8 for an illustration.

The vertex weights $L^{(J)}_{u,s}$ and the cross vertex weights $R_{z,s_1,s_2}$ satisfy the following Yang-Baxter equation:

#### Proposition 3.1

[Yang-Baxter equation]. Fix the path counts $i_1,j_1 \in \left\{ 0,1,\ldots ,J \right\}$, $i_2,i_3,j_2,j_3\in \mathbb {Z}_{\ge 0}$ and the parameters $u_1,u_2,s_1,s_2$. Then, we have

3.3 $$\begin{aligned} \begin{aligned}&\sum _{k_1,k_2,k_3} R_{\frac{u_2}{u_1},s_1,s_2}(j_3,k_2;k_3,j_2) \hspace{1pt}L^{(J)}_{u_1,s_1}(i_2,i_1;k_2,k_1) \hspace{1pt}L^{(J)}_{u_2,s_2}(i_3,k_1;k_3,j_1) \\&\quad = \sum _{k_1,k_2,k_3} L^{(J)}_{u_2,s_2}(k_3,i_1;j_3,k_1)\hspace{1pt}L^{(J)}_{u_1,s_1}(k_2,k_1;j_2,j_1)\hspace{1pt}R_{\frac{u_2}{u_1},s_1,s_2}(k_3,i_2;i_3,k_2). \end{aligned} \end{aligned}$$

**Fig. 9:**
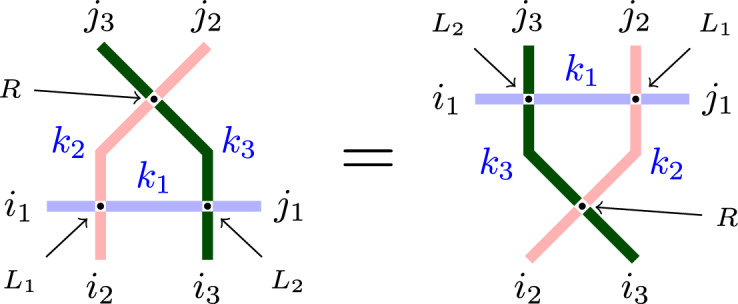
The Yang-Baxter equation (3.3) is the equality of partition functions of the two configurations in the figure, where the boundary path counts $i_1,i_2,i_3,j_1,j_2,j_3$ are fixed, and the summation is over all possible configurations of paths occupying the internal edges. These internal path counts are $k_1,k_2,k_3$. The vertex weights are $L_i=L^{(J)}_{u_i,s_i}$, and $R=R_{\frac{u_2}{u_1},s_1,s_2}$

See Fig. 9 for an illustration. The sums in (3.3) are over $k_1 \in \left\{ 0,1,\ldots ,J \right\}$ and $k_2,k_3\in \mathbb {Z}_{\ge 0}$. However, both sum are finite due to the path conservation properties, making the Yang-Baxter equation (3.3) an identity between rational functions.

Observe that the cross vertex weights $R_{\frac{u_2}{u_1},s_1,s_2}$ entering the Yang-Baxter equation (3.3) do not depend on the parameter *J* and only depend on the parameters $u_1,u_2$ through their ratio.

#### Proof of Proposition 3.1

The Yang-Baxter equation (3.3) follows by fusion from the simpler case when the paramters are $s_1=s_2=q^{-1/2}$ and $I_1=1$. This simpler case of the Yang-Baxter equation may be checked through direct computations. Moreover, the latter equation essentially coincides with [BMP21, Proposition A.1], up to the specialization of their parameter *s* into $q^{-J/2}$ and a gauge transformation making the vertex weights $w_{u,s}$ stochastic. Note that the cross vertex weights $r_{u/v}$ in [BMP21, Proposition A.1] are already stochastic (i.e. satisfy the sum to one property). Then, our fused Yang-Baxter equation (3.3) follows from [BMP21, Proposition A.3] (which is essentially a fusion of [BMP21, Proposition A.1]), up to a gauge transformation and path complementation $i\mapsto I-i$.

Alternatively, the fused Yang-Baxter equation with stochastic vertex weights implying (3.3) is a color-blind case of the master Yang-Baxter equation coming from $U_q(\widehat{\mathfrak {sl}_n})$ obtained in [BM16]; see [BW22, (C.1.2)]. $\square$

### Nonnegativity

From Proposition 2.1, we see that the cross vertex weights $R_{z,s_1,s_2}$, defined by (3.1), satisfy the sum to one property

3.4 $$\begin{aligned} \sum _{i_1,j_2=0}^{\infty } R_{z,s_1,s_2}(i_1,i_2;j_1,j_2)=1 \end{aligned}$$

for any fixed $i_2,j_1\in \mathbb {Z}_{\ge 0}$. Moreover, if the vertex weights $R_{z,s_1,s_2}$ are nonnegative, the vertex weights define a probability distribution. This distribution is on the top paths $(i_1, j_2)$ of a cross vertex for any fixed bottom paths $(i_2,j_1)$, see Fig. 8. Below, we show that the cross vertex weights are nonnegative under a suitable restriction of the parameters.

#### Proposition 3.2

If $q\in [0,1)$, $s_1,s_2\in (-1,0)$, and $0\le z\le \min \bigl \{ \frac{s_1}{s_2}, \frac{s_2}{s_1},\frac{q}{s_1s_2} \bigr \}$, then

$$\begin{aligned} R_{z,s_1,s_2}(i_1,i_2;j_1,j_2)\ge 0 \end{aligned}$$

for all $$i_1,j_1,i_2,j_2\in \mathbb {Z}_{\ge 0}$$.

#### Proof

First, assume that $i_1\le i_2$, which is equivalent to $j_1\le j_2$. Rewrite (3.2) via the ordinary *q*-hypergeometric series $_4\phi _3$ (2.9):

3.5 $$\begin{aligned} \begin{aligned}&R_{z,s_1,s_2}(i_1,i_2;j_1,j_2)= \textbf{1}_{j_1+i_2=i_1+j_2} \frac{(-s_1z)^{j_1}q^{ \frac{1}{2} j_1(j_1+2i_2-1)} s_2^{i_2+j_2-i_1}(zs_1 s_2^{-1};q)_{j_2-j_1}}{(q;q)_{i_1} (zs_1s_2;q)_{i_1+j_2} (s_1^{-2}q^{1-i_2};q)_{i_2-j_2}} \\ &\quad \times (s_2^2;q)_{i_1} (q^{1+j_2-j_1};q)_{i_1} (s_1^{-2}q^{1-i_1-j_2};q)_{i_1} \cdot _{4}\phi _3\left( \begin{matrix} q^{-i_1},q^{-j_1},zs_1^{-1}s_2,q z^{-1}s_1^{-1}s_2\\ s_2^{2},q^{1+j_2-j_1},s_1^{-2}q^{1-i_1-j_2}\end{matrix} \bigg |\, q,q\right) . \end{aligned}\nonumber \\ \end{aligned}$$

Note that $s_2^{i_2+j_2-i_1} = (-1)^{j_1} s_2^{2j_2}(-s_2)^{-j_1}$. Then, the following part of the prefactor

$$\begin{aligned} \frac{ (-s_1z)^{j_1} s_2^{2j_2}(-s_2)^{-j_1} q^{\frac{1}{2}j_1(j_1+2i_2-1)} (s_2^2;q)_{i_1} (q^{1+j_2-j_1};q)_{i_1} (zs_1 s_2^{-1};q)_{j_2-j_1} }{ (q;q)_{i_1} (zs_1s_2;q)_{i_1+j_2} } \end{aligned}$$

is clearly nonnegative under our conditions. Additionally, observe that

$$\begin{aligned} (-1)^{j_1}\frac{(s_1^{-2}q^{1-i_1-j_2};q)_{i_1}}{(s_1^{-2}q^{1-i_2};q)_{i_2-j_2}} =(-1)^{j_1}\,(s_1^{-2}q^{1-j_1-i_2};q)_{j_1}\ge 0, \end{aligned}$$

as all factors in the above *q*-Pochhammer symbol are nonpositive, and there are $j_1$ of them.

Thus, it remains to establish the nonnegativity of the *q*-hypergeometric series $_4\phi _3$ in (3.5). We use the nonnegativity result in the proof of [BMP21, Proposition A.8] which is based on Watson’s transformation formula [GR04, (III.19)]. That proof essentially established the nonnegativity of

3.6 $$\begin{aligned} _{4}\phi _3\left( \begin{matrix} q^{-\textsf{i}_2},q^{-\textsf{i}_1},- q / (\textsf{s}\xi ), -\textsf{s}\theta \\ -\textsf{s}/\xi , q^{1+\textsf{j}_2-\textsf{i}_2},-\theta q^{1-\textsf{i}_1-\textsf{j}_2} /\textsf{s}\end{matrix} \bigg |\, q,q\right) , \end{aligned}$$

where $\textsf{i}_2+\textsf{j}_1=\textsf{i}_1+\textsf{j}_2$, $\textsf{i}_2\le \textsf{j}_2$ (so $\textsf{i}_1\le \textsf{j}_1$), and the parameters satisfy

3.7 $$\begin{aligned} q\in (0,1),\qquad \textsf{s}\in (-\sqrt{q},0), \qquad \xi ,\theta \in [-\textsf{s},-\textsf{s}^{-1}]. \end{aligned}$$

Indeed, one can check that the prefactor

3.8 $$\begin{aligned} \frac{\textsf{s}^{\textsf{j}_2} (-\theta q^{1-\textsf{i}_1-\textsf{j}_2}/\textsf{s};q)_{\textsf{i}_2}}{ (-q / (\textsf{s}\xi );q)_{\textsf{i}_1-\textsf{j}_1} } \end{aligned}$$

in front of $_4\phi _3$ in [BMP21, (A.24)] is already nonnegative. Namely, for $\textsf{i}_1=\textsf{j}_2=0$ this prefactor is 1. For all other values of $\textsf{i}_1,\textsf{j}_2$ we see that $\textsf{s}<0$, $-\theta q^{1-\textsf{i}_1-\textsf{j}_2+l}/\textsf{s}\ge 1$ for $0\le l\le \textsf{i}_2-1$. Using (2.2) we have $(-q / (\textsf{s}\xi );q)_{\textsf{i}_1-\textsf{j}_1}^{-1} =(-1 / (\textsf{s}\xi );1/q)_{\textsf{j}_1-\textsf{i}_1}$, and note that $-q^{-l}/(\textsf{s}\xi )\ge 1$ for $l\ge 0$. Thus, (3.8) is a product of $\textsf{j}_2+\textsf{i}_2+\mathsf {j_1}-\mathsf {i_1}=2\textsf{j}_2$ nonpositive factors, and hence is nonnegative.

Now, (3.6) matches the $_4\phi _3$ function in (3.5) when $\textsf{i}_1=i_1$, $\textsf{i}_2=j_1$, $\textsf{j}_1=i_2$, $\textsf{j}_2=j_2$, and

$$\begin{aligned} \textsf{s}=-\sqrt{z s_1s_2}, \qquad \xi =\sqrt{zs_1s_2^{-3}},\qquad \theta =\sqrt{zs_1^{-3}s_2}. \end{aligned}$$

Rewriting conditions (3.7) on $\textsf{s},\xi ,\theta$ in terms of $z,s_1,s_2$, we arrive at the desired result for $i_1\le i_2$.

For the remaining case $i_1>i_2$, one can check that $R_{z,s_1,s_2}$ satisfies

3.9 $$\begin{aligned} R_{z,s_1,s_2}(i_1,i_2;j_1,j_2)=z^{j_2-i_2}\frac{s_2^{i_2+j_2}}{s_1^{i_1+j_1}} \,R_{z,s_2,s_1}(j_2,j_1;i_2,i_1). \end{aligned}$$

Thus, the case $i_1>i_2$ reduces to case $i_1 \le i_2$ since the prefactor is nonnegative. This establishes the result for all cases. $\square$

### Specialization at $q=0$

The expression for the cross vertex weights $R_{z,s_1,s_2}$ simplify considerably when $q=0$. Let us denote the specialization of the vertex weight at $q=0$ as follows

3.10 $$\begin{aligned} R^{(0)}_{z,s_1,s_2}(i_1,i_2;j_1,j_2) = R_{z,s_1,s_2}(i_1,i_2;j_1,j_2)\big \vert _{q=0}. \end{aligned}$$

Additionally, introduce the notation

3.11 $$\begin{aligned} \begin{aligned}&{\widehat{R}}^{(0)}_{z,s_1,s_2}(i_1,i_2;j_1,j_2) :=\textbf{1}_{i_1+j_2=i_2+j_1} \hspace{1pt}z^{j_1}(s_1s_2)^{-j_1} s_2^{2j_2} \\ &\quad \times \biggl ( \frac{(1-zs_1 s_2^{-1}\textbf{1}_{j_2>j_1})(1-s_2^2\textbf{1}_{i_1>0}) (1-s_1^2\textbf{1}_{i_2=0}\textbf{1}_{j_1>0})}{1-zs_1s_2\textbf{1}_{i_1+j_2>0}} \\&\quad - \textbf{1}_{i_1=i_2>0}\textbf{1}_{j_1>0} \hspace{1pt}\frac{(-s_1)(s_2z-s_1)}{1-zs_1s_2} \biggr ). \end{aligned} \end{aligned}$$

We express the specialization at $q=0$ in terms of $\widehat{R}^{(0)}_{z,s_1,s_2}$:

#### Proposition 3.3

We have

3.12 $$\begin{aligned} R^{(0)}_{z,s_1,s_2}(i_1,i_2;j_1,j_2) = {\left\{ \begin{array}{ll} {\widehat{R}}^{(0)}_{z,s_1,s_2}(i_1,i_2;j_1,j_2),& \text {if }\,\, j_1\le j_2;\\ z^{j_2-i_2}s_2^{i_2+j_2}s_1^{-i_1-j_1}{\widehat{R}}^{(0)}_{z,s_2,s_1} (j_2,j_1;i_2,i_1),& \text {if} \,\, j_1\ge j_2, \end{array}\right. }\nonumber \\ \end{aligned}$$

with the weights on the left and right side of the equation given by (3.10) and (3.11).

#### Proof of Proposition 3.3

Throughout the proof we assume that $j_1+i_2=i_1+j_2$ due to the path conservation property. We have

3.13 $$\begin{aligned} \begin{aligned}&R_{z,s_1,s_2}(i_1,i_2;j_1,j_2) = \sum _{k=0}^{\min (i_1,j_1)} \frac{(-s_1z)^{j_1}q^{ \frac{1}{2} j_1(j_1+2i_2-1)} s_2^{i_2+j_2-i_1}(zs_1 s_2^{-1};q)_{j_2-j_1}}{(q;q)_{i_1} (zs_1s_2;q)_{i_1+j_2} (s_1^{-2}q^{1-i_2};q)_{i_2-j_2}} \frac{q^k}{(q;q)_k} (q^{-i_1};q)_k \\&\hspace{20pt}\times (q^{-j_1};q)_k \bigl (\tfrac{zs_2}{s_1};q\bigr )_k \bigl (\tfrac{qs_2}{zs_1};q\bigr )_k (s_2^2q^k;q)_{i_1-k} (q^{1+j_2-j_1+k};q)_{i_1-k} (s_1^{-2}q^{1-i_1-j_2+k};q)_{i_1-k}. \end{aligned}\nonumber \\ \end{aligned}$$

Setting $q=0$ eliminates all the terms in the sum containing a positive power of *q*. If there are only positive powers of *q*, then the whole sum vanishes. Note that there are no negative powers of *q*, which follows from the fusion construction of $R_{z,s_1,s_2}$, see the proof of Proposition 3.1.

Thus, it remains to check how the powers of *q* are balanced in each term in (3.13). We have, for example, $(q;q)_{i_1}\big \vert _{q=0}=1$ and $(zs_1s_2;q)_{i_1+j_2}\big \vert _{q=0}=1$ for all $i_1,j_2$. One sees that all the factors we need to consider in detail are as follows:

3.14 $$\begin{aligned} \frac{ q^{ \frac{1}{2} j_1(j_1+2i_2-1)+k} (zs_1 s_2^{-1};q)_{j_2-j_1}}{(s_1^{-2}q^{1-i_2};q)_{i_2-j_2}} (q^{-i_1};q)_k (q^{-j_1};q)_k (s_1^{-2}q^{1-i_1-j_2+k};q)_{i_1-k}. \nonumber \\ \end{aligned}$$

Next, the factor $(zs_1 s_2^{-1};q)_{j_2-j_1}$ yields the power $q^{\frac{1}{2}(j_1-j_2)(j_1-j_2+1)}$ for $j_2<j_1$, see (2.2), and so on. Overall, one can check that the total power of *q* in (3.14) is equal to

3.15 $$\begin{aligned} \textbf{1}_{j_2<j_1}\genfrac(){0.0pt}1{j_1-j_2+1}{2} + \tfrac{1}{2} k(k-1) + k(i_2-i_1) \end{aligned}$$

If $j_1\le j_2$, then (3.15) equal to zero in the following cases:

- either $$k=0$$;
- or $$i_1=i_2$$ and $$k=1$$.

We obtain (3.11) by the contribution of the two cases above. Setting $q=0$ and $k=0$ in the sum in (3.13), we get the first summand in (3.11). For $i_1=i_2$, we get the additional second summand in (3.11) coming from the term with $k=1$. This proves (3.12) for $j_1\le j_2$. We use the symmetry (3.9) to obtain the result when $j_1\ge j_2$ This completes the proof. $\square$

We extend the nonnegativity result of Proposition 3.2 for the specialization at $q=0$. Note that Proposition 3.2 restricts *z* to 0 for $q=0$. Due to this, we need to independently find a range of parameters $(z,s_1,s_2)$ for which the weights $R^{(0)}_{z,s_1,s_2}$ are nonnegative:

#### Proposition 3.4

If $q=0$, $s_1,s_2\in (-1,0)$, $0\le z\le \min \{\frac{s_1}{s_2},\frac{s_2}{s_1}\}$, and $s_1^2+s_2^2 \le 1+zs_1s_2$, then

$$\begin{aligned} R^{(0)}_{z,s_1,s_2}(i_1,i_2;j_1,j_2)\ge 0 \end{aligned}$$

for all $$i_1,j_1,i_2,j_2\in \mathbb {Z}_{\ge 0}$$.

#### Proof

For $i_1\ne i_2$, one can check that conditions $s_1,s_2\in (-1,0)$ and $0\le z\le \min \{\frac{s_1}{s_2},\frac{s_2}{s_1}\}$ are enough for nonnegativity since only the first summand in (3.11) is present. When $i_1=i_2$, in the second summand in (3.11) we have $s_2z-s_1\ge 0$, so the second summand is negative. Combining it with the first summand leads to the additional condition $s_1^2+s_2^2 \le 1+zs_1s_2$. $\square$

### Specialization to *q*-beta-binomial cross vertex weights

For $q>0$, the cross vertex weights $R_{\frac{u_2}{u_1},s_1,s_2}$ (3.2) have a complicated *q*-hypergeometric expression, even when $J=1$ and the lattice vertex weights $L^{(J)}_{u_i,s_i}$ entering the Yang-Baxter equation (3.3) are explicit rational functions (see (2.11)). There are several ways to specialize the cross vertex parameters to simplify $R_{\frac{u_2}{u_1},s_1,s_2}$. For instance, one may take finite spin specializations by setting, in the simplest case, $s_1=s_2=q^{-\frac{1}{2}}$. However, this specialization would bound the gap sizes in the higher spin exclusion process $\textbf{x}(t)$ defined in Sect. 2.3 and, as a result, we will not consider this specialization here. Instead, we distinguish a specialization reducing $R_{\frac{u_2}{u_1},s_1,s_2}$ to the *q*-beta-binomial distribution:

#### Proposition 3.5

If $u_2/u_1=s_2/s_1$, then the cross vertex weights in the Yang-Baxter equation in Proposition 3.1 are simplified as

3.16 $$\begin{aligned} R_{\frac{s_2}{s_1},s_1,s_2} (i_1,i_2;j_1,j_2) = \textbf{1}_{i_1+j_2=i_2+j_1} \cdot \textbf{1}_{j_2\le j_1} \cdot \varphi _{q,s_2^2/s_1^2,s_2^2}(j_2\mid j_1), \end{aligned}$$

where $\varphi _{q,s_2^2/s_1^2,s_2^2}$ is the *q*-beta-binomial distribution (2.3).

#### Proof

This is a combination of (3.1) and the reduction of the weights $L^{(J)}_{s,s}$ (2.12). Here we set $q^{I_1}=s_1^{-2}$. $\square$

Recall (2.5) and note that $R_{\frac{s_2}{s_1},s_1,s_2} (i_1,i_2;j_1,j_2)\ge 0$ for all $i_1,i_2,j_1,j_2\in \mathbb {Z}_{\ge 0}$ if

3.17 $$\begin{aligned} s_1,s_2 \in (-1,0],\qquad |s_2|\le |s_1|. \end{aligned}$$

We see that the specialization $z=s_2/s_1$ extends the range of nonnegativity of the cross vertex weights compared to the conditions of Proposition 3.2. In particular, the condition $s_2^2\le q$ is dropped.

#### Definition 3.6

Let $\mathcal {R}$ be the range of parameters so that $(z,s_1,s_2)\in \mathcal {R}$ if

- either $q\in (0,1)$, $s_1,s_2\in (-1,0)$, and $0\le z\le \min \bigl \{ \frac{s_1}{s_2}, \frac{s_2}{s_1},\frac{q}{s_1s_2} \bigr \}$ as in Proposition 3.2;
- or $q=0$, $s_1,s_2\in (-1,0)$, $0\le z\le \min \{\frac{s_1}{s_2},\frac{s_2}{s_1}\}$, and $s_1^2+s_2^2 \le 1+zs_1s_2$ as in Proposition 3.4;
- or $z=s_2/s_1$ and $s_1,s_2\in (-1,0]$ with $|s_2|\le |s_1|$ as in (3.17).

Note that the cross vertex weights $R_{z,s_1,s_2}(i_1,i_2;j_1,j_2)$ are nonnegative for all $(z,s_1,s_2) \in \mathcal {R}$.

Let us describe the probabilistic interpretation of the specialization $R_{\frac{s_2}{s_1},s_1,s_2}(i_1,i_2;j_1, j_2)$ viewed as the distribution of the top paths $(i_1,j_2)$ given the bottom paths $(i_2,j_1)$, see Fig. 8 for an illustration. From (3.16) we see that $j_1$ paths coming from southeast randomly split into $j_2$ and $j_1-j_2$ according to the *q*-beta-binomial distribution $\varphi _{q,s_2^2/s_1^2,s_2^2}(j_2\mid j_1)$. Then $j_2$ paths continue in the northeast direction, while $j_1-j_2$ paths turn in the northwest direction. All the southwest $i_2$ paths simply continue in the northwest direction, so that $i_1=i_2+j_1-j_2$.

### Limit to infinitely many paths

We will also need a limit of the cross vertex weights $R_{z,s_1,s_2}(i_1,i_2;j_1,j_2)$ as the number of southwest incoming paths $i_2$ grows to infinity:

#### Proposition 3.7

Let $j_1,j_2\in \mathbb {Z}_{\ge 0}$. We have $\lim \limits _{L\rightarrow +\infty }R_{z,s_1,s_2}(L,L+j_2-j_1;j_1,j_2) = R^{\textrm{bdry}}_{z,s_1,s_2}(j_1,j_2)$, where $R^{\textrm{bdry}}_{z,s_1,s_2}(j_1,j_2)$ is, by definition, equal to

3.18 $$\begin{aligned} \frac{(-1)^{j_1}q^{\frac{1}{2}j_1(j_1-1)}s_2^{2j_2}(s_1z/s_2;q)_{j_2-j_1}}{(q;q)_{j_2}(s_2^2;q)_{j_1}}\hspace{1pt}\frac{(s_2^2;q)_{\infty }}{(zs_1s_2;q)_{\infty }} \, _{3}\bar{\phi }_2 \left( \begin{matrix} q^{-j_1};zs_2/s_1, q^{j_2-j_1}zs_1/s_2\\ q^{j_2-j_1+1},q^{1-j_1}z/(s_1s_2) \end{matrix} \bigg |\, q,q \right) .\nonumber \\ \end{aligned}$$

Here $_{3}\bar{\phi }_2$ is the regularized (terminating) *q*-hypergeometric series (2.9). Moreover, for any fixed $j_1\in \mathbb {Z}_{\ge 0}$ we have

3.19 $$\begin{aligned} \sum _{j_2=0}^{\infty } R^{\textrm{bdry}}_{z,s_1,s_2}(j_1,j_2)=1. \end{aligned}$$

#### Proof

We apply the Sears’ transformation formula [GR04, (III.15)] to $_{4}{\phi }_3$ in $R_{z,s_1,s_2}$ (3.2). After necessary simplifications, we obtain a terminating *q*-hypergeometric series $_{4}{\phi }_3$:

3.20 $$\begin{aligned} \begin{aligned}&R_{z,s_1,s_2}(i_1,i_2;j_1,j_2)= \frac{ s_2^{2j_2-j_1} z^{j_1}s_1^{-j_1}(s_1s_2/z;q)_{j_1} (zs_1 s_2^{-1};q)_{j_2-j_1} (s_2^2;q)_{i_1} (q^{1+j_2-j_1};q)_{i_1}}{(q;q)_{i_1} (s_2^2;q)_{j_1} (z s_1s_2;q)_{i_2} } \\ &\hspace{90pt}\times \textbf{1}_{j_1+i_2=i_1+j_2}\cdot _{4}\phi _3\left( \begin{matrix} q^{-j_1},zs_2s_1^{-1},q^{j_2-j_1}zs_1s_2^{-1}, q^{i_2+1}\\ q^{1+j_2-j_1},q^{1-j_1}zs_1^{-1}s_2^{-1},q^{i_2}zs_1s_2\end{matrix} \bigg |\, q,q\right) . \end{aligned}\nonumber \\ \end{aligned}$$

Note that in $_{4}\phi _3$ there is one upper and one lower parameter that each contain $q^{i_2}$ as a factor. Then, sending $i_2$ to infinity eliminates these upper and lower parameters in $_{4}\phi _3$, producing $_3\phi _2$ with the remaining parameters. The prefactor in front of $_3\phi _2$ readily leads to that in (3.18); recall the regularization (2.9). This completes the proof of the first claim.

For the second claim, recall that the quantities $R_{z,s_1,s_2} (i_2+j_1-j_2,i_2;j_1,j_2)$ (3.20) sum to one over $0\le j_2\le i_2+j_1$ for any fixed $(i_2,j_1)$, see (3.4) (for $j_2>i_2+j_1$, these quantities vanish). We would like to take the limit as $i_2\rightarrow +\infty$ inside the sum

3.21 $$\begin{aligned} \sum _{j_2\ge 0}R_{z,s_1,s_2} (i_2+j_1-j_2,i_2;j_1,j_2). \end{aligned}$$

Assuming $i_2$ is large and $j_2\ge j_1$ is fixed, we can estimate uniformly in $i_2$:

$$\begin{aligned} \left| \frac{ (s_2^2;q)_{i_2+j_1-j_2} (q^{1+j_2-j_1};q)_{i_2+j_1-j_2}}{(q;q)_{i_2+j_1-j_2} (z s_1s_2;q)_{i_2}}\right| \le \textrm{const}\cdot \left| \frac{(s_2^2;q)_{\infty } (q^{1+j_2-j_1};q)_{\infty }}{(q;q)_{\infty } (z s_1s_2;q)_{\infty }} \right| , \end{aligned}$$

which is bounded. For the *k*-th term in the terminating *q*-hypergeometric series (with $0\le k\le j_1$), we have $\left| \frac{(q^{i_2 + 1}; q)_k}{(q^{i_2}zs_1s_2; q)_k} \right| \le \textrm{const}$ (we only included the factors depending on $i_2$). Now that $i_2$ is accounted for, all the $j_2$-dependent factors (coming from the prefactor and from the *k*-th term in the *q*-hypergeometric sum) are estimated as

$$\begin{aligned} \left| s_2^{2j_2-j_1} (zs_1 s_2^{-1};q)_{j_2-j_1} \right| \le \textrm{const}\cdot s_2^{2j_2},\qquad \left| \frac{ (q^{j_2 - j_1}zs_1s_2^{-1}; q)_k }{(q^{1 + j_2 - j_1}; q)_k }\right| \le \textrm{const}. \end{aligned}$$

Therefore, thanks to the factor $s_2^{2j_2}$, the tail $j_2\ge M$ of the sum (3.21) is bounded above uniformly in $i_2$ by $\textrm{const}\cdot (1-\varepsilon )^{M}$ for some $\varepsilon >0$. This implies that we can take the limit $i_2\rightarrow +\infty$ inside the sum (3.21), resulting in (3.19). $\square$

Let us write down the specializations of $R^{\textrm{bdry}}_{z,s_1,s_2}(j_1,j_2)$ to $q=0$ and to the the *q*-beta-binomial distribution, similar to Sects. 3.3 and 3.4. For $q=0$, we have the specialization (3.11)–(3.12) for finite $i_1,i_2$. Then, by taking the limit as $i_1,i_2$ increase arbitrarily large, we obtain the following specialization:

3.22 $$\begin{aligned} R^{\textrm{bdry},(0)}_{z,s_1,s_2}(j_1,j_2) :=R^{\textrm{bdry}}_{z,s_1,s_2}(j_1,j_2) \big \vert _{q=0} = {\left\{ \begin{array}{ll} {\widehat{R}}^{\textrm{bdry},(0)}_{z,s_1,s_2}(j_1,j_2),& \text {if }\,\,j_1\le j_2; \\[4pt] \dfrac{s_2^{2j_2}s_1^{-2j_1}}{1-s_1^2\textbf{1}_{j_2=0}} \widehat{R}^{\textrm{bdry},(0)}_{z,s_2,s_1}(j_2,j_1),& \text {if}\,\, j_1> j_2, \end{array}\right. }\nonumber \\ \end{aligned}$$

where

3.23 $$\begin{aligned} \widehat{R}^{\textrm{bdry},(0)}_{z,s_1,s_2}(j_1,j_2) :=\frac{z^{j_1}(s_1s_2)^{-j_1} s_2^{2j_2}}{1-zs_1s_2} \bigl ( (1 - zs_1s_2^{-1} \textbf{1}_{j_2>j_1})(1- s_2^2)+(z s_1 s_2 -s_1^2) \textbf{1}_{j_1=j_2 >0} \bigr ).\nonumber \\ \end{aligned}$$

In the *q*-beta-binomial specialization $z=s_2/s_1$, the limiting weights $R^{\textrm{bdry}}_{z,s_1,s_2}(j_1,j_2)$ are exactly the same as pre-limit ones (see Proposition 3.5):

3.24 $$\begin{aligned} R^{\textrm{bdry}}_{\frac{s_2}{s_1},s_1,s_2}(j_1,j_2)= \textbf{1}_{j_2\le j_1} \cdot \varphi _{q,s_2^2/s_1^2,s_2^2}(j_2\mid j_1). \end{aligned}$$

Indeed, setting $z=s_2/s_1$ eliminates the dependence of $R_{z,s_1,s_2}(i_1,i_2;j_1,j_2)$ on $i_1,i_2$. Then, one can immediately take the limit $i_1,i_2\rightarrow +\infty$ as in Proposition 3.7.

We also observe that the limiting weights $R^{\textrm{bdry}}_{z,s_1,s_2}$ are nonnegative if $(z,s_1,s_2)\in \mathcal {R}$, see Definition 3.6.

## Intertwining Relations

In this section we present our first main result, the intertwining (or quasi-commutation) relations for the Markov transition operator of the stochastic higher spin six vertex model. Here we discuss the result at the level of Markov operators based on vertex weights. Then, in Sects. 5 and 6 below, we present its specializations to exclusion processes on the line, such as *q*-TASEP and TASEP, and to the Schur vertex model.

### Swap operators

Recall the state spaces $\mathscr {G}$ and $\mathscr {X}$, from Definition 2.3, and the Markov operators $T_{\textbf{u},\textbf{s}}$ and $\tilde{T}_{\textbf{u},\textbf{s}}$ on $\mathscr {G}$ and $\mathscr {X}$, respectively and described in Sect. 2.3, coming from the stochastic higher spin six vertex model $\textbf{g}(t)$ and its exclusion process counterpart $\textbf{x}(t)$. These Markov operators depend on two sequences of parameters $(\textbf{u},\textbf{s})\in \mathcal {T}$ (i.e. parameters satisfying the conditions given by (2.17)– (2.18)).

Here we define new Markov operators on $\mathscr {G}$ based on the stochastic cross vertex weights $R_{z,s_1,s_2}$ and $R^{\textrm{bdry}}_{z,s_1,s_2}$. Via the gap-particle correspondence, these operators also define the corresponding Markov operators on $\mathscr {X}$.

#### Definition 4.1

*(Markov swap operators).* For $n\ge 1$ and $(z,s_1,s_2)\in \mathcal {R}$ (the range of parameters given in Definition 3.6), let $P^{(n)}_{z,s_1,s_2}$ be the Markov operator acting on $\mathscr {G}$ by randomly changing the coordinates $(g_{n-1},g_n)$ into $(g_{n-1}',g_n')$ sampled from the cross vertex weights

4.1 $$\begin{aligned} {\left\{ \begin{array}{ll} R_{z,s_1,s_2}(g_{n-1}',g_{n-1};g_n,g_n'),& n\ge 2;\\ R^{\textrm{bdry}}_{z,s_1,s_2}(g_n,g_n'),& n=1. \end{array}\right. } \end{aligned}$$

In particular, we always have $g_{n-1}'+g_n'=g_{n-1}+g_n$. The boundary case $n=1$ is consistent with our usual agreement $g_0=g_0'=+\infty$. By definition, the operator $P^{(n)}_{z,s_1,s_2}$ does not change all other coordinates $g_j$, where $j\ne n-1,n$.

Additionally, let ${\tilde{P}}^{(n)}_{z,s_1,s_2}$ be the corresponding operator on $\mathcal {X}$ induced via the gap-particle duality. This operator randomly moves the particle $x_n$ to a new location $x_n'$ based on the locations of the neighboring particles $x_{n-1}$ and $x_{n+1}$, with probabilities coming from (4.1) via the gap-particle transformation (Definition 2.4).

The Yang-Baxter equation implies an intertwining relation between $P^{(n)}$ and *T*. This relation may also be called a (quasi-)commutation between the operators. The following statement is an immediate consequence of Proposition 3.1:

#### Proposition 4.2

Fix $n\ge 1$. Let $(\textbf{u},\textbf{s})\in \mathcal {T}$ be such that $\bigl ( \frac{u_n}{u_{n-1}},s_{n-1},s_n \bigr )\in \mathcal {R}$. Then, we have

**Fig. 10:**
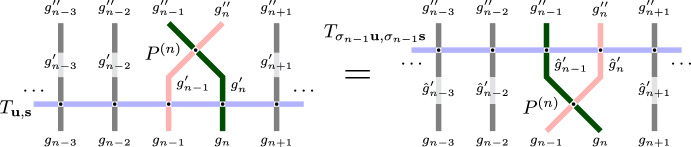
Relation (4.2)–(4.3) between Markov operators $T_{\textbf{u},\textbf{s}}$ and $P^{(n)}=P^{(n)}_{u_n/u_{n-1},s_{n-1},s_n}$

4.2 $$\begin{aligned} T_{\textbf{u},\textbf{s}} \hspace{1pt}P^{(n)}_{u_n/u_{n-1},s_{n-1},s_n} = P^{(n)}_{u_n/u_{n-1},s_{n-1},s_n} T_{\sigma _{n-1}\textbf{u},\sigma _{n-1}\textbf{s}}, \end{aligned}$$

where $\sigma _{n-1}=(n-1,n)$ is the *n*-th elementary transposition in the symmetric group acting on $\mathbb {Z}_{\ge 0}$. The same identity holds if all the operators in (4.2) are replaced by their counterparts acting in the space $\mathscr {X}$. See Fig. 10 for an illustration.

Thus, we have established Proposition 1.6 from the Introduction.

#### Remark 4.3

In (4.2) and throughout the paper, we adopt the convention that the product of Markov operators follows the order of their action on measures. In particular, on arbitrary delta measures $\delta _{\textbf{g}}$, where $\textbf{g}\in \mathscr {G}$ is fixed, the identity (4.2) is expanded as follows

4.3 $$\begin{aligned} \sum _{\textbf{g}'\in \mathscr {G}} T_{\textbf{u},\textbf{s}} (\textbf{g},\textbf{g}') \hspace{1pt}P^{(n)}_{u_n/u_{n-1},s_{n-1},s_n} (\textbf{g}',\textbf{g}'') = \sum _{\hat{\textbf{g}}'\in \mathscr {G}} P^{(n)}_{u_n/u_{n-1},s_{n-1},s_n} (\textbf{g},\hat{\textbf{g}}') \hspace{1pt}T_{\sigma _{n-1}\textbf{u},\sigma _{n-1}\textbf{s}} (\hat{\textbf{g}}',\textbf{g}''),\nonumber \\ \end{aligned}$$

for any fixed $\textbf{g},\textbf{g}''\in \mathscr {G}$; see Fig. 10 for an illustration. Note that both sums in (4.3) are finite due to the path conservation property, which is built into the operator $P^{(n)}$.

### Shift operator

Let us now consider a product of the operators $P^{(n)}$ over all $n\ge 1$ with parameters chosen in such a way that the iterated intertwining relations (4.2) lead to the shifting in $\textbf{u}, \textbf{s}$:

4.4 $$\begin{aligned} &  \textsf{sh}:=\ldots \sigma _2 \sigma _1 \sigma _0, \qquad \textsf{sh}(u_0,u_1,u_2,\ldots )= (u_1,u_2,\ldots ), \qquad \nonumber \\  &  \quad \textsf{sh}(s_0,s_1,s_2,\ldots )= (s_1,s_2,\ldots ). \end{aligned}$$

That is, we swap the parameters $(u_0,s_0)$ first with $(u_1,s_1)$, then with $(u_2,s_2)$, and so on all the way to infinity. As a result, the parameters $(u_0,s_0)$ disappear, leading to the shift (4.4). First, we need certain assumptions on the parameters:

#### Definition 4.4

Denote by $\mathcal {B}$ the space of sequences $(\textbf{u},\textbf{s})$ as in (2.17) such that:

- $$\bigl (\frac{u_n}{u_0},s_0,s_n\bigr )\in \mathcal {R}$$ for all $$n\ge 1$$;
- There exists $\varepsilon >0$ such that
  4.5 $$\begin{aligned} \frac{(-s_n)(u_ns_0-u_0s_n)}{u_0-s_0s_nu_n}<1-\varepsilon <1 \end{aligned}$$
  for all sufficiently large *n*.

Similarly to Remark 2.5, the condition $\bigl (\frac{u_n}{u_0},s_0,s_n\bigr )\in \mathcal {R}$ implies that the ratios in (4.5) are already $\le 1$. However, these ratios must be bounded away from 1 as *n* grows.

We are now in a position to define the Markov operator on the space $\mathscr {G}$ which acts on the stochastic higher spin six vertex model $T_{\textbf{u},\textbf{s}}$ by shifting the parameter sequences.

#### Definition 4.5

*(Markov shift operator).* Let $(\textbf{u},\textbf{s})\in \mathcal {B}$. We define the operator $B_{\textbf{u},\textbf{s}}$ on $\mathscr {G}$ by

**Fig. 11:**
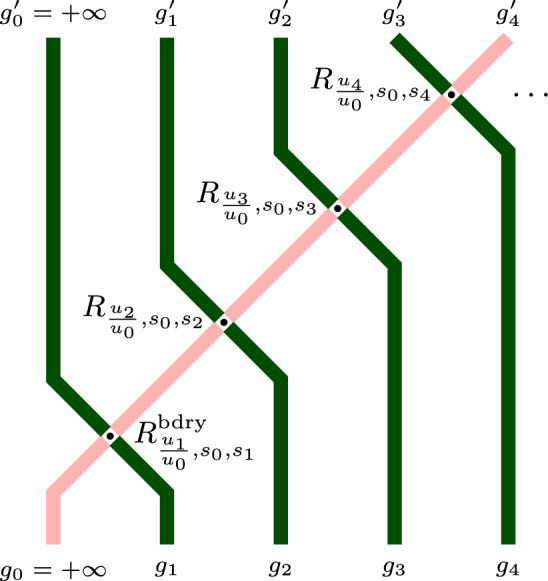
The path configuration whose weight is the matrix element $B_{\textbf{u},\textbf{s}}(\textbf{g},\textbf{g}')$ of the Markov shift operator from Definition 4.5

4.6 $$\begin{aligned} B_{\textbf{u},\textbf{s}} :=P^{(1)}_{\frac{u_1}{u_0},s_0,s_1} P^{(2)}_{\frac{u_2}{u_0},s_0,s_2} P^{(3)}_{\frac{u_3}{u_0},s_0,s_3} \ldots \end{aligned}$$

(the order follows the action on measures, cf. Remark 4.3). See Fig. 11 for an illustration. By means of the gap-particle transformation (Definition 2.4), we also obtain a corresponding operator ${\tilde{B}}_{\textbf{u},\textbf{s}}$ acting in the space $\mathscr {X}$ of particle configurations.

#### Lemma 4.6

If $(\textbf{u},\textbf{s})\in \mathcal {B}$, then the shift operator $B_{\textbf{u},\textbf{s}}$ is well-defined.

#### Proof

Since $(u_n/u_0,s_0,s_n)\in \mathcal {R}$ for all $n\ge 1$, all vertex weights involved in the operators $P^{(n)}_{u_n/u_0,s_0,s_n}$ in the product (4.6) are nonnegative. Next, the second condition in (4.5) implies that no path escapes to infinity under the action of $B_{\textbf{u},\textbf{s}}$, similarly to the proof of Lemma 2.6. Indeed, we have

$$\begin{aligned} R_{\frac{u_n}{u_0},s_0,s_n}(0,j;0,j)=\frac{s_n^{2j}(u_ns_0/(u_0s_n);q)_{j}}{(u_ns_0s_n/u_0;q)_{j}}, \qquad j \in \mathbb {Z}_{\ge 0}, \end{aligned}$$

and due to (4.5), these quantities are bounded away from 1 uniformly in $j\ge 1$ for all *n* sufficiently large. This completes the proof. $\square$

The first main structural result is the next Theorem 4.7. It concerns the stochastic higher spin six vertex model $T_{\textbf{u},\textbf{s}}$ with general horizontal spin *J*, and shows how the operator $B_{\textbf{u},\textbf{s}}$ acts on it by shifting the parameter sequence. Below in Sect. 5, we consider specializations of Theorem 4.7 to simpler particle systems, which leads to known and new results. Most importantly, whereas the known results only applied to step initial conditions, the new results apply to stochastic particle systems started from a arbitrary initial condition.

#### Theorem 4.7

Let $$(\textbf{u},\textbf{s})\in \mathcal {T}\cap \mathcal {B}$$. Then

4.7 $$\begin{aligned} T_{\textbf{u},\textbf{s}} \hspace{1pt}B_{\textbf{u},\textbf{s}} = B_{\textbf{u},\textbf{s}} \hspace{1pt}T_{\textsf{sh}(\textbf{u}),\textsf{sh}(\textbf{s})}, \end{aligned}$$

where $\textsf{sh}$ is the shift (4.4). The same identity holds if all the operators in (4.7) are replaced (via the gap-particle transformation of Definition 2.4) by their corresponding counterparts acting on the space $\mathscr {X}$.

#### Proof

This is simply an iteration of Proposition 4.2. $\square$

Note that the intermediate summations, as in Remark 4.3, arising in the products in both sides of (4.7) are finite. Indeed, for any fixed $\textbf{g},\textbf{g}''\in \mathscr {G}$ there are only finitely many $\textbf{g}'\in \mathscr {G}$ such that $T_{\textbf{u},\textbf{s}}(\textbf{g},\textbf{g}') \hspace{1pt}B_{\textbf{u},\textbf{s}}(\textbf{g}',\textbf{g}'')>0$. Moreover, one can check similarly to the proofs of Lemmas 2.6 and 4.6 that under the condition $(\textbf{u},\textbf{s})\in \mathcal {T}\cap \mathcal {B}$, both products in (4.7) are well-defined as Markov operators in $\mathscr {G}$. That is, with probability 1, the product of the Markov operators does not allow paths to run off to infinity if $(\textbf{u},\textbf{s})\in \mathcal {T}\cap \mathcal {B}$.

## Application to *q*-Hahn TASEP and its Specializations

In this section we consider specializations of Theorem 4.7 to the *q*-Hahn TASEP [Pov13, Cor14], various *q*-TASEPs [SW98, BC14, BC15], and the usual TASEP. We recover the previously known results on parameter symmetry obtained in [PS22, Pet21], and extend them to arbitrary initial data. All Poisson-type limit transitions involved in this section are the same as in [Pet21], and therefore we only sketch the details of how discrete time Markov chains become continuous time Markov jump processes.

### *q*-Hahn Boson and *q*-Hahn TASEP

Let us first recall [BP18a, Section 6.6] how the stochastic higher spin six vertex model $T_{\textbf{u},\textbf{s}}$ from Sect. 2.3 specializes to the *q*-Hahn Boson system from [Pov13, Cor14]. This is achieved by setting

5.1 $$\begin{aligned} u_i=s_i\in (-1,0],\quad i\in \mathbb {Z}_{\ge 0},\qquad q^J=\gamma \in \bigl [1,\sup \nolimits _{i\ge 0} s_i^{-2}\bigr ). \end{aligned}$$

The stochastic vertex weights $L^{(J)}_{s_i,s_i}$ for all $i\ge 0$ are the *q*-beta-binomial weights $\varphi _{q,\gamma s_i^2,s_i^2}$ (2.3), see (2.12)–(2.13). These weights are nonnegative under the parameter assumptions (5.1). Thus, the resulting stochastic vertex model is called the (*stochastic*) *q-Hahn Boson system*. Denote its one-step Markov transition operator acting on $\mathscr {G}$ by $T_{\gamma ,\textbf{s}}^{\textrm{qHahn}}$, and the corresponding Markov transition operator acting on $\mathscr {X}$ (via the gap-particle transformation, see Definition 2.4) by ${\tilde{T}}_{\gamma ,\textbf{s}}^{\textrm{qHahn}}$. Throughout this section it is convenient to work in the exclusion process state space $\mathscr {X}$.

The stochastic particle system on $\mathscr {X}$ with Markov transition operator ${\tilde{T}}_{\gamma ,\textbf{s}}^{\textrm{qHahn}}$ is called the *q-Hahn TASEP*. In *q*-Hahn TASEP, the updates are performed *in parallel*, as opposed to the sequential update in the general case. That is, each particle $x_n$ jumps to the right independently of other particles by a random distance $h_{n-1}$, where $0\le h_{n-1}\le x_{n-1}-x_n-1$, with probability

5.2 $$\begin{aligned} \varphi _{q,\gamma s_{n-1}^2,s_{n-1}^2}(h_{n-1}\mid x_{n-1}-x_n-1), \qquad n=1,2,\ldots . \end{aligned}$$

Here, we use the notation $h_{n-1}$ in agreement with Sect. 2.3. For $n=1$, we have $x_0=+\infty$, so the jumping distribution is given by (2.4).

Let us denote the *q*-Hahn specialization of the swap operator (Definition 4.1) acting on the space $\mathscr {G}$ by $P^{(n),\textrm{qHahn}}_{s_{n-1},s_n}$, and its corresponding counterpart acting on the space $\mathscr {X}$ by $\tilde{P}^{(n),\textrm{qHahn}}_{s_{n-1},s_n}$. These operators involve the cross vertex weights

5.3 $$\begin{aligned} \begin{aligned} R^{\textrm{bdry}}_{\frac{s_1}{s_0},s_0,s_1}(g_1,g_1')&=\textbf{1}_{g_1'\le g_1}\cdot \varphi _{q,s_1^2/s_0^2,s_1^2}(g_1'\mid g_1),\\ R_{\frac{s_n}{s_{n-1}},s_{n-1},s_n}(g_{n-1}',g_{n-1};g_n,g_n')&= \textbf{1}_{g_{n-1}'+g_n'=g_{n-1}+g_n} \cdot \textbf{1}_{g_n'\le g_n}\cdot \varphi _{q,s_n^2/s_{n-1}^2,s_n^2}(g_n'\mid g_n), \end{aligned} \end{aligned}$$

where $n\ge 2$, which specialize to the *q*-beta-binomial distribution (Proposition 3.5). By (3.17), the operators $P^{(n),\textrm{qHahn}}_{s_{n-1},s_n}$ and $\tilde{P}^{(n),\textrm{qHahn}}_{s_{n-1},s_n}$ have nonnegative matrix elements if $s_{n-1},s_n\in (-1,0]$ and $|s_n|\le |s_{n-1}|$.

#### Remark 5.1

Setting $s_i=u_i$ is not the only way of making the cross vertex weights to take the simpler *q*-beta-binomial form. For instance, one could take $J\in \mathbb {Z}_{\ge 1}$ and require that $s_i/s_j=u_i/u_j$ for all *i*, *j*. We do not focus on this case in the current Sect. 5 since this section is devoted to extending existing results from [PS22, Pet21] on *q*-Hahn TASEP and its specializations. Results very similar to the ones below in Sect. 5 hold for the subfamily of stochastic higher spin six vertex models with $s_i/s_j=u_i/u_j$ for all *i*, *j*. We return to this subfamily in Sect. 8 below.

Proposition 4.2 extends the action of the *q*-Hahn swap operators from [Pet21] to general initial data. Let us fix some notation to formulate the result. Fix a discrete time $t\in \mathbb {Z}_{\ge 0}$ and a particle configuration $\textbf{y}\in \mathscr {X}$. Let $\textbf{x}(t)$ be the particle configuration of the *q*-Hahn TASEP at time *t* started from the initial particle configuration $\textbf{x}(0)=\textbf{y}$, with parameters $\textbf{s}=(s_0,s_1,s_2,\ldots )$, $-1<s_i\le 0$. Additionally, let $n\in \mathbb {Z}_{\ge 1}$ and assume that $|s_n|\le |s_{n-1}|$. Applying the swap operator $\tilde{P}^{(n),\textrm{qHahn}}_{s_{n-1},s_n}$ to the configuration $\textbf{x}(t)$ at time *t* moves a single particle $x_n(t)$ to a random new location $x_n'(t)$ with probability

5.4 $$\begin{aligned} \varphi _{q,s_n^2/s_{n-1}^2,s_{n}^2}(x_n'(t)-x_{n+1}(t)-1\mid x_n(t)-x_{n+1}(t)-1). \end{aligned}$$

Denote the resulting configuration by $\textbf{x}'(t)$.

#### Proposition 5.2

[Extension of [Pet21, Theorem 3.8] to general initial data]. Take the notation above. Then, the random configuration $\textbf{x}'(t)$ coincides in distribution with the configuration of the *q*-Hahn TASEP at time *t* started from a *random* initial configuration $\delta _{\textbf{y}}{\tilde{P}}^{(n),\textrm{qHahn}}_{s_{n-1},s_n}$ that evolves with the swapped parameters $\sigma _{n-1}\textbf{s}$, where $\sigma _{n-1}=(n-1,n)$ is the *n*-th elementary transposition.

#### Proof

This is the *q*-Hahn specialization of the intertwining relation (4.2) of Proposition 4.2 between the swap operator and the time evolution operator (applied *t* times). Note that this result is formulated for the space $\mathscr {X}$ of particle configurations in $\mathbb {Z}$. $\square$

The swap operator ${\tilde{P}}^{(n),\textrm{qHahn}}_{s_{n-1},s_n}$ preserves the step initial configuration $\textbf{y}=\textbf{x}_{step}$ due to the presence of the indicator $\textbf{1}_{g_n'\le g_n}$ in (5.3). Thus, the swap operator does not randomize the initial configuration for the *q*-Hahn TASEP started with $\textbf{x}_{step}$. In the case of step initial data, Proposition 5.2 was proven in [Pet21] (up to matching notation $\nu _i=s_{i-1}^2$) using exact formulas which are not readily available for general initial data. Finally, observe that $\tilde{P}^{(n),\textrm{qHahn}}_{s_{n-1},s_n}$ becomes the identity operator when $s_n=s_{n-1}$, and the statement of Proposition 5.2 in this case is trivial (while still true).

One may also specialize Theorem 4.7 to the case of *q*-Hahn TASEP. The result is a shift operator which acts by removing the parameter $s_0$ if $|s_0|\ge |s_n|$ for all $n\ge 1$. In a continuous time limit, this leads to an extension of [Pet21, Theorem 4.7] to general initial data. The original result [Pet21, Theorem 4.7] for the step initial data follows by observing that the *q*-Hahn specialization of the shift operator preserves $\textbf{x}_{step}$. To avoid cumbersome notation, in this paper we only consider the continuous limit for the case of *q*-TASEP, see Sects. 5.3 and 5.4 below.

### Intertwining relation for geometric *q*-TASEP

Let us now consider the limit of the *q*-Hahn TASEP leading to the discrete time *q*-TASEP:

5.5 $$\begin{aligned} s_n^2\rightarrow 0,\qquad \gamma s_n^2\rightarrow a_n\in (0,1),\qquad n=0,1,\ldots . \end{aligned}$$

This also implies that $s_n^2/s_{k}^2\rightarrow a_n/a_k$. Under this limit, the *q*-Hahn TASEP turns into the *discrete time geometric q-TASEP* introduced in [BC15]. During each time step in this process, each particle $x_n$, $n\in \mathbb {Z}_{\ge 1}$, jumps to the right independently of other particles by a random distance $h_{n-1}$ with probability (see (5.2))

5.6 $$\begin{aligned} &  \varphi _{q,a_{n-1},0}(h \mid g)= a_{n-1}^h (a_{n-1};q)_{g-h} \hspace{1pt}\frac{(q;q)_g}{(q;q)_h(q;q)_{g-h}}, \qquad \nonumber \\  &  h=h_{n-1},\ g=x_{n-1}-x_n-1. \end{aligned}$$

When $n=1$, we have $g=+\infty$, by agreement. Each $a_{n-1}$ may be viewed as the speed parameter attached to the particle $x_{n}$ in the *q*-TASEP. When $q=0$, the jumping distance (5.6) becomes $h_{n-1}=\min (\eta ,x_{n-1}-x_n-1)$, where $\eta \in \mathbb {Z}_{\ge 0}$ is a geometric random variable with ${\mathbb {P}}(\eta =k)=a_{n-1}^k(1-a_{n-1})$. Hence, the name “geometric”.

Observe that the geometric *q*-TASEP swap operator depends only on the ratio of the speed parameters, and involves the vertex weights $\varphi _{q,a_n/a_{n-1},0}$ similarly to (5.3)–(5.4). The swap operator and the *q*-TASEP evolution satisfy a relation similar to Proposition 5.2.

Let us further specialize the speed parameters in the *q*-TASEP by setting

5.7 $$\begin{aligned} a_k=\alpha r^{k},\qquad k\in \mathbb {Z}_{\ge 0}, \end{aligned}$$

where $\alpha ,r\in (0,1)$ are fixed. Denote the Markov transition operator of this *q*-TASEP acting on $\mathscr {X}$ by $\tilde{T}^{\textrm{qT}}_{\alpha ,r}$. Using Definition 4.5, let us also denote by ${\tilde{B}}^{\textrm{qT}}_{r}$ the corresponding shift operator. Note that it does not depend on $\alpha$ and involves the vertex weights $\varphi _{q,r^n,0}$, where $r^n=a_n/a_0$.

#### Proposition 5.3

Fix $t\in \mathbb {Z}_{\ge 0}$ and $\textbf{y}\in \mathscr {X}$. Let $\textbf{x}(t)$ be the configuration of the geometric *q*-TASEP $\tilde{T}^{\textrm{qT}}_{\alpha ,r}$ at time *t*, started from $\textbf{y}=\textbf{x}(0)$. Also, let $\textbf{x}'(t)$ be the configuration resulting from applying the shift operator $\tilde{B}^{\textrm{qT}}_{r}$ to $\textbf{x}(t)$. Then, $\textbf{x}'(t)$ coincides in distribution with the *q*-TASEP $\tilde{T}^{\textrm{qT}}_{\alpha r,r}$ at time *t* started from a random initial configuration $\delta _{\textbf{y}}\tilde{B}^{\textrm{qT}}_{r}$ and evolving with the modified parameters $a_k'=\alpha r^{k+1}$, $k\in \mathbb {Z}_{\ge 0}$.

In terms of the operators, the statement is equivalent to the following intertwining relation:

5.8 $$\begin{aligned} \bigl (\tilde{T}^{\textrm{qT}}_{\alpha ,r}\bigr )^t {\tilde{B}}^{\textrm{qT}}_{r} = {\tilde{B}}^{\textrm{qT}}_{r} \bigl ({\tilde{T}}^{\textrm{qT}}_{\alpha r,r}\bigr )^t, \end{aligned}$$

where the order of the operators is understood as in Remark 4.3, and $(\cdots )^t$ means raising to the nonnegative integer power *t*.

#### Proof of Proposition 5.3

This is a specialization of Theorem 4.7 formulated in terms of particle systems. Note that the operator ${\tilde{T}}^{\textrm{qT}}_{\alpha ,r}$ is well-defined for $a_k=\alpha r^k$, since $0<a_k<1$ for all *k*. Also, the shift operator $\tilde{B}^{\textrm{qT}}_{r}$ is well-defined by Lemma 4.6 since $a_k>a_{k+1}$ for all *k*, and the second condition in Definition 4.4 holds trivially if $u_i = s_i$ for all *i*. $\square$

### Limit to continuous time *q*-TASEP

Let us now take a Poisson-type limit to continuous time for the geometric *q*-TASEP ${\tilde{T}}^{\textrm{qT}}_{\alpha ,r}$. This is achieved by letting

5.9 $$\begin{aligned} \alpha \rightarrow 0, \qquad t=\lfloor (1-q)\textsf{t}/\alpha \rfloor , \end{aligned}$$

where $\textsf{t}\in \mathbb {R}_{\ge 0}$ is the new continuous time variable. Indeed, observe the expansion

5.10 $$\begin{aligned} \varphi _{q,\mu ,0}(h\mid g)= {\left\{ \begin{array}{ll} 1+O(\mu ),& h=0;\\[4pt] \dfrac{1-q^g}{1-q}\hspace{1pt}\mu +O(\mu ^2),& h=1;\\[7pt] O(\mu ^2),& h\ge 1, \end{array}\right. } \qquad \qquad \mu \rightarrow 0. \end{aligned}$$

This means that particles jump very rarely for small $\alpha$ in discrete time. Moreover, when a particle jumps, it jumps by one with much higher probability than any other distance greater than one. Then, by speeding up the time, the discrete jumping distributions $\varphi _{q,\alpha r^{n-1},0}$ (5.6) lead to independent exponential clocks. Therefore, under the resulting *continuous time q-TASEP* [BC14], each particle $x_n$ has an independent exponential clock of rate $r^{n-1}\left( 1-q^{g_n} \right)$, where $g_n=x_n-x_{n+1}-1$ (the factor $1-q$ in the rate in (5.10) is removed by the time scaling (5.9)). When the clock attached to the particle $x_n$ rings, this particle jumps by 1 to the right. Note that when $g_n=0$, the jump rate of $x_n$ is zero, which means that a particle cannot jump into an occupied location.

#### Remark 5.4

*(TASEP specialization,* $q=0$*).* The continuous time *q*-TASEP with the sequence of speeds $(1,r,r^2,\ldots )$ turns into the *TASEP* with these speeds when $q=0$. Under TASEP, each particle $x_n$ has an independent exponential clock with rate $r^{n-1}$. When a clock rings, the corresponding particle jumps to the right by one, provided that the destination is unoccupied. Otherwise, the jump of the particle is blocked.

Moreover, in the case $r=1$, we recover the well-known *homogeneous continuous time TASEP* in which the speeds of all particles are equal to 1.

Let us denote the continuous time Markov semigroup on $\mathscr {X}$ corresponding to the continuous time *q*-TASEP with particle speeds $(1,r,r^2,\ldots )$ by $\{\tilde{T}^{\textrm{qT}}_r(\textsf{t})\}_{\textsf{t}\in \mathbb {R}_{\ge 0}}$. In the case $r=1$, the process given by the semigroup $\tilde{T}^{\textrm{qT}}_1(\textsf{t})$ (which we will denote simply by ${\tilde{T}}^{\textrm{qT}}(\textsf{t})$) is the *homogeneous q-TASEP*, where all particles have speeds equal to 1.

Taking the continuous time limit (5.9) in (5.8), we get the following intertwining relations for any $m\in \mathbb {Z}_{\ge 1}$:

5.11 $$\begin{aligned} \tilde{T}^{\textrm{qT}}_r(\textsf{t}) \bigl ( {\tilde{B}}^{\textrm{qT}}_{r} \bigr )^{m} = \bigl ( {\tilde{B}}^{\textrm{qT}}_{r} \bigr )^{m}\hspace{1pt}\tilde{T}^{\textrm{qT}}_r(r^{m}\hspace{1pt}\textsf{t}). \end{aligned}$$

Indeed, shifting the sequence of speed parameters as $(1,r,r^2,\ldots )\mapsto (r^m,r^{m+1},r^{m+2}, \ldots )$ means slowing down all the particles by the factor $r^{m}$, which is equivalent to looking at the *q*-TASEP distribution at an earlier time $r^{m}\hspace{1pt}\textsf{t}$.

### Mapping *q*-TASEP back in time

We now aim to take one more Poisson-type limit in the intertwining relation (5.11). Let

5.12 $$\begin{aligned} r= 1-\varepsilon ,\qquad m=\lfloor \tau /\varepsilon \rfloor ,\qquad \varepsilon \searrow 0, \end{aligned}$$

where $\tau \in \mathbb {R}_{\ge 0}$ is a new continuous time parameter. Under (5.12), one readily sees that the *q*-TASEP Markov operators in both sides of (5.11) turn into the operators ${\tilde{T}}^{\textrm{qT}}(\textsf{t})$ and $\tilde{T}^{\textrm{qT}}(e^{-\tau } \hspace{1pt}\textsf{t})$, respectively. Recall that the latter two operators correspond the homogeneous *q*-TASEP where all particles move with homogeneous speed one.

Let us consider the limit of $\bigl ( {\tilde{B}}^{\textrm{qT}}_{r} \bigr )^{m}$. In particular, consider the cross vertex weights (5.3) in the limit $\varepsilon \rightarrow 0$ under the specialization (5.5) and (5.7). For any fixed $n\in \mathbb {Z}_{\ge 0}$, we have:

5.13 $$\begin{aligned} \varphi _{q,r^n,0}(g' \mid g)= {\left\{ \begin{array}{ll} \displaystyle \frac{n}{1-q^{g-g'}}\frac{(q;q)_g}{(q;q)_{g'}} \hspace{1pt}\varepsilon +O(\varepsilon ^2),& 0\le g'\le g-1; \\[8pt] 1-ng\hspace{1pt}\varepsilon +O(\varepsilon ^2),& g'=g. \end{array}\right. } \end{aligned}$$

Note that the quantity $r^n$ arises as $a_n/a_0$, see (5.7).

We have the following interpretation for the expansion (5.13). For small $\varepsilon$, the action of a single shift operator $\tilde{B}^{\textrm{qT}}_{r}$ does not change the particle configuration with high probability. Speeding up the time leads to exponential particle jumps with rates coming from the coefficients of the $\varepsilon$-terms in (5.13). In the limit regime (5.12) the operators $\bigl ( {\tilde{B}}^{\textrm{qT}}_{r} \bigr )^{m}$ on $\mathscr {X}$ converge into a continuous time Markov semigroup $\{{\tilde{B}}^{\textrm{qT}}(\tau )\}_{\tau \in \mathbb {R}_{\ge 0}}$ on $\mathscr {X}$, where the convergence is in the sense of matrix elements of Markov operators on $\mathscr {X}$.

We call the Markov semigroup ${\tilde{B}}^{\textrm{qT}}(\tau )$ on $\mathscr {X}$ the *backwards q-TASEP dynamics*. Under this dynamics, each particle $x_n$ has an independent exponential clock with rate $ng_n=n(x_n-x_{n+1}-1)$. When a clock rings, the corresponding particle $x_n$ instantaneously jumps backwards to a new location $x_n'<x_n$ with probability

5.14 $$\begin{aligned} \frac{1}{g_n(1-q^{x_n-x_n'})}\frac{(q;q)_{g_n}}{(q;q)_{g_n'}}, \qquad \text {where}\quad g_n=x_n-x_{n+1}-1,\quad g_n'=x_n'-x_{n+1}-1.\nonumber \\ \end{aligned}$$

Observe that for any configuration in $\mathscr {X}$, the sum of the jump rates of all possible particle jumps is finite, meaning that the backwards *q*-TASEP on $\mathscr {X}$ is well-defined.

#### Remark 5.5

In the TASEP specialization, when $q=0$ (cf. Remark 5.4), the probabilities (5.14) define a uniform distribution. Therefore, under the backwards dynamics, when the clock of the particle $x_n$ rings (with rate $n(x_n-x_{n+1}-1)$), this particle selects one of the following locations

$$\begin{aligned} \left\{ x_{n+1}+1,x_{n+1}+2,\ldots ,x_n-2,x_n-1 \right\} \end{aligned}$$

uniformly at random, and instantaneously jumps into the selected location. Thus, setting $q=0$ turns the backwards *q*-TASEP dynamics ${\tilde{B}}^{\textrm{qT}}(\tau )$ into the (*inhomogeneous*) *backwards Hammersley process* introduced in [PS22] (see Fig. 5 for an illustration).

Taking the Poisson-type limit (5.12) of the intertwining relation (5.11), we immediately obtain the main result of Sect. 5 (this is Theorem 1.7 from the Introduction):

#### Theorem 5.6

Let $\{{\tilde{T}}^{\textrm{qT}}(\textsf{t})\}_{\textsf{t}\in \mathbb {R}_{\ge 0}}$ and $\{{\tilde{B}}^{\textrm{qT}}(\tau )\}_{\tau \in \mathbb {R}_{\ge 0}}$ be the Markov semigroups of the homogeneous *q*-TASEP and the backwards *q*-TASEP on $\mathscr {X}$, respectively. Then

5.15 $$\begin{aligned} \tilde{T}^{\textrm{qT}}(\textsf{t}) \hspace{1pt}{\tilde{B}}^{\textrm{qT}}(\tau ) = {\tilde{B}}^{\textrm{qT}}(\tau ) \hspace{1pt}\tilde{T}^{\textrm{qT}}\bigl (e^{-\tau }\textsf{t}\bigr ) \qquad \text {for all }\,\, \textsf{t}, \tau \in \mathbb {R}_{\ge 0}. \end{aligned}$$

The same identity holds if all the operators are replaced (via the gap-particle transformation of Definition 2.4) by their counterparts acting in the vertex model space $\mathscr {G}$.

Theorem 5.6 may be reformulated equivalently in terms of stochastic particle systems on $\mathbb {Z}$. Fix $\textbf{y}\in \mathscr {X}$, and let $\textbf{x}(\textsf{t})$ denote the configuration of the homogeneous *q*-TASEP at time $\textsf{t}$ started with initial condition $\textbf{x}(0)=\textbf{y}$. Fix $\tau$, and run the backwards *q*-TASEP dynamics from the configuration $\textbf{x}(\textsf{t})$ for time $\tau$. Then, the distribution of the resulting configuration is the same as the distribution of the *q*-TASEP at time $e^{-\tau }\textsf{t}$ with *random* initial configuration $\delta _{\textbf{y}}{\tilde{B}}^{\textrm{qT}}(\tau )$.

We recover the $\nu =0$ case^4^ of [Pet21, Theorem 4.7] by setting the initial configuration $\textbf{y}$ is $\textbf{x}_{step}$. In particular, note that the configuration $\textbf{x}_{step}$ is fixed by $\tilde{B}^{\textrm{qT}}(\tau )$. The Theorem in [Pet21, Theorem 4.7] states that the backwards dynamics maps the distribution of *q*-TASEP with step initial data backwards in time, from $\textsf{t}$ to $e^{-\tau }\textsf{t}$, by applying the backwards *q*-TASEP for time $\tau$. Moreover, setting $q=0$ recovers [PS22, Theorem 1] for the homogeneous TASEP.

### Lax equation for *q*-TASEP and TASEP

We obtain a Lax type equation for the *q*-TASEP (and TASEP in the special case $q=0$), arising from identity (5.15) established in Theorem 5.6. Our computations in this subsection are informal, though we believe that the end results (5.17) and (5.18) become rigorous in appropriate spaces of functions. We believe that our Lax equation could be employed to study multipoint asymptotics of the *q*-TASEP and, in a scaling limit, lead to Kadomtsev-Petviashvili (KP) or Korteweg-de Vries (KdV) type equations recently derived in [QR22] for the KPZ fixed point process [MQR21]. We leave the asymptotic analysis of the Lax equation to future work.

Let $\tilde{\textsf{T}}$ and $\tilde{\textsf{B}}$ denote the infinitesimal generators of the *q*-TASEP and the backwards *q*-TASEP, respectively. Multiply both sides of (5.15) by $\tilde{T}^{\textrm{qT}}(\textsf{t}-e^{-\tau }\textsf{t})$ from the right. Using the semigroup property of $\tilde{T}^{\textrm{qT}}(\textsf{t})$, we obtain

$$\begin{aligned} {\tilde{T}}^{\textrm{qT}}(\textsf{t}) \hspace{1pt}{\tilde{B}}^{\textrm{qT}}(\tau ) \hspace{1pt}{\tilde{T}}^{\textrm{qT}}(\textsf{t}-e^{-\tau }\textsf{t}) = \tilde{B}^{\textrm{qT}}(\tau ) \hspace{1pt}\tilde{T}^{\textrm{qT}}\bigl (\textsf{t}\bigr ). \end{aligned}$$

Fix $\textsf{t}>0$, and differentiate this identity in $\tau$ at $\tau =0$. We obtain

$$\begin{aligned} {\tilde{T}}^{\textrm{qT}}(\textsf{t}) \bigl ( \tilde{\textsf{B}}+\textsf{t}\cdot \tilde{\textsf{T}} \bigr ) =\tilde{\textsf{B}}\hspace{1pt}{\tilde{T}}^{\textrm{qT}}(\textsf{t}). \end{aligned}$$

Dividing by $\textsf{t}$, rewrite this as

5.16 $$\begin{aligned} \tilde{T}^{\textrm{qT}}(\textsf{t}) \hspace{1pt}\tilde{\textsf{T}} = \left[ \tfrac{1}{\textsf{t}} \tilde{\textsf{B}}, \tilde{T}^{\textrm{qT}}(\textsf{t}) \right] , \end{aligned}$$

where $[\cdot ,\cdot ]$ is the commutator of operators. Using Kolmogorov (also called Fokker–Planck) equation, we can express the left-hand side as a derivative in $\textsf{t}$. Thus, we obtain

5.17 $$\begin{aligned} \frac{d}{d\hspace{1pt}\textsf{t}} \tilde{\textsf{T}}^{\textrm{qT}}(\textsf{t}) = \left[ \tfrac{1}{\textsf{t}} \tilde{\textsf{B}}, \tilde{T}^{\textrm{qT}}(\textsf{t}) \right] , \end{aligned}$$

a differential equation for the *q*-TASEP semigroup in the Lax form.

Let us apply the Lax equation to an arbitrary (sufficiently nice) function *F* on the space $\mathscr {X}$. Note that we have $\bigl (\tilde{\textsf{T}}^{\textrm{qT}}(\textsf{t}) F\bigr )(\textbf{y})= \mathbb {E}_{\textbf{y}}\left[ F(\textbf{x}(\textsf{t})) \right]$, where the expectation is with respect to the *q*-TASEP at time $\textsf{t}$ started from $\textbf{y}$, since $\tilde{\textsf{T}}^{\textrm{qT}}(\textsf{t})$ is a Markov semigroup. Then, from (5.16), we obtain the following:

5.18 $$\begin{aligned} \textsf{t}\, \mathbb {E}_{\textbf{y}}\bigl [ \bigl (\tilde{\textsf{T}}\hspace{1pt}F\bigr )(\textbf{x}(\textsf{t})) \bigr ] = \tilde{\textsf{B}} \hspace{1pt}\mathbb {E}_{\textbf{y}} \left[ F(\textbf{x}(\textsf{t})) \right] - \mathbb {E}_{\textbf{y}} \bigl [ \bigl (\tilde{\textsf{B}}\hspace{1pt}F\bigr )(\textbf{x}(\textsf{t})) \bigr ], \end{aligned}$$

where the operator $\tilde{\textsf{B}}$ on the right side acts on the expectation as a function in $\textbf{y}$, for the first term, and on the function *F*, for the second term.

Identity (5.18) generalizes [Pet21, Proposition 5.3] (and also [PS22, Proposition 7.1] when $q=0$) by allowing an arbitrary initial condition $\textbf{y}$. Indeed, if $\textbf{y}=\textbf{x}_{step}$, then $\tilde{\textsf{B}} \hspace{1pt}\mathbb {E}_{\textbf{y}} \left[ F(\textbf{x}(\textsf{t})) \right] =0$ because $\textbf{x}_{step}$ is an absorbing state for $\tilde{\textsf{B}}$. Thus, the combined generator $\textsf{t}\hspace{1pt}\tilde{\textsf{T}}+\tilde{\textsf{B}}$ satisfies

$$\begin{aligned} \mathbb {E}_{\textbf{x}_{step}} \bigl [ \bigl ( \textsf{t}\hspace{1pt}\tilde{\textsf{T}}F+\tilde{\textsf{B}}F \bigr )(\textbf{x}(\textsf{t})) \bigr ]=0, \end{aligned}$$

so the process with this combined generator preserves the time $\textsf{t}$ distribution of the *q*-TASEP started from the step initial configuration. This preservation of measure was proven in [Pet21] using contour integral formulas available for the *q*-TASEP distribution with the step initial configuration, and for $q=0$ in [PS22] using a different approach. Moreover, using duality, in [Pet21] it was shown that the process with the combined generator converges to its stationary distribution when started from an arbitrary initial configuration in $\mathscr {X}$.

## Application to Schur Vertex Model

The Schur vertex model studied in [KPS19] is the $J=1$, $q=0$ specialization of the stochastic higher spin six vertex model $\textbf{g}(t)$ defined in Sect. 2.3. The name “Schur” comes from the fact that some joint distributions in $\textbf{g}(t)$ are expressed through the Schur processes [KPS19, Theorem 3.5]. This model can be equivalently reformulated as a certain corner growth [KPS19, Section 1.2], and is also equivalent to the generalized TASEP of [DPPP12] and [Pov13], which appeared (in the form of tandem queues and first passage percolation models) already in [Woe05] and [Mar09]. Here we outline the specialization of the general shift operator from Sect. 4.2 to this model. We also observe that in contrast with the *q*-Hahn TASEP and its specializations, in the Schur vertex model the shift operator *does not* preserve the distinguished initial configuration $\textbf{g}_{step}$.

The Schur vertex model scales to a version of the TASEP in continuous inhomogeneous space [KPS19, Theorem 2.7]. It would be interesting to see how the shift operators behave under this scaling, but we do not pursue this analysis here.

### Schur vertex model

The *Schur vertex model* depends on the parameters $u_i,s_i$ as in (2.17). For simplicity, here we can take the parameters $s_i$ to be homogeneous, $s_i\equiv s\in (-1,0)$. Denote $\nu =s^2\in (0,1)$ and $-su_i=a_i\ge 0$. In term of these parameters, condition (2.18) means that the $a_i$’s should be uniformly bounded from above.

The transition probabilities in the Schur vertex model are the $q=0$ specializations of (2.11). They are given by

6.1 $$\begin{aligned} \begin{aligned}&L^{\textrm{Schur}}_{a_i,\nu }(0,0;0,0)=1, \qquad L^{\textrm{Schur}}_{a_i,\nu }(0,1;0,1)=\frac{\nu +a_i}{1+a_i}, \qquad \\ &L^{\textrm{Schur}}_{a_i,\nu }(0,1;1,0)=\frac{1-\nu }{1+a_i}; \\&L^{\textrm{Schur}}_{a_i,\nu }(g,0;g,0)= L^{\textrm{Schur}}_{a_i,\nu }(g,1;g+1,0)= \frac{1}{1+a_i},\qquad g\ge 1; \\&L^{\textrm{Schur}}_{a_i,\nu }(g,0;g-1,1)= L^{\textrm{Schur}}_{a_i,\nu }(g,1;g,1)= \frac{a_i}{1+a_i},\qquad g\ge 1. \end{aligned} \end{aligned}$$

Throughout this section it is convenient to work in the vertex model state space $\mathscr {G}$ (Definition 2.3). We interpret $\textbf{g}_i$ for each $i\in \mathbb {Z}_{\ge 1}$ as the number of particles at location *i*, where multiple particles per site are allowed. Let $T^{\textrm{Schur}}_{\nu ,\textbf{a}}$ denote the Markov transition operator for the Schur vertex model acting in $\mathscr {G}$.

Let us describe the dynamics for the Markov operator $T^{\textrm{Schur}}_{\nu ,\textbf{a}}$. At each time step, the stacks of particles are updated in parallel. First, each nonempty stack of particles $\textbf{g}_i(t)>0$ emits a single particle with probability $a_i / (1+a_i)$. Then, the emitted particle instantaneously travels to the right by a random distance $\min (\eta ,k+1)$, where $\eta$ is a random variable in $\mathbb {Z}_{\ge 1}$ with distribution

$$\begin{aligned} {\mathbb {P}}\left( \eta =j \right) = \frac{1-\nu }{1+a_{i+j}} \prod _{m=1}^{j-1}\frac{\nu +a_{i+m}}{1+a_{i+m}}, \qquad j\ge 1, \end{aligned}$$

and $k\ge 0$ is the number of empty stacks after $\textbf{g}_i(t)$, i.e. $\textbf{g}_{i+1}(t)=\ldots =\textbf{g}_{i+k}(t)=0$ and $\textbf{g}_{i+k+1}(t)>0$. If $\textbf{g}_i(t)$ is the rightmost nonempty stack, then $k=+\infty$.

### Shift operator for the Schur vertex model

Let $B^{\textrm{Schur}}_{\nu ,\textbf{a}}$ denote the Markov shift operator (Definition 4.5) for the Schur vertex model. It acts on the space $\mathscr {G}$ and involves the cross vertex weights $R^{\textrm{bdry},(0)}_{a_1/a_0,-\sqrt{\nu },-\sqrt{\nu }}$ (3.22) and $R^{(0)}_{a_n/a_0,-\sqrt{\nu },-\sqrt{\nu }}$ (3.12) for $n\ge 2$. For the nonnegativity of these weights, the parameters must satisfy the conditions in Proposition 3.4, which means

6.2 $$\begin{aligned} 2-\frac{1}{\nu }\le \frac{a_n}{a_0}\le 1,\qquad n\ge 1. \end{aligned}$$

Note that the lower bound on $a_n/a_0$ is restrictive only for $\nu > \frac{1}{2}$. The operator $B^{\textrm{Schur}}_{\nu ,\textbf{a}}$ is well-defined due to Lemma 4.6, since the condition (4.5) is automatic for our specialization of parameters. The next statement readily follows from Theorem 4.7:

#### Proposition 6.1

Let $\nu \in (0,1)$ and the parameters $a_n\ge 0$ be uniformly bounded from above and satisfy (6.2). Then

6.3 $$\begin{aligned} T^{\textrm{Schur}}_{\nu ,\textbf{a}}\hspace{1pt}B^{\textrm{Schur}}_{\nu ,\textbf{a}} = B^{\textrm{Schur}}_{\nu ,\textbf{a}}\hspace{1pt}T^{\textrm{Schur}}_{\nu ,\textsf{sh}(\textbf{a})}, \end{aligned}$$

where $\textsf{sh}$ is the shift of the sequence $\textbf{a}=(a_0,a_1,a_2,\ldots )$ as in (4.4).

In contrast with the *q*-Hahn TASEP and its specializations considered in Sect. 5 and in the previous papers [PS22] and [Pet21], the shift operator $B^{\textrm{Schur}}_{\nu ,\textbf{a}}$, for the Schur vertex model, *does not preserve* the distinguished empty configuration $\textbf{g}_{step}\in \mathscr {G}$:

#### Proposition 6.2

Let the parameters $\{a_n \}$ satisfy (6.2). Then the action of $B^{\textrm{Schur}}_{\nu ,\textbf{a}}$ on $\textbf{g}_{step}$ changes $\textbf{g}_{step}$ with positive probability.

#### Proof

From (3.22)–(3.23) we have

6.4 $$\begin{aligned} R^{\textrm{bdry},(0)}_{z,-\sqrt{\nu },-\sqrt{\nu }} (0,j)=\frac{\nu ^j(1-\nu )(1-z\textbf{1}_{j>0})}{1-\nu z}, \qquad j\in \mathbb {Z}_{\ge 0}. \end{aligned}$$

This means that applying the first operator $P^{(1)}$ (see (4.6)) to $\textbf{g}_{step}$ introduces a random number of paths according to the distribution (6.4) with $z=a_1/a_0$. These paths do not disappear after the application of the further operators $P^{(2)},P^{(3)},\ldots$ in (4.6) due to path conservation. Moreover, from (3.11)–(3.12) we have

6.5 $$\begin{aligned} R^{(0)}_{z,-\sqrt{\nu },-\sqrt{\nu }}(i,j;0,j-i) =\frac{\nu ^{j-i}(1-\nu \textbf{1}_{i>0})(1-z\textbf{1}_{i<j})}{1-\nu z\textbf{1}_{j>0}}, \qquad i\in {0,1,\ldots ,j }, \nonumber \\ \end{aligned}$$

where $z=a_n/a_0$ for $n\ge 2$. This implies that the operator $B^{\textrm{Schur}}_{\nu ,\textbf{a}}$ indeed does not preserve the distinguished empty configuration $\textbf{g}_{step}$. $\square$

**Part II Bijectivisation and Rewriting History**

In the second part, we describe how the intertwining relations obtained in the first part lead to couplings between trajectories of the stochastic vertex model (and the corresponding exclusion process) with different sequences of parameters. The passage from intertwining relations to couplings, a “bijectivisation”, is by now a well-known technique that originated in [DF90] and was later developed in the context of integrable stochastic particle systems in [BF14, BG09, BP16b, BP19]. Here, we apply a bijectivisation in a new setting leading to couplings of probability measures on trajectories under time evolution of integrable stochastic systems.

## Bijectivisation and Coupling of Trajectories. General Constructions

In this section, we return to the general setup of the fused stochastic higher spin six vertex model as in Sects. 2 to 4. We construct couplings between measures on trajectories of the stochastic higher spin six vertex model with different sequences of parameters by applying a bijectivisation [BP19] (also called a “probabilistic bijection”, e.g., see [AF21]) to the Yang-Baxter equation and iterating it. This section focuses on a general discussion which does not rely on any particular choice of a bijectivisation of the Yang-Baxter equation. In further sections, we consider the simplest, i.e. *independent*, bijectivisation in a subfamily of vertex models. This subfamily is still quite general and, in particular, includes *q*-TASEP and TASEP.

### Bijectivisation of summation identities

We begin by recalling the basic notion of a bijectivisation for a summation identity with finitely many terms, see [BP19, Section 2]. Let *A*, *B* be two disjoint finite sets. Also, introduce a positive weight function *w*(*x*) for each element $x \in A \cup B$ so that the following identity holds:

7.1 $$\begin{aligned} \sum _{a\in A}w(a) = \sum _{b\in B}w(b). \end{aligned}$$

In particular, identity (7.1) defines probability distributions on the sets *A* and *B* with probability weights proportional to $\{w(a)\}_{a\in A}$ and $\{w(b)\}_{b\in B}$, respectively. A bijectivisation is a coupling between these two probability distributions, expressed via conditional probabilities.

More precisely, a *bijectivisation* is a family of forward and backward transition probabilities $p^{\textrm{fwd}}(a \rightarrow b)\ge 0$, $p^{\textrm{bwd}}(b \rightarrow a)\ge 0$, for $a\in A$, $b\in B$, satisfying the following *stochasticity* and *detailed balance equation*:

7.2 $$\begin{aligned} \begin{aligned}&\sum _{b \in B} p^{\textrm{fwd}}(a \rightarrow b) = 1 \quad \forall a \in A, \qquad \qquad \sum _{a \in A} p^{\textrm{bwd}}(b \rightarrow a) = 1 \quad \forall b \in B. \\&\hspace{35pt} w(a)\, p^{\textrm{fwd}}(a \rightarrow b) = w(b)\, p^{\textrm{bwd}}(b \rightarrow a), \qquad \forall a\in A,\ b\in B. \end{aligned} \end{aligned}$$

For general sets *A* and *B*, a bijectivisation exists and it is not unique. However, in the special case when the cardinality of the sets *A* or *B*, i.e. |*A*| or |*B*|, is equal to 1, a bijectivisation is unique. For instance, when $|A|=1$ and $A=\left\{ a_0 \right\}$, we have

$$\begin{aligned} p^{\textrm{fwd}}(a_0 \rightarrow b)=\frac{w(b)}{w(a_0)}, \qquad p^{\textrm{bwd}}(b \rightarrow a_0)=1, \qquad \forall b\in B. \end{aligned}$$

In the case when $|A|=|B|=2$, the dimension of the space of all possible solutions to the linear equations (7.2) is equal to one, meaning that there is a one-parameter family of bijectivizations for this case.

### Bijectivisation of the vertical Yang-Baxter equation

Let us determine a bijectivisation to the vertical Yang-Baxter equation from Proposition 3.1. The equation depends on four parameters $u_1,u_2\ge 0$ and $s_1,s_2\in (-1,0]$. Recall that the path conservation implies that the sums in both sides of the Yang-Baxter equation (3.3) are actually finite. Additionally, all terms in the sums for the Yang-Baxter equation are nonnegative if $(u_2/u_1,s_1,s_2)\in \mathcal {R}$; see Definition 3.6. For fixed $i_1,j_1 \in \left\{ 0,1,\ldots ,J \right\}$, $i_2,i_3,j_2,j_3\in \mathbb {Z}_{\ge 0}$, we denote the terms on the left and right side of the Yang-Baxter equation, respectively, by the following weight functions:

7.3 $$\begin{aligned} \begin{aligned}&w_{i_1,j_1}^{\textrm{LHS}}(k_2,k_3\mid i_2,i_3;j_2,j_3)= R_{\frac{u_2}{u_1},s_1,s_2}(j_3,k_2;k_3,j_2) \hspace{1pt}L^{(J)}_{u_1,s_1}(i_2,i_1;k_2,k_1) \hspace{1pt}\\ &\quad L^{(J)}_{u_2,s_2}(i_3,k_1;k_3,j_1), \\ &w_{i_1,j_1}^{\textrm{RHS}}(k_3',k_2'\mid i_2,i_3;j_2,j_3)= L^{(J)}_{u_2,s_2}(k_3',i_1;j_3,k_1')\hspace{1pt}L^{(J)}_{u_1,s_1}(k_2',k_1';j_2,j_1)\hspace{1pt}\\ &\quad R_{\frac{u_2}{u_1},s_1,s_2}(k_3',i_2;i_3,k_2'), \end{aligned} \end{aligned}$$

where $k_1,k_1'$ are omitted in the notation of the weight functions since they may be determined through the path conservation:

$$\begin{aligned} k_1=i_1+i_2-k_2, \qquad k_1'=j_1+j_2-k_2'. \end{aligned}$$

Throughout the current Sect. 7, we denote any choice of transition probabilities coming from a bijectivisation of the Yang-Baxter equation (3.3) by

7.4 $$\begin{aligned} &  p^{\downarrow }_{i_1,j_1}[(k_2,k_3)\rightarrow (k_3',k_2')\mid i_2,i_3,j_2,j_3] \qquad \text {and}\qquad \nonumber \\  &  p^{\uparrow }_{i_1,j_1}[(k_3',k_2')\rightarrow (k_2,k_3)\mid i_2,i_3,j_2,j_3]. \end{aligned}$$

**Fig. 12:**
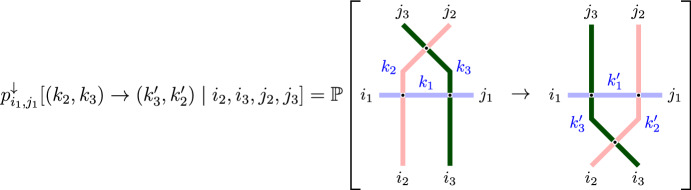
Graphical illustration of the down transition probability coming from a bijectivisation of the Yang-Baxter equation. The up transition is similar, with the cross vertex moving upwards instead

Here, the down and up arrows indicate the direction in which the cross vertex is moved. See Fig. 12 for an illustration.

#### Remark 7.1

*(Bijectivisation with infinitely many paths).* We tacitly included the case of infinitely many paths $i_2=j_3=+\infty$ (arising at the left boundary of the stochastic higher spin six vertex model, see Sect. 3.5) into the notation (7.4). In this case, the range of the down transition probability (i.e. the set of possible values of $(\infty ,k_2')$) is always finite. However, the range of the up transition probability

$$\begin{aligned} p^{\uparrow }_{*,j_1}[(\infty ,k_2')\rightarrow (\infty ,k_3)\mid \infty , i_3, j_2, \infty ] \end{aligned}$$

(“$*$” means that there is no dependence on $i_1$) may be infinite, since both $k_1$ and $k_3$ may be arbitrarily large, provided that $i_3+k_1=k_3+j_1$, because the stochastic higher spin six vertex model allows for an unbounded number of paths per horizontal edge by letting $q^J$ be an independent parameter with $J\notin \mathbb {Z}_{\ge 1}$.

Throughout the rest of this section, we assume that a well-defined bijectivisation at the left boundary exists. In considering explicit bijectivisations for the present paper, we restrict our attention to models with $J=1$ and, thus, the issue of an infinite range of the up transition probability does not arise.

In the current Sect. 7, we explain the general framework for a bijectivisation and couplings of measures on trajectories of stochastic vertex models. We do not pursue an explicit computation of possible transition probabilities $p^{\downarrow }_{i_1,j_1}$ and $p^{\uparrow }_{i_1,j_1}$ in the fully general case when all three vertex weights entering the Yang-Baxter equation have a *q*-hypergeometric form. Below in Sect. 8, we focus on a, still rather general, subfamily of vertex models for which the cross vertex weights factorize and become *q*-beta-binomial as in Sect. 3.4. Moreover, we set $J=1$ in the weights $L^{(J)}_{u_i,s_i}$, which forces $i_1,j_1$ to be either zero or one. For this subfamily, an explicit treatment of a bijectivisation is accessible.

### Down and up transitions on vertex model configurations

Recall the space $\mathscr {G}$ whose elements encode vertical paths crossing a given horizontal slice in the stochastic higher spin six vertex model, see Sect. 2.3. Recall the transition operator of the stochastic vertex model $T_{\textbf{u},\textbf{s}}$ (Sect. 2.3) and the swap operator $P^{(n)}_{z,s_1,s_2}$ ((Sect. 4.1). These operators satisfy a (quasi-)computation relation (Proposition 4.2) which follows from the Yang-Baxter equation. Here we employ bijectivisation of this intertwining relation to define up and down transitions on vertex model configurations.

Fix $n\in \mathbb {Z}_{\ge 1}$ and abbreviate throughout the rest of this section:

7.5 $$\begin{aligned} P^{(n)}=P^{(n)}_{u_n/u_{n-1},s_{n-1},s_n}, \qquad T=T_{\textbf{u},\textbf{s}}, \qquad T_{\sigma }= T_{\sigma \textbf{u},\sigma \textbf{s}}, \end{aligned}$$

where $\sigma$ is an arbitrary permutation from the infinite symmetric group (that is, $\sigma$ acts on $\mathbb {Z}_{\ge 0}$ by fixing all but finitely many points). The intertwining relation from Proposition 4.2 is

**Fig. 13:**
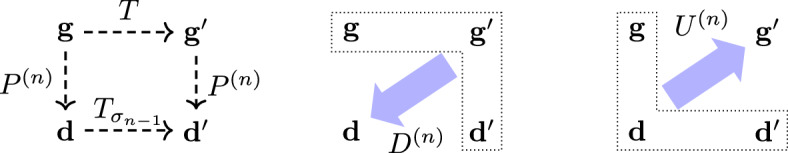
Left: A commuting diagram of Markov operators, where $\textbf{g},\textbf{g}',\textbf{d},\textbf{d}'\in \mathscr {G}$ with the notation from (7.5). The element $\textbf{g}$ is fixed, and all other elements are random. Intertwining means that the distributions of $\textbf{d}'$ obtained along both paths (right-down and down-right) coincide. Center and right: Markov operators $D^{(n)}$ and $U^{(n)}$ constructed from bijectivisation

7.6 $$\begin{aligned} TP^{(n)}=P^{(n)}T_{\sigma _{n-1}}, \end{aligned}$$

see Fig. 13, left, for an illustration. Here $\sigma _{n-1}=(n-1,n)$ is an elementary transposition. Recall that we are writing products of Markov operators as acting on measures, cf. Remark 4.3.

Relation (7.6) follows from a single Yang-Baxter equation illustrated in Fig. 10. Note that all the terms of this Yang-Baxter equation are nonnegative if we assume that the parameters $\textbf{u},\textbf{s}$ of the operators (7.5) satisfy the conditions of Proposition 4.2. Taking a bijectivisation (7.4) of this Yang-Baxter equation, we arrive at the following down and up Markov operators.

#### Definition 7.2

*(Down Markov operators for swaps).* Fix $n\in \mathbb {Z}_{\ge 1}$ and $\textbf{g},\textbf{g}',\textbf{d}'\in \mathscr {G}$ such that $T(\textbf{g},\textbf{g}')\hspace{1pt}P^{(n)}(\textbf{g}',\textbf{d}')\ne 0$. Define a random element $\textbf{d}\in \mathscr {G}$ such that $d_l=g_l$ for all $l\ne n-1,n$, and $d_{n-1},d_n$ are random and chosen from the distribution

$$\begin{aligned} p^{\downarrow }_{i_1,j_1}[(g_{n-1}',g_n')\rightarrow (d_{n-1},d_n)\mid g_{n-1},g_n,d_{n-1}',d_n'], \end{aligned}$$

with $i_1,j_1$ given by the numbers of horizontal paths determined from the configurations of vertical paths

7.7 $$\begin{aligned} \textstyle i_1 = \sum _{l \ge n-1}d_l' - \sum _{l \ge n-1}g_l, \qquad j_1 = \sum _{l \ge n+1}d_l' - \sum _{l \ge n+1}g_l. \end{aligned}$$

Let $D^{(n)}(\textbf{g}'\rightarrow \textbf{d}\mid \textbf{g},\textbf{d}')$ denote the probability weight of $\textbf{d}$, and call $D^{(n)}= D^{(n)}_{u_{n-1},s_{n-1};u_n,s_n}$ the *down Markov operator* corresponding to the swap operator $P^{(n)}$ at sites $n-1,n$. See Fig. 13, center, for an illustration.

#### Definition 7.3

*(Up Markov operators for swaps).* Fix $n\in \mathbb {Z}_{\ge 1}$ and $\textbf{g},\textbf{d},\textbf{d}'\in \mathscr {G}$ such that $P^{(n)}(\textbf{g},\textbf{d}) \hspace{1pt}T_{\sigma _{n-1}}(\textbf{d},\textbf{d}')\ne 0$. Define a random element $\textbf{g}'\in \mathscr {G}$ such that $g'_l=d'_l$ for all $l\ne n-1,n$, and $g'_{n-1},g'_n$ are random and chosen from the distribution

$$\begin{aligned} p^{\uparrow }_{i_1,j_1} [(d_{n-1},d_n)\rightarrow (g'_{n-1},g'_n)\mid g_{n-1},g_n,d_{n-1}',d_n'], \end{aligned}$$

with $i_1,j_1$ given by (7.7). Let $U^{(n)}(\textbf{d}\rightarrow \textbf{g}'\mid \textbf{g},\textbf{d}')$ denote the probability weight of $\textbf{g}'$, and call $U^{(n)}=U^{(n)}_{u_{n-1},s_{n-1};u_n,s_n}$ the *up Markov operator* corresponding to $P^{(n)}$. See Fig. 13, right, for an illustration.

The operators $D^{(n)}, U^{(n)}$ depend on the parameters $u_{n-1},s_{n-1},u_n,s_n$ (which we often omit from the notation) and on the choice of bijectivisation which typically is not unique. For any choice of bijectivisation, the down and up operators satisfy the stochasticity

$$\begin{aligned} \sum _{\textbf{d}\in \mathscr {G}}D^{(n)}(\textbf{g}'\rightarrow \textbf{d}\mid \textbf{g},\textbf{d}')=1 \quad \forall \textbf{g},\textbf{g}',\mathbf {d'}; \qquad \sum _{\textbf{g}'\in \mathscr {G}}U^{(n)}(\textbf{d}\rightarrow \textbf{g}'\mid \textbf{g},\textbf{d}')=1 \quad \forall \textbf{g},\textbf{d},\mathbf {d'}, \end{aligned}$$

and the detailed balance equation

7.8 $$\begin{aligned} T(\textbf{g},\textbf{g}') \hspace{1pt}P^{(n)}(\textbf{g}',\textbf{d}') \hspace{1pt}D^{(n)}(\textbf{g}'\rightarrow \textbf{d}\mid \textbf{g},\textbf{d}') {=} P^{(n)}(\textbf{g},\textbf{d}) \hspace{1pt}T_{\sigma _{n-1}}(\textbf{d},\textbf{d}') \hspace{1pt}U^{(n)}(\textbf{d}\rightarrow \textbf{g}'\mid \textbf{g},\textbf{d}')\nonumber \\ \end{aligned}$$

for any quadruple $\textbf{g},\textbf{g}',\textbf{d},\textbf{d}'\in \mathscr {G}$. Note that when, say, $T(\textbf{g},\textbf{g}') \hspace{1pt}P^{(n)}(\textbf{g}',\textbf{d}')=0$ (in contradiction with the assumption in Definition 7.2), the value of $D^{(n)}$ is irrelevant in (7.8) and, additionally, the corresponding value of $U^{(n)}$ must be zero to satisfy the detailed balance. Moreover, observe that summing (7.8) over $\textbf{d}$ and $\textbf{g}'$ results in the intertwining relation (7.6).

### Down and up transitions related to the Markov shift operator

Throughout the rest of this section we continue to use abbreviations (7.5), and also introduce the following abbreviations

**Fig. 14:**
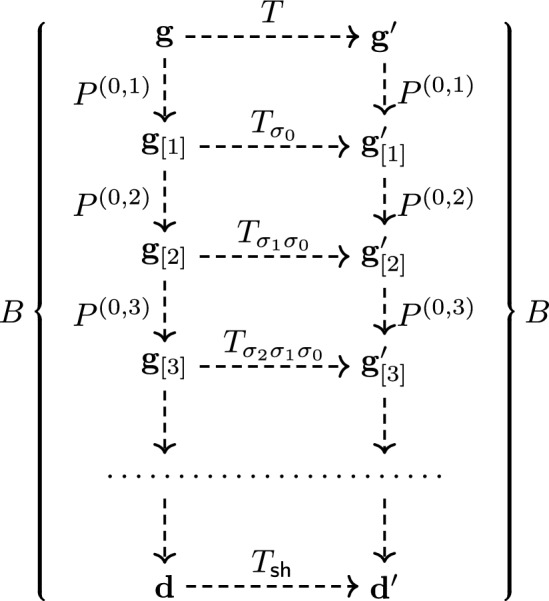
Relation $TB=BT_{\textsf{sh}}$ is a consequence of the tower of intertwining relations displayed in the figure. The random configuration $\textbf{d}\in \mathscr {G}$ distributed according to $D^{\bullet }(\textbf{g}'\rightarrow \textbf{d}\mid \textbf{g},\textbf{d}')$ is constructed as follows. Fix $\textbf{g},\textbf{g}',\textbf{d}'\in \mathscr {G}$. This completely determines $\textbf{g}'_{[m]}$ for all $m\ge 1$, see (7.13)–(7.12). Apply the down Markov operator for swaps to each square of the tower from the top of the diagram to the bottom; see Definition 7.2. First, use $D^{(0,1)}$ to sample $\textbf{g}_{[1]}$ given $\textbf{g},\textbf{g}',\textbf{g}'_{[1]}$, and continue consecutively using $D^{(0,m)}$ to sample to sample $\textbf{g}_{[m]}$ given $\textbf{g}_{[m-1]},\textbf{g}'_{[m]},\textbf{g}'_{[m+1]}$ for $m\ge 2$. Once the update terminates, we get the desired random element $\textbf{d}\in \mathscr {G}$. The fact that the sequence terminates is due to Lemma 7.4. The up operator $U^{\bullet }$ is defined similarly by applying the up Markov operator for swaps to each square of the tower from bottom of the diagram to the top; see Definition 7.3

7.9 $$\begin{aligned} P^{(0,n)}= P^{(n)}_{u_n/u_{0},s_{0},s_n}, \qquad B=B_{\textbf{u},\textbf{s}}, \qquad T_{\textsf{sh}}=T_{\textsf{sh}(\textbf{u}),\textsf{sh}(\textbf{s})}, \end{aligned}$$

where $\textsf{sh}$ is the shift (4.4). Recall that the Markov shift operator *B* is obtained by iterating the swap operators $P^{(0,n)}$ over all $n\in \mathbb {Z}_{\ge 1}$, see (4.6). Iterating the down or up operators in a similar manner would result in Markov operators on $\mathscr {G}$ denoted by $D^{\bullet }$ and $U^{\bullet }$ which satisfy the following detailed balance equation:

7.10 $$\begin{aligned} T(\textbf{g},\textbf{g}') \hspace{1pt}B(\textbf{g}',\textbf{d}') \hspace{1pt}D^{\bullet }(\textbf{g}'\rightarrow \textbf{d}\mid \textbf{g},\textbf{d}') = B(\textbf{g},\textbf{d}) \hspace{1pt}T_{\textsf{sh}}(\textbf{d},\textbf{d}') \hspace{1pt}U^{\bullet }(\textbf{d}\rightarrow \textbf{g}'\mid \textbf{g},\textbf{d}') \nonumber \\ \end{aligned}$$

for any $\textbf{g},\textbf{g}',\textbf{d},\textbf{d}'\in \mathscr {G}$. Graphically, one can extract the definition of $D^{\bullet }$ and $U^{\bullet }$ from the tower of intertwining relations in Fig. 14. However, to describe these operators in full detail we need some notation and observations.

Denote

7.11 $$\begin{aligned} D^{(0,n)}=D^{(n)}_{u_0,s_0;u_n,s_n}, \qquad U^{(0,n)}=U^{(n)}_{u_0,s_0;u_n,s_n}. \end{aligned}$$

That is, $D^{(0,n)}$ randomly changes $(g_{n-1}',g_n')$ to $(d_{n-1},d_n)$, but uses the parameters $(u_0,s_0)$ and $(u_n,s_n)$ instead of the ones in Definition 7.2, and similarly for $U^{(0,n)}$. Let the parameters of the vertex model satisfy $(\textbf{u},\textbf{s})\in \mathcal {T}\cap \mathcal {B}$, so that the operators $T,T_{\textsf{sh}}$, and *B* are well-defined (see (2.17)–(2.18) and Definition 4.4). Moreover, assume that the up transition $U^{(0,1)}$ at the left boundary is also well-defined, cf. Remark 7.1.

Now, let us encode the path configurations at intermediate horizontal slices in the vertex model for $B(\textbf{g},\textbf{d})$ given in Fig. 11. For $\textbf{g},\textbf{d}\in \mathscr {G}$ with $B(\textbf{g},\textbf{d})\ne 0$, we denote the $m^{th}$ horizontal slice by $\textbf{g}_{[m]}$ and it is given by

7.12 $$\begin{aligned} (\textbf{g}_{[m]})_l={\left\{ \begin{array}{ll} d_l,& l<m;\\ h_m,& l=m;\\ g_l,& l>m; \end{array}\right. } \end{aligned}$$

for $m\in \mathbb {Z}_{\ge 0}$ so that the number of vertical arrows at the $m^{th}$ position is given by

7.13 $$\begin{aligned} h_m:=\sum _{l\ge m}d_l-\sum _{l\ge m+1}g_l,\qquad m\in \mathbb {Z}_{\ge 1}. \end{aligned}$$

Note that $h_m=0$ for all sufficiently large *m* since $\textbf{d},\textbf{g}\in \mathscr {G}$. Also, note that $\textbf{g}_{[0]}$ coincides with $\textbf{g}$.

#### Lemma 7.4

Let $\textbf{g},\textbf{d}\in \mathscr {G}$ and $M>1+\max \left\{ l\in \mathbb {Z}_{\ge 1}:g_l>0 \ \text {or}\ d_l>0 \right\}$. Then

7.14 $$\begin{aligned} B(\textbf{g},\textbf{d}) = \prod _{m=1}^{M} P^{(0,m)}(\textbf{g}_{[m-1]},\textbf{g}_{[m]}) \end{aligned}$$

and $$\textbf{d}=\textbf{g}_{[M]}$$.

Lemma 4.6 essentially shows that for fixed $\textbf{g}$, the sum of (7.14) over all $\textbf{d}$ is equal to 1. Since *M* depends on $\textbf{d}$ in (7.14), Lemma 7.4 does not imply Lemma 4.6.

#### Proof of Lemma 7.4

For all $m\ge M$, we have $h_m=0$ by the lower bound on *M*. Hence, $\textbf{g}_{[m-1]}=\textbf{g}_{[m]}$ for $m\ge M$. In particular, for $m\ge M$, we have $P^{(0,m)}(\textbf{g}_{[m-1]},\textbf{g}_{[m]}) = \textbf{1}_{\textbf{g}_{[m]}=\textbf{g}_{[m-1]}}$ since the path configuration, as in Fig. 11, is empty to the right of location *M*. Thus, we may truncate the infinite product of swap operators $P^{(0,m)}$ that define the operator *B*, see (4.6), to the product of the swap operators $P^{(0,m)}$ in (7.14). Additionally, note that the $\textbf{g}_{[m]}$’s stabilize to $\textbf{d}$. This completes the proof.

For $\textbf{g}',\textbf{d}'$ with $B(\textbf{g}',\textbf{d}')\ne 0$ and $m\in \mathbb {Z}_{\ge 0}$, define $\textbf{g}'_{[m]}$ in the same way as in (7.13)–(7.12). The construction of the tower of intertwining relations given in Fig. 14 follows from the representation (7.14) of the shift operators together with (7.6). Note, in particular, that the tower is finite since the $\textbf{g}_{[m]}$ and $\textbf{g}'_{[m]}$ stabilize as is shown below.

#### Lemma 7.5

Let $\textbf{g},\textbf{g}',\textbf{d},\textbf{d}'\in \mathscr {G}$, and $M>1+\max \left\{ l\in \mathbb {Z}_{\ge 1}:\max (g_l,g_l',d_l,d_l')>0 \right\}$. Then, for any $m\ge M$, we have

7.15 $$\begin{aligned} T_{\sigma _{m-2}\sigma _{m-3}\ldots \sigma _1\sigma _0 } (\textbf{g}_{[m-1]},\textbf{g}'_{[m-1]})= T_{\sigma _{m-1}\sigma _{m-2}\sigma _{m-3}\ldots \sigma _1\sigma _0 } (\textbf{g}_{[m]},\textbf{g}'_{[m]}), \end{aligned}$$

and

7.16 $$\begin{aligned} \begin{aligned} D^{(0,m)}(\textbf{g}'_{[m-1]}\rightarrow \textbf{g}_{[m]}\mid \textbf{g}_{[m-1]},\textbf{g}_{[m]}')&= \textbf{1}_{\textbf{g}_{[m]}=\textbf{g}_{[m-1]}}, \\ U^{(0,m)}(\textbf{g}_{[m]}\rightarrow \textbf{g}'_{[m-1]}\mid \textbf{g}_{[m-1]},\textbf{g}_{[m]}')&= \textbf{1}_{\textbf{g}'_{[m-1]}=\textbf{g}_{[m]}'}. \end{aligned} \end{aligned}$$

#### Proof

Observe that the transfer matrices $T_{\sigma _{m-2}\ldots \sigma _1\sigma _0 }$ and $T_{\sigma _{m-1}\sigma _{m-2}\ldots \sigma _1\sigma _0 }$ differ only by the location of the parameter $s_0$. Moreover, for $m\ge M$, the action of the these transfer matrices on the configuration $\textbf{g}_{[m]}$ does not depend on $s_0$ since the configuration is empty to the right of *M*. Therefore, the action is the same. This proves (7.15).

Next, notice that $P^{(0,m)}$ acts as identity on our elements for $m\ge M$, see the proof of Lemma 7.4. Then, along with (7.15), this implies that the detailed balance equation for $D^{(0,m)},U^{(0,m)}$ has a unique solution given by (7.16). This completes the proof. $\square$

#### Definition 7.6

[*Down operator for shift*]. Let $\textbf{g},\textbf{g}',\textbf{d}'\in \mathscr {G}$ be such that $T(\textbf{g},\textbf{g}')B(\textbf{g}',\textbf{d}') \ne 0$. The *down Markov operator* corresponding to the shift operator *B* is defined as follows:

7.17 $$\begin{aligned} D^{\bullet }(\textbf{g}'\rightarrow \textbf{d}\mid \textbf{g},\textbf{d}') :=\prod _{m=1}^{\infty } D^{(0,m)}(\textbf{g}'_{[m-1]}\rightarrow \textbf{g}_{[m]}\mid \textbf{g}_{[m-1]},\textbf{g}_{[m]}'), \qquad \textbf{d}=\lim _{m\rightarrow +\infty }\textbf{g}_{[m]}\in \mathscr {G}.\nonumber \\ \end{aligned}$$

Due to Lemma 7.5, the product is actually finite and the limit stabilizes. See Figs. 13 and 14 for an illustration

Note that for any $\textbf{g},\textbf{g}',\textbf{d}'\in \mathscr {G}$, there are only finitely many $\textbf{d}\in \mathscr {G}$ for which (7.17) is nonzero. This is due to the fact that there are only finitely many $\textbf{d}$ for which $T_{\textsf{sh}}(\textbf{d},\textbf{d}')\ne 0$, see the desired detailed balance equation (7.10).

#### Definition 7.7

[*Up operator for shift*]. Let $\textbf{g},\textbf{d},\textbf{d}'\in \mathscr {G}$ be such that $B(\textbf{g},\textbf{d}) T_\textsf{sh}(\textbf{d},\textbf{d}')\ne 0$. The *up Markov operator* corresponding to the shift operator *B* is defined as follows:

7.18 $$\begin{aligned} U^{\bullet }(\textbf{d}\rightarrow \textbf{g}'\mid \textbf{g},\textbf{d}') :=\prod _{m=1}^{\infty } U^{(0,m)}(\textbf{g}_{[m]}\rightarrow \textbf{g}'_{[m-1]}\mid \textbf{g}_{[m-1]},\textbf{g}_{[m]}'), \qquad \textbf{g}'=\textbf{g}'_{[0]}\in \mathscr {G}.\nonumber \\ \end{aligned}$$

Due to Lemma 7.5, the product is actually finite. See Figs. 13 and 14 for an illustration.

We assume that the bijectivisation at the left boundary is well-defined, so that the whole up operator $U^{\bullet }$ is also well-defined. In contrast with the down operator $D^{\bullet }$, in (7.18), the number of possible outcomes $\textbf{g}'$ for any fixed $\textbf{g},\textbf{d},\textbf{d}'$ may be infinite. These infinitely many choices arise at the left boundary, for $m=1$, as explained in Remark 7.1.

One readily sees that the operators $D^{\bullet }$ and $U^{\bullet }$ from Definitions 7.6 and 7.7 satisfy the detailed balance equation (7.10) involving the shift operator *B* and the stochastic higher spin six vertex model transfer matrices *T* and $T_{\textsf{sh}}$. This follows directly from the detailed balance equations for the Markov swap operators $D^{(0,n)}$ and $U^{(0,n)}$ given by (7.11).

#### Remark 7.8

Here, and in Sect. 7.3, we argued in terms of the space $\mathscr {G}$ of vertex model configurations. Note that we may define corresponding down and up Markov operators to act on the space $\mathscr {X}$ of exclusion process configurations via the gap-particle transformation (Definition 2.4). Following our convention so far, we use the notation $\tilde{D}^{(n)},{\tilde{D}}^{(0,n)},{\tilde{D}}^{\bullet }$, and so on, to denote these operators acting on $\mathscr {X}$.

### Coupling of measures on trajectories via rewriting history

We couple together trajectories of two instances of the stochastic higher spin six vertex model with different parameters through the use of the down and up Markov operators defined in Sects. 7.3 and 7.4. Here, we only consider this construction for the swap operator $P^{(n)}$ and the operators $D^{(n)},U^{(n)}$ from Sect. 7.3. The couplings involving the shift operator *B* work very similarly, and they will be discussed in a continuous time limit in Sect. 10 below.

Fix $n\in \mathbb {Z}_{\ge 1}$ and take parameters $(\textbf{u},\textbf{s})\in \mathcal {T}$ such that $\bigl ( \frac{u_n}{u_{n-1}},s_{n-1},s_n \bigr )\in \mathcal {R}$. That is, we assume that the parameters satisfy the conditions of Proposition 4.2, so that the Markov operators $T,T_{\sigma _{n-1}},P^{(n)}$ (7.5) are well-defined. Let $D^{(n)}$ and $U^{(n)}$ be the operators from Sect. 7.3 providing a bijectivisation of the intertwining relation $TP^{(n)}=P^{(n)}T_{\sigma _{n-1}}$ from Proposition 4.2.

Fix $M\ge 1$ and an initial configuration $\hat{\textbf{g}} \in \mathscr {G}$. Denote by $\{\textbf{g}(t)\}_{0\le t\le M}$ the stochastic higher spin six vertex model with parameters $(\textbf{u},\textbf{s})$ started from $\hat{\textbf{g}}$. Also, denote by $\{\textbf{d}(t)\}_{0\le t\le M}$ the vertex model with parameters $(\sigma _{n-1}\textbf{u},\sigma _{n-1}\textbf{s})$ started from a random initial configuration $\delta _{\hat{\textbf{g}}}P^{(n)}$. Let $\mathfrak {T}$ and $\mathfrak {T}^{\sigma _{n-1}}$, respectively, denote the measures on trajectories of these processes on $\mathscr {G}$. Then, in particular, the probability weights for these measures are given by

7.19 $$\begin{aligned} \begin{aligned}&\mathfrak {T} (\textbf{g}(0),\textbf{g}(1),\ldots ,\textbf{g}(M) )= \textbf{1}_{\textbf{g}(0)=\hat{\textbf{g}}} \hspace{1pt}T(\textbf{g}(0),\textbf{g}(1))\hspace{1pt}T(\textbf{g}(1),\textbf{g}(2)) \ldots T(\textbf{g}(M-1),\textbf{g}(M)),\\&\mathfrak {T}^{\sigma _{n-1}} (\textbf{d}(0),\textbf{d}(1),\ldots ,\textbf{d}(M) ) = P^{(n)}(\hat{\textbf{g}},\textbf{d}(0)) \hspace{1pt}T_{\sigma _{n-1}}(\textbf{d}(0),\textbf{d}(1)) \ldots T_{\sigma _{n-1}}(\textbf{d}(M-1),\textbf{d}(M)). \end{aligned}\nonumber \\ \end{aligned}$$

**Fig. 15:**
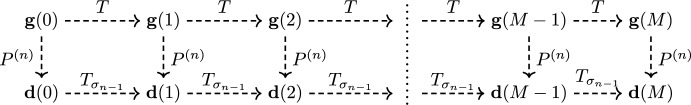
The chain of intertwining relations providing the coupling of trajectories (Theorem 7.9) of the two processes in (7.19)

The iterated intertwining relation $T^M P^{(n)}=P^{(n)}(T_{\sigma _{n-1}})^{M}$ implies that the distribution of the final state $\textbf{d}(M)$ of $\mathfrak {T}^{\sigma _{n-1}}$ is the same as the distribution of $\delta _{\textbf{g}(M)}P^{(n)}$, obtained by applying $P^{(n)}$ to the final state of $\mathfrak {T}$ (see Fig. 15 for an illustration). The next statement extends this identity in distribution to couplings between *joint distributions in time*; this is the main result of the current Sect. 7. These couplings have a sequential nature (where the time *t* runs through $t\in \left\{ 0,1,\ldots ,M \right\}$), and may be thought of as “rewriting the history” of a vertex model. There are two distinct sequential couplings corresponding to the direction in which the time *t* is varied.

#### Theorem 7.9

**1.**: (Rewriting history from future to past) Fix $\hat{\textbf{g}}\in \mathscr {G}$. Let $\{\textbf{g}(t)\}_{0\le t\le M}$ be distributed according to $\mathfrak {T}$. First, apply $P^{(n)}$ to $\textbf{g}(M)$, and denote this random configuration by $\textbf{d}'(M)$. Sequentially in the order $t=M-1,M-2,\ldots ,1,0$, let $\textbf{d}'(t)$ be sampled from
  7.20 $$\begin{aligned} D^{(n)}(\textbf{g}(t+1)\rightarrow \textbf{d}'(t)\mid \textbf{g}(t),\textbf{d}'(t+1)). \end{aligned}$$
  Then, the joint distribution of $\{\textbf{d}'(t)\}_{0\le t\le M}$ is equal to $\mathfrak {T}^{\sigma _{n-1}}$.
**2.**: (Rewriting history from past to future) Fix $\hat{\textbf{g}}\in \mathscr {G}$. Let $\{\textbf{d}(t)\}_{0\le t\le M}$ be distributed according to $\mathfrak {T}^{\sigma _{n-1}}$, where now the initial condition is random and depends on $\hat{\textbf{g}}$. Sequentially in the order $t=1,2,\ldots ,M$, let $\textbf{g}'(t)$ be sampled from
  7.21 $$\begin{aligned} U^{(n)}(\textbf{d}(t-1)\rightarrow \textbf{g}'(t)\mid \textbf{g}'(t-1),\textbf{d}(t)), \end{aligned}$$
  where, by agreement, $\textbf{g}'(0)=\hat{\textbf{g}}$. Then, the joint distribution of $\{\textbf{g}'(t)\}_{0\le t\le M}$ is equal to $\mathfrak {T}$.

#### Proof

The results follow by iterating the detailed balance equation (7.8) involving $T,T_{\sigma _{n-1}}$, and $P^{(n)}$. Moreover, by the Markov property, it suffices to consider joint distributions at adjacent time moments $t,t+1$, and use induction in *t*. This induction is descending or ascending in the first or the second part, respectively. See Fig. 15 for an illustration of the notation employed throughout the proof. We give more details below.

Consider the first part. Inductively, we show that the transition probability $\textbf{d}'(t) \rightarrow \textbf{d}'(t+1)$ is equal to $T_{\sigma _{n-1}} (\textbf{d}'(t), \textbf{d}'(t+1))$ if the transition probability for $\textbf{g}(t) \rightarrow \textbf{g}(t+1)$ is equal to $T( \textbf{g}(t), \textbf{g}(t+1))$. Additionally, for the induction argument, we show that the transition probability for $\textbf{g}(t) \rightarrow \textbf{d}'(t)$ is equal to $P^{(n)}(\textbf{g}(t), \textbf{d}'(t))$ if the transition probability for $\textbf{g}(t+1) \rightarrow \textbf{d}'(t+1)$ is equal to $P^{(n)}(\textbf{g}(t+1), \textbf{d}'(t+1))$. We start the induction by noting that the conditions are true for the first step when $t+1 = M$ by assumption. In the following, we carry out the computations for the induction step.

Let us assume that $\textbf{g}(t)$ is known. That is, the following computations are conditioned on $\textbf{g}(t)$. Then, we have the following chain of expressions for the joint distribution of $\textbf{d}'(t),\textbf{d}'(t+1)$ conditioned on $\textbf{g}(t)$:

$$\begin{aligned}&{\mathbb {P}}\left( \textbf{d}'(t+1),\textbf{d}'(t) \mid \textbf{g}(t) \right) \\&= {\mathbb {P}}\left( \textbf{d}'(t) \mid \textbf{g}(t)\right) \hspace{1pt}{\mathbb {P}}\left( \textbf{d}'(t+1) \mid \textbf{d}'(t), \textbf{g}(t) \right) \\&= \sum _{\textbf{g}(t+1)} {\mathbb {P}}(\textbf{g}(t+1), \textbf{d}'(t+1),\textbf{d}'(t) \mid \textbf{g}(t) )\\&=\sum _{\textbf{g}(t+1)} {\mathbb {P}}( \textbf{g}(t+1) \mid \textbf{g}(t))\hspace{1pt}{\mathbb {P}}( \textbf{d}'(t+1) \mid \textbf{g}(t+1), \textbf{g}(t))\hspace{1pt}{\mathbb {P}}( \textbf{d}'(t) \mid \textbf{g}(t+1), \textbf{g}(t), \textbf{d}'(t+1)) \\&=\sum _{\textbf{g}(t+1)}T(\textbf{g}(t),\textbf{g}(t+1)) \hspace{1pt}P^{(n)}(\textbf{g}(t+1),\textbf{d}'(t+1)) \hspace{1pt}D^{(n)}(\textbf{g}(t+1)\rightarrow \textbf{d}'(t)\mid \textbf{g}(t),\textbf{d}'(t+1))\\&=\sum _{\textbf{g}(t+1)}P^{(n)}(\textbf{g}(t),\textbf{d}'(t)) \hspace{1pt}T_{\sigma _{n-1}}(\textbf{d}'(t),\textbf{d}'(t+1)) U^{(n)}(\textbf{d}'(t) \rightarrow \textbf{g}(t+1)\mid \textbf{g}(t),\textbf{d}'(t+1))\\&=P^{(n)}(\textbf{g}(t),\textbf{d}'(t)) \hspace{1pt}T_{\sigma _{n-1}}(\textbf{d}'(t),\textbf{d}'(t+1)). \end{aligned}$$

We used the induction hypothesis on the fourth equality, detailed balance equation for the fifth equality, and the stochasticity for the sixth equality. From the identity above we see that the conditional distribution of $\textbf{d}'(t+1)$ given $\textbf{d}'(t)$ is $T_{\sigma _{n-1}}(\textbf{d}'(t),\textbf{d}'(t+1))$, which is independent of $\textbf{g}(t)$. Moreover, the marginal distribution of $\textbf{d}'(t)$ is $P^{(n)}(\textbf{g}(t),\textbf{d}'(t))$, which allows to continue the induction. Thus, the result for the first part follows.

The second part is proven similarly, with a simplification that we do not need to condition the computations on $\textbf{d}(t)$ due to the other direction of the Markov step $P^{(n)}$. This completes the proof. $\square$

#### Definition 7.10

*(Markov operators for rewriting history).* Fix a trajectory $\{\textbf{g}(t)\}_{0\le t\le M}$ of the stochastic higher spin six vertex model with some initial data $\hat{\textbf{g}}$, and also fix an *arbitrary* configuration $\textbf{d}'(M)$ at the final time such that $P^{(n)}(\textbf{g}(M),\textbf{d}'(M))\ne 0$. Given $\textbf{d}'(M)$, denote by $H_n^{\leftarrow }$ the Markov operator that maps the trajectory $\{\textbf{g}(t)\}_{0\le t\le M}$ to the trajectory $\{\textbf{d}'(t)\}_{0\le t\le M}$ by the sequential application of $D^{(n)}$ as in the first part of Theorem 7.9. The operator $H_n^{\leftarrow }$ may be viewed as a Markov process with initial condition $\textbf{d}'(M)$ and running *backwards in time*, from future to past.

Similarly, fix a trajectory $\{\textbf{d}(t)\}_{0\le t\le M}$ with some initial data $\hat{\textbf{d}}$, and fix an *arbitrary* configuration $\hat{\textbf{g}}$ such that $P^{(n)}(\hat{\textbf{g}},\hat{\textbf{d}})\ne 0$. Given $\hat{\textbf{g}}$, denote by $H^{\rightarrow }_{n}$ the Markov operator that maps the trajectory $\{\textbf{d}(t)\}_{0\le t\le M}$ to the trajectory $\{\textbf{g}'(t)\}_{0\le t\le M}$ by the sequential application of $U^{(n)}$ as in the second part of Theorem 7.9. The operator $H^{\rightarrow }_{n}$ may be viewed as a Markov process with initial condition $\hat{\textbf{g}}$ and running *forward in time*, from past to future.

We call $H_n^{\leftarrow }$ and $H_n^{\rightarrow }$ the *Markov operators for rewriting history* corresponding to the swap operator $P^{(n)}$.

Note that both $H_n^{\leftarrow }$ and $H^{\rightarrow }_n$ act locally and change only the components $g_{n-1},g_n$ along the trajectory of the stochastic history of the spin six vertex model. This locality comes from the same feature of the Markov swap operator $P^{(n)}$.

We reformulate Theorem 7.9, with this definition. Recall that measures on trajectories are defined by (7.19).

#### Corollary 7.11

**1.**: If a trajectory $\{\textbf{g}(t)\}_{0\le t\le M}$ has distribution $\mathfrak {T}$ and $\textbf{d}'(M)$ has distribution $\delta _{\textbf{g}(M)}P^{(n)}$, then the application of $H_n^{\leftarrow }$ (with initial condition $\textbf{d}'(M)$) to $\{\textbf{g}(t)\}_{0\le t\le M}$ produces a trajectory with distribution $\mathfrak {T}^{\sigma _{n-1}}$.
**2.**: If a trajectory $\{\textbf{d}(t)\}_{0\le t\le M}$ has distribution $\mathfrak {T}^{\sigma _{n-1}}$ (in particular, its initial condition $\hat{\textbf{d}}$ has distribution $\delta _{\hat{\textbf{g}}}P^{(n)}$, where $\hat{\textbf{g}}$ is fixed), then the application of $H^{\rightarrow }_{n}$ (with initial condition $\hat{\textbf{g}}$) to $\{\textbf{d}(t)\}_{0\le t\le M}$ produces a trajectory with distributed $\mathfrak {T}$.

## Application to Discrete-time Particle Systems

We now consider the simplest bijectivisation for a subfamily of stochastic higher spin six vertex models We call this the independent bijectivisation. The advantage of this subfamily is that the cross vertex weights factorize into the *q*-beta-binomial form. The subfamily of stochastic higher spin six vertex models is still quite general and, in particular, includes *q*-TASEP and TASEP. In the following, we also translate the Markov operators $H_n^{\leftarrow }$ and $H^{\rightarrow }_n$ for rewriting history in these vertex models into the language of particle systems.

### Notation and independent bijectivisation

**Fig. 16:**
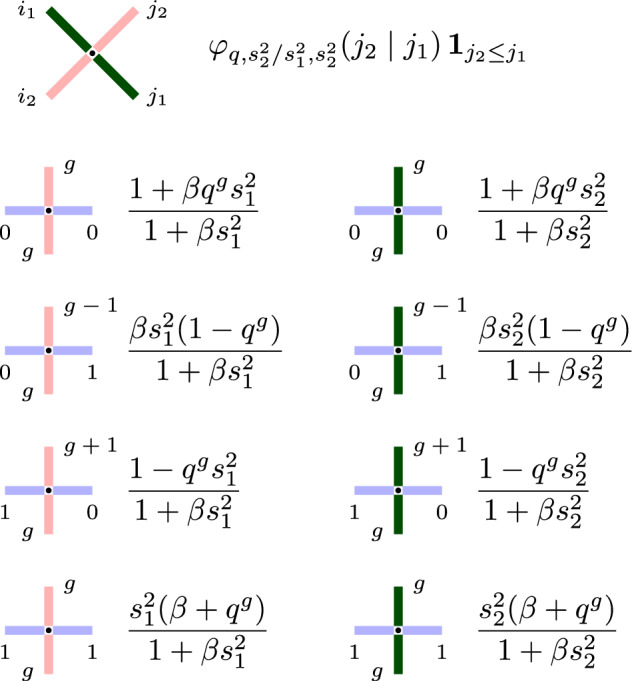
Vertex weights entering the Yang-Baxter equation considered in the current Sect. 8

Consider the setting of Sect. 7.2 (bijectivisation of a single Yang-Baxter equation) and take $J=1$, $u_1=-\beta s_1$, $u_2=-\beta s_2$, where $s_1,s_2\in (-1,0)$ with $|s_2|\le |s_1|$, and $\beta >0$. Then, the vertex weights in the Yang-Baxter equation (3.3) become as in Fig. 16. In particular, the cross vertex weights factorize into the *q*-beta-binomial form, see Proposition 3.5. Our conditions on the parameters make all the terms in the Yang-Baxter equation, i.e. the weights (7.3), nonnegative.

Recall that the boundary conditions for the Yang-Baxter equation are encoded by $i_1,j_1 \in \left\{ 0,1,\ldots ,J \right\}$ and $i_2,i_3,j_2,j_3\in \mathbb {Z}_{\ge 0}$, see Fig. 9. There are only four possible values for the pair $(i_1,j_1)\in \left\{ 0,1 \right\} ^2$ encoding the horizontal boundary conditions since we are taking $J=1$. Fixing $i_1,j_1$, let us employ the shorthand notation

8.1 $$\begin{aligned} i_2=a,\qquad i_3=b,\qquad j_2=c. \end{aligned}$$

We always have $0\le c\le b+1$, otherwise the cross vertex weight vanishes. The value of $j_3$ is recovered from the path conservation property:

8.2 $$\begin{aligned} j_3=a+b-c+i_1-j_1. \end{aligned}$$

Assuming that *a*, *b*, *c* are also fixed, the terms (7.3) in both sides of the Yang-Baxter equation, as well as the transition probabilities (7.4), may all be encoded by the numbers of paths through the internal horizontal edge $k_1,k_1'\in \left\{ 0,1 \right\}$, see Figs. 9 and 12. Indeed, given $k_1$, we may reconstruct $k_2,k_3$ from $a,b,c,i_1,j_1$, and similarly for $k_2',k_3'$ given $k_1'$. We use the following shorthand notation for the corresponding weights and transition probabilities:

8.3 $$\begin{aligned} w^{\textrm{LHS}}_{i_1,j_1}(k_1), \qquad w^{\textrm{RHS}}_{i_1,j_1}(k_1'), \qquad p^{\downarrow }_{i_1,j_1}[k_1\rightarrow k_1'], \qquad p^{\uparrow }_{i_1,j_1}[k_1'\rightarrow k_1]. \end{aligned}$$

The Yang-Baxter equation thus takes the form

8.4 $$\begin{aligned} w^{\textrm{LHS}}_{i_1,j_1}(0)+ w^{\textrm{LHS}}_{i_1,j_1}(1)= w^{\textrm{RHS}}_{i_1,j_1}(0)+ w^{\textrm{RHS}}_{i_1,j_1}(1). \end{aligned}$$

To simplify the constructions of our couplings, throughout the rest of the paper we consider the so-called *independent bijectivisation* so that the transition, say $k_1\rightarrow k_1'$, depends only on the end state $k_1'$ and the boundary conditions, and not on $k_1$. More specifically, we give the following definition:

#### Definition 8.1

For a fixed set of boundary parameters $a\in \mathbb {Z}_{\ge 0}\cup \left\{ +\infty \right\}$, $b,c\in \mathbb {Z}_{\ge 0}$ and $i_1,j_1\in \left\{ 0,1 \right\}$, the transition probabilities for the *independent bijectivisation* are given by:

8.5 $$\begin{aligned} p^{\downarrow }_{i_1,j_1}[k_1\rightarrow k_1'] :=\frac{w^{\textrm{RHS}}_{i_1,j_1}(k_1')}{w^{\textrm{RHS}}_{i_1,j_1}(0)+w^{\textrm{RHS}}_{i_1,j_1}(1)} \qquad p^{\uparrow }_{i_1,j_1}[k_1'\rightarrow k_1] :=\frac{w^{\textrm{LHS}}_{i_1,j_1}(k_1)}{w^{\textrm{LHS}}_{i_1,j_1}(0)+w^{\textrm{LHS}}_{i_1,j_1}(1)},\nonumber \\ \end{aligned}$$

with $$k_1,k_1'\in \left\{ 0,1 \right\} $$.

#### Remark 8.2

Note that the denominator is nonzero in each of the two expressions in (8.5). Otherwise, there is no Yang-Baxter equation with the given boundary conditions $a,b,c,i_1,j_1$ and, correspondingly, there is no bijectivisation. One readily sees that the transition probabilities in (8.5) are always nonnegative and satisfy the detailed balance equation (7.2). Observe that this bijectivisation corresponds to taking the coupling of measures on *A* and *B* described in Sect. 7.1 to be simply the product measure. For this reason we call (8.5) the *independent bijectivisation*.

The case $a=+\infty$ in (8.5) corresponds to having infinitely many paths through the leftmost vertical edges, but this does not present an issue since $J=1$; see Remark 7.1. In other words, the limits of $p^{\downarrow }_{i_1,j_1}[k_1\rightarrow k_1']$ and $p^{\uparrow }_{i_1,j_1}[k_1'\rightarrow k_1]$ as $a\rightarrow +\infty$ exist and give a well-defined bijectivisation of the Yang-Baxter equation with $i_2=j_3=+\infty$.

Formulas arising from (8.5) do not have factorized denominators and, in general, can have a rather complicated form despite the simplicity of the general definition. For example, we have

$$\begin{aligned} &  p^{\downarrow }_{0,0}[0\rightarrow 1] = p^{\downarrow }_{0,0}[1\rightarrow 1] \\= &  \quad \frac{ (1-q^{c-1}s_1^2)\beta s_2^2(1-q^{a+b-c+1})\hspace{1pt}\varphi (c-1\mid b) }{(1+q^c \beta s_1^2)(1+q^{a+b-c}\beta s_2^2)\hspace{1pt}\varphi (c\mid b) +(1-q^{c-1}s_1^2)\beta s_2^2(1-q^{a+b-c+1})\hspace{1pt}\varphi (c-1\mid b)}, \end{aligned}$$

where we abbreviated $\varphi =\varphi _{q,s_2^2/s_1^2,s_2^2}$. Note that this expression admits a straightforward limit as $a\rightarrow +\infty$, making the bijectivisation at the left edge well-defined.

### Rewriting history in particle systems from future to past

Let us now take the full stochastic higher spin six vertex model with $J=1$, $u_i=-\beta s_i$, $i\in \mathbb {Z}_{\ge 0}$, where the parameters of the model are

$$\begin{aligned} \beta >0,\qquad \textbf{s}=(s_0,s_1,s_2,\ldots ),\quad -1<s_i<0. \end{aligned}$$

This vertex model corresponds to a discrete time stochastic particle system $\{\textbf{x}(t)\}_{t\in \mathbb {Z}_{\ge 0}}$ on the space $\mathscr {X}$ of particle configurations on $\mathbb {Z}$, via the gap-particle transformation (Definition 2.4). For any $n\ge 1$, the bijectivisation defined in Sect. 8.1 gives rise to the Markov operators for rewriting history as in Definition 7.10. We denote the corresponding operators by ${\tilde{H}}_n^{\leftarrow }$ and $\tilde{H}_n^{\rightarrow }$, following the convention that operators on $\mathscr {X}$ include a tilde.

Fix $n\ge 1$ and assume that $|s_{n-1}|\ge |s_n|$. In this subsection, we describe the Markov operator $\tilde{H}_n^{\leftarrow }$ and, in the following Sect. 8.3, we describe the Markov operator ${\tilde{H}}_n^{\rightarrow }$. We first consider the down Markov operator ${\tilde{D}}^{(n)}$ from Definition 7.2. For a fixed time $0\le t\le M-1$, the action of ${\tilde{D}}^{(n)}$ as in (7.20) depends on the trajectories of the neighboring particles around the *n*-th one:

$$\begin{aligned} &  \textrm{x}_{n-1}:=x_{n-1}(t+1)> \textrm{x}_{n+1}:=x_{n+1}(t+1),\qquad \\  &  \textrm{x}_{n-1}-i_1=x_{n-1}(t) > \textrm{x}_{n+1}-j_1=x_{n+1}(t), \end{aligned}$$

where $$i_1,j_1\in \left\{ 0,1 \right\} $$.

#### Remark 8.3

Throughout this section, the roman letters $\textrm{x}_k$, $\textrm{x}_k'$, $\textrm{y}_k$, and $\textrm{y}_k'$ stand for positions of particles at specified times, *t* or $t+1$. We employ this convention for shorter notation to avoid repeatedly writing out the times.

Given the new location $\textrm{x}_n'$ of the *n*-th particle at time $t+1$, ${\tilde{D}}^{(n)}$ maps the two-time trajectory of the *n*-th particle,

$$\begin{aligned} \textrm{x}_n-k_1= x_n(t) \le \textrm{x}_n = x_n(t+1), \end{aligned}$$

where $k_1\in \left\{ 0,1 \right\}$, into a random new trajectory

$$\begin{aligned} \textrm{x}_n'-k_1' \le \textrm{x}_n',\qquad k_1'\in \left\{ 0,1 \right\} . \end{aligned}$$

**Fig. 17:**
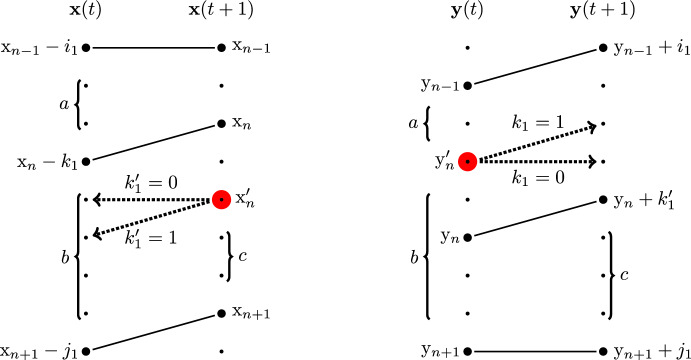
Left: Action of ${\tilde{D}}^{(n)}$ with boundary conditions $i_1=0$, $j_1=1$. Given the location at time $t+1$, the particle $\textrm{x}_n'$ randomly chooses its location at time *t* from the possible locations $\{ \textrm{x}_n',\textrm{x}_n'-1 \}$ with probabilities determined by $p^{\downarrow }_{i_1,j_1}$, independent of $k_1$ unless $\textrm{x}_n'$ is pushed down or blocked. Right: Action of ${\tilde{U}}^{(n)}$ with boundary conditions $i_1=1$, $j_1=0$. Similarly, given the location at time *t*, the particle $\textrm{y}_n'$ randomly chooses its location at time $t+1$ from the possible locations $\{ \textrm{y}_n', \textrm{y}_n'+1 \}$ with probabilities determined by $p^{\uparrow }_{i_1,j_1}$, independent of $k_1'$ unless $\textrm{y}_n'$ is pushed up or blocked

See Fig. 17, left, for an illustration. The coordinates introduced above must satisfy

8.6 $$\begin{aligned} \begin{aligned} \textrm{x}_{n-1}>\textrm{x}_n>\textrm{x}_{n+1},\qquad&\textrm{x}_{n-1}-i_1>\textrm{x}_n-k_1>\textrm{x}_{n+1}-j_1, \\ \textrm{x}_{n-1}>\textrm{x}_n'>\textrm{x}_{n+1},\qquad&\textrm{x}_{n-1}-i_1>\textrm{x}_n'-k_1'>\textrm{x}_{n+1}-j_1, \\ \textrm{x}_n \ge \textrm{x}_n'> \textrm{x}_{n+1},\qquad&\textrm{x}_n-k_1 \ge \textrm{x}_n'-k_1'>\textrm{x}_{n+1}-j_1. \end{aligned} \end{aligned}$$

The inequalities on the last line in (8.6) come from the *q*-beta-binomial specialization of the cross vertex weights $R_{s_n/s_{n-1},s_{n-1},s_n}(\textsf{i}_1,\textsf{i}_2;\textsf{j}_1,\textsf{j}_2)$, which contain the indicator $\textbf{1}_{\textsf{j}_2\le \textsf{j}_1}$, see Proposition 3.5.

The probability to select $k_1'\in \left\{ 0,1 \right\}$ is $p^{\downarrow }_{i_1,j_1}[k_1\rightarrow k_1']$ from (8.5) with parameters $(s_1,s_2)$ replaced by $(s_{n-1},s_n)$, horizontal edge occupation numbers $i_1,j_1,k_1$ specified above, and

$$\begin{aligned} a=\textrm{x}_{n-1}-\textrm{x}_n-1-i_1+k_1,\qquad b=\textrm{x}_{n}-\textrm{x}_{n+1}-1-k_1+j_1,\qquad c=\textrm{x}_n'-\textrm{x}_{n+1}-1, \end{aligned}$$

as indicated in Fig. 17, left.

Under the independent bijectivisation, the probabilities $p^{\downarrow }_{i_1,j_1}[k_1\rightarrow k_1']$ are chosen to be independent (as much as possible) of the old trajectory of the *n*-th particle. More precisely, they depend on the old state $\textrm{x}_n-k_1$ at time *t* through *a*, *b*, but not on the old state $\textrm{x}_n$ at time $t+1$. There are, however, two cases when the value of $k_1'$ is deterministically prescribed by the last inequality in (8.6):

- (*blocking*) If $c=0$ and $j_1=0$, then $k_1'=0$ with probability 1. This means that $\textrm{x}_n'$ is blocked by $\textrm{x}_{n+1}$ from going down due to close proximity.
- (*pushing down*) If $c=b+1-j_1$, then $k_1=1$ and, additionally, $k_1'=1$ with probability 1. This means that $\textrm{x}_n'$ is pushed down by $\textrm{x}_n$ due to close proximity.

We see that in the pushing case, the independent bijectivisation cannot ignore $k_1$ which encoded the old trajectory of the *n*-th particle.

Applying the operators ${\tilde{D}}^{(n)}$ sequentially for $t=M-1,M-2,\ldots ,1,0$, we arrive at the operator $\tilde{H}_n^{\leftarrow }$ for rewriting history. The action of $\tilde{H}_n^{\leftarrow }$ may be viewed as a Markov process running backwards in time, which replaces the old trajectory $\{x_n(t) \}_{0\le t\le M}$ by the new one, $\{x_n'(t) \}_{0\le t\le M}$. The Markov process for building $\{x_n'(t) \}_{0\le t\le M}$ starts from a fixed initial condition $x_n'(M)$ such that $x_{n+1}(M)<x_n'(M)\le x_n(M)$, and evolves in the chamber

$$\begin{aligned} x_{n+1}(t)<x_n'(t)\le x_n(t),\qquad 0\le t\le M. \end{aligned}$$

**Fig. 18:**
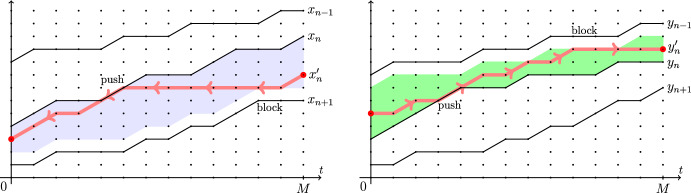
Left: Rewriting history from future to past using the operator ${\tilde{H}}_n^{\leftarrow }$. Right: Rewriting history from past to future using the operator ${\tilde{H}}_n^{\rightarrow }$. In both cases the allowed chamber for the new trajectory of the *n*-th particle is shaded. When the new trajectory reaches the boundary of this chamber, it is pushed or blocked depending on the type of the boundary

The trajectory of the upper neighbor $\{x_{n}(t)\}_{0\le t\le M}$ affects the transition probabilities of $x_n'(t)$ due to the push rule described above. We refer to Fig. 18, left, for an illustration.

Thus, the operator ${\tilde{H}}_n^{\leftarrow }$ satisfies the first part of Corollary 7.11, where all vertex configurations and operators are replaced by their exclusion process counterparts using the gap-particle transformation. Below, in Sect. 8.4, we explicitly describe the Markov process ${\tilde{H}}_n^{\leftarrow }$ for $n=1$ when there is no upper neighbor and the transition probabilities are simpler.

### Rewriting history in particle systems from past to future

The Markov operator ${\tilde{H}}_n^{\rightarrow }$ for rewriting history from past to future is treated very similarly to Markov operator ${\tilde{H}}_n^{\leftarrow }$ for rewriting history from future to past, as described in Sect. 8.2. Here, we only indicate the main notation and definitions. For instance, we denote particle coordinates by $y_j$ instead of $x_j$ to distinguish from the previous subsection.

Let $n\ge 1$ and $|s_{n-1}|\ge |s_n|$ in the stochastic higher spin six vertex model with $J=1$ and $u_i=-\beta s_i$. We first describe the up Markov operator ${\tilde{U}}^{(n)}$ from the independent bijectivisation. For time $0\le t\le M-1$, it depends on the two-time trajectories

$$\begin{aligned} &  \textrm{y}_{n-1}:=y_{n-1}(t)> \textrm{y}_{n+1}:=y_{n+1}(t), \qquad \\  &  \textrm{y}_{n-1}+i_1=y_{n-1}(t+1) > \textrm{y}_{n+1}+j_1=y_{n+1}(t+1), \end{aligned}$$

where $i_1,j_1\in \left\{ 0,1 \right\}$. Given the new location $\textrm{y}_n'$, the operator ${\tilde{U}}^{(n)}$ maps the two-time trajectory of the *n*-th particle, $\textrm{y}_n\le \textrm{y}_n+k_1'$, where $k_1'\in \left\{ 0,1 \right\}$, into a random new trajectory $\textrm{y}_n'\le \textrm{y}_n'+k_1$, where $k_1\in \left\{ 0,1 \right\}$. See Fig. 17, right, for an illustration. All the coordinates must satisfy

8.7 $$\begin{aligned} \begin{aligned} \textrm{y}_{n-1}>\textrm{y}_n>\textrm{y}_{n+1},\qquad&\textrm{y}_{n-1}+i_1>\textrm{y}_n+k_1'>\textrm{y}_{n+1}+j_1,\\ \textrm{y}_{n-1}>\textrm{y}_n'>\textrm{y}_{n+1},\qquad&\textrm{y}_{n-1}+i_1>\textrm{y}_n'+k_1>\textrm{y}_{n+1}+j_1,\\ \textrm{y}_{n-1}> \textrm{y}_n'\ge \textrm{y}_n,\qquad&\textrm{y}_{n-1}+i_1 > \textrm{y}_n'+k_1 \ge \textrm{y}_n+k_1'. \end{aligned} \end{aligned}$$

The probability to select $k_1\in \left\{ 0,1 \right\}$ is $p^{\uparrow }_{i_1,j_1}[k_1'\rightarrow k_1]$ given in (8.5) with parameters $(s_1,s_2)$ replaced by $(s_{n-1},s_n)$, horizontal edge occupation numbers $i_1,j_1,k_1'$ specified above, and

$$\begin{aligned} a=\textrm{y}_{n-1}-\textrm{y}_n'-1,\qquad b=\textrm{y}_n'-\textrm{y}_{n+1}-1,\qquad c=\textrm{y}_n-\textrm{y}_{n+1}-1+k_1'-j_1 \end{aligned}$$

as indicated in Fig. 17, right. There are also blocking and pushing mechanisms present:

- (*blocking*) If $a=0$ and $i_1=0$, then $k_1=0$ with probability 1. This means that $\textrm{y}_n'$ is blocked and cannot go up due to close proximity to $\textrm{y}_{n-1}$.
- (*pushing up*) If $c=b+1-j_1$, then $k_1'=1$ and, additionally, $k_1=1$ with probability 1. This means that $\textrm{y}_n'$ is pushed up by $\textrm{y}_n$ due to close proximity.

Applying the operators ${\tilde{U}}^{(n)}$ sequentially for $t=0,1,\ldots ,M-1$, we arrive at the Markov operator $\tilde{H}_n^{\rightarrow }$ for rewriting history from past to future. Its action may be viewed as a Markov process which builds the new trajectory $\{y_n'(t)\}_{0\le t\le M}$ of the *n*-th particle which lies in the chamber

$$\begin{aligned} y_n(t)\le y_n'(t)<y_{n-1}(t),\qquad 0\le t\le M. \end{aligned}$$

This new trajectory, which replaces the old trajectory $\{y_n(t)\}_{0\le t\le M}$, starts from a fixed initial condition $y_n'(0)$, and its law depends on the trajectories of the particles with numbers $n-1,n,n+1$. See Fig. 18, right, for an illustration.

Thus, the operator ${\tilde{H}}_n^{\rightarrow }$ satisfies the second part of Corollary 7.11, where all vertex configurations and operators are replaced by their exclusion process counterparts using the gap-particle transformation. Below, in Sect. 8.4, we explicitly describe ${\tilde{H}}_n^{\rightarrow }$ for $n=1$ when the transition probabilities are simpler.

### Resampling the first particle

Let us illustrate the general results of the previous Sect. 8.2 and 8.3 and consider the case $n=1$, that is, the system of two particle. Let $\textbf{x}(t)=(x_1(t),x_2(t))$, $x_1(t)>x_2(t)$, be the first two particles in the system given at the beginning of Sect. 8.2. More precisely, $\textbf{x}(t)$ corresponds (via the gap-particle transformation, see Definition 2.4) to the stochastic higher spin six vertex model with $J=1$ and the parameters

8.8 $$\begin{aligned} u_i=-\beta s_i,\qquad \beta>0, \qquad |s_0|>|s_1|. \end{aligned}$$

We will omit *J* and $\beta$ in the notation and simply say that $\textbf{x}(t)$ has parameters $(s_0,s_1)$. Let also $\textbf{y}(t)=(y_1(t),y_2(t))$ be the process with the swapped parameters $(s_1,s_0)$. We will describe two couplings between $\textbf{x}(t)$ and $\textbf{y}(t)$. Similar couplings may be explicitly written down for any number of particles, but the advantage for $n=1$ is that the transition probabilities have a simple form.

We start with rewriting history from future to past. Let us denote

8.9 $$\begin{aligned} \texttt{d}^0_{b,c} = \frac{(1+\beta s_0^2 q^c)(1-q^{b+1-c})}{\beta (s_0^2-s_1^2 q^{b-c})(1-q^c)}, \qquad \texttt{d}^1_{b,c} = \frac{(1-s_0^2q^c)(1-q^{b-c})}{(\beta +q^c)(s_0^2-s_1^2 q^{b-c-1})}, \end{aligned}$$

where $b,c\in \mathbb {Z}_{\ge 0}$. The quantities (8.9) take values in $[0,+\infty ]$. In particular, we may have $\texttt{d}^0_{b,0}=+\infty$ and $\texttt{d}^0_{b,b+1}=\texttt{d}^1_{b,b}=0$, which will respectively correspond to blocking and pushing down as in Sect. 8.2.

Fix $M\in \mathbb {Z}_{\ge 1}$. Assume that we are given a chamber $\{x_1(t)>x_2(t)\}_{0\le t\le M}$, and also $x_1'(M)$ with $x_1(M)\ge x_1'(M)>x_2(M)$. The process ${\tilde{H}}_1^{\leftarrow }$ is a random walk $x_1'(t)$ in a chamber (i.e. $x_1(t)\ge x_1'(t)>x_2(t)$ for all *t*) which is started from $x_1'(M)$ and runs in reverse time $t=M-1,M-2,\ldots ,1,0$. See Fig. 18, left with $n=1$ and $x_0=+\infty$, for an illustration. During time step $t+1\rightarrow t$, this random walk takes steps 0 or $-1$ with probabilities

8.10 $$\begin{aligned} \frac{\texttt{d}^{j_1}_{b,c}}{1+\texttt{d}^{j_1}_{b,c}} \quad \text {and}\quad \frac{1}{1+\texttt{d}^{j_1}_{b,c}}, \end{aligned}$$

respectively, where

$$\begin{aligned} &  b=x_1(t)-x_2(t)-1, \qquad c=x_1'(t+1)-x_2(t+1)-1, \qquad \\  &  j_1=x_2(t+1)-x_2(t)\in \left\{ 0,1 \right\} , \end{aligned}$$

as in Fig. 17, left, with $a=+\infty$.

#### Proposition 8.4

Fix $M\in \mathbb {Z}_{\ge 1}$. Let $\textbf{x}(t)$ be the system as above, with parameters $(s_0,s_1)$ satisfying (8.8) and initial conditions so that $x_1(0)>x_2(0)$. Also, let $x_1'(M)$ be chosen from the probability distribution

8.11 $$\begin{aligned} \varphi _{q,s_1/s_0^2,s_1^2}(x_1'(M)-x_2(M)-1\mid x_1(M)-x_2(M)-1). \end{aligned}$$

Moreover, let $x_1'(t)$ be the random walk in the chamber, $x_1(t)\ge x_1'(t)>x_2(t)$, with transition probabilities (8.10) as above. Then, the joint distribution of the new process $\{(x_1'(t),x_2(t))\}_{0\le t\le M}$ is equal to the distribution of the process $\textbf{y}(t)$ with parameters $(s_1,s_0)$ started from the initial condition $y_1'(0)>x_2(0)$, where $y_1'(0)$ is random and chosen from

8.12 $$\begin{aligned} \varphi _{q,s_1/s_0^2,s_1^2}(y_1'(0)-x_2(0)-1\mid x_1(0)-x_2(0)-1). \end{aligned}$$

#### Proof

First, note that the distributions (8.11), (8.12) are precisely given by the corresponding application of the Markov swap operator $\tilde{P}^{(1)}$. Then, by the first part of Theorem 7.9, it suffices to show that

8.13 $$\begin{aligned} \texttt{d}^{0}_{b,c} = \frac{w^{\textrm{RHS}}_{*,0}(0)}{w^{\textrm{RHS}}_{*,0}(1)},\qquad \texttt{d}^{0}_{b,c}= \frac{w^{\textrm{RHS}}_{*,1}(0)}{w^{\textrm{RHS}}_{*,1}(1)}, \end{aligned}$$

using the notation of Sect. 8.1. These identities are checked in a straightforward way using the vertex weights in Fig. 16 with $(s_1,s_2)$ renamed to $(s_0,s_1)$. Thus, we have

8.14 $$\begin{aligned} \frac{\texttt{d}^{j_1}_{b,c}}{1+\texttt{d}^{j_1}_{b,c}} = p^{\downarrow }_{*,j_1}[k_1\rightarrow 0], \qquad \frac{1}{1+\texttt{d}^{j_1}_{b,c}} = p^{\downarrow }_{*,j_1}[k_1\rightarrow 1]. \end{aligned}$$

Recall that star in (8.13), (8.14) means that there is no dependence on $i_1$ since $a=+\infty$. It follows that the random walk $x_1'(t)$ with transition probabilities (8.10) is indeed the process ${\tilde{H}}_1^{\leftarrow }$. This completes the proof. $\square$

Let us similarly write down the transition probabilities for the random walk ${\tilde{H}}_1^{\rightarrow }$ in the chamber $\left\{ y_1(t)>y_2(t) \right\} _{0\le t\le M}$ started from an initial condition $y_1'(0)$. For $b,c\in \mathbb {Z}_{\ge 0}$, denote:

8.15 $$\begin{aligned} \texttt{u}^0_{b,c} = \frac{(1+\beta s_1^2 q^b)(1-q^{b+1-c})}{\beta (s_0^2-s_1^2 q^{b-c})(1-q^{b+1})}, \qquad \texttt{u}^1_{b,c} = \frac{ (1-s_1^2 q^{b-1})(1-q^{b-c}) }{ (\beta +q^{b})(s_0^2-s_1^2 q^{b-c-1}) }. \end{aligned}$$

These quantities take values in $[0,+\infty )$. There is no $+\infty$ values since there is no blocking because $a=+\infty$ or, equivalently, $y_0 = + \infty$. In particular, we have $\texttt{u}^0_{b,b+1}=\texttt{u}^1_{b,b}=0$, corresponding to pushing up rules as in Sect. 8.3.

Fix a trajectory of the two-particle system $\{(y_1(t),y_2(t))\}_{t\ge 0}$, where $y_1(t)>y_2(t)$. Moreover, fix $y_1'(0)$ such that $y_1'(0)\ge y_1(0)$. The process $\tilde{H}_1^{\rightarrow }$ is a random walk $y_1'(t)$ with $y_1'(t)\ge y_1(t)$ for all *t*, which runs in forwards time $t=0,1,\ldots$. See Fig. 18, right with $n=1$ and $y_0=+\infty$, for an illustration. The walk starts from $y_1'(0)$ and, for the transition $t\rightarrow t+1$, it takes steps 0 or $+1$ with probabilities

8.16 $$\begin{aligned} \frac{\texttt{u}^{j_1}_{b,c}}{1+\texttt{u}^{j_1}_{b,c}} \quad \text {and}\quad \frac{1}{1+\texttt{u}^{j_1}_{b,c}}, \end{aligned}$$

respectively, where

$$\begin{aligned} b=y_1'(t)-y_2(t)-1, \qquad c=y_1(t+1)-y_2(t+1)-1, \qquad j_1=y_2(t+1)-y_2(t), \end{aligned}$$

as in Fig. 17, right, with $a=+\infty$. The next statement is proven in the same way as Proposition 8.4:

#### Proposition 8.5

Let $y_1'(0)>y_2(0)$ be fixed, and take $y_1(0)$, with $y_2(0)<y_1(0)\le y_1'(0)$, to be random and with distribution given by

$$\begin{aligned} \varphi _{q,s_1/s_0^2,s_1^2}(y_1(0)-y_2(0)-1\mid y_1'(0)-y_2(0)-1). \end{aligned}$$

Let $\textbf{y}(t)$ be the two-particle system with initial condition $(y_1(0),y_2(0))$ and parameters $(s_1,s_0)$ satisfying (8.8). Given the trajectory $\{\textbf{y}(t)\}_{\ge 0}$, construct the random walk $\{y_1'(t)\}_{t\ge 0}$ from the initial condition $y_1'(0)$ with $y_1'(t)\ge y_1(t)$ and transition probabilities given by (8.16). Then, the joint distribution of the new process $\{(y_1'(t),y_2(t))\}_{t\ge 0}$ is equal to the joint distribution of the process $\textbf{x}(t)$ with parameters $(s_0,s_1)$ with initial condition $y_1'(0)>y_2(0)$.

We generalize a certain parameter symmetry for the partincle systems using the couplings between the two-particle systems $\textbf{x}(t)$ with parameters $(s_0,s_1)$ and $\textbf{y}(t)$ with parameters $(s_1,s_0)$ in Propositions 8.4 and 8.5. For instance, take both $\textbf{x}(t)$ and $\textbf{y}(t)$ with step initial conditions $\textbf{x}_{step}$, i.e. $x_1(0)=y_1(0)=-1$ and $x_2(0)=y_2(0)=-2$. Then, the distributions of the trajectories of the second particle, $\{x_2(t)\}_{t\ge 0}$ and $\{y_2(t)\}_{t\ge 0}$, are the same. In particular, one may show that the distribution of the the second particle is a *symmetric* function on the parameters $(s_0, s_1)$, without using a coupling argument, making the previous statement true. On the other hand, this symmetry breaks when the initial configuration is not $\textbf{x}_{step}$. The following statement restores (in a stochastic way) the symmetry for general initial configurations:

#### Corollary 8.6

Fix $x_1(0)>x_2(0)$. Let $\textbf{x}(t)$ be the two-particle system with parameters $(s_0,s_1)$, $|s_0|>|s_1|$, started from $(x_1(0),x_2(0))$. Let $y_1'(0)$, where $x_2(0)<y_1'(0)\le x_1(0)$ be random and distributed as

$$\begin{aligned} \varphi _{q,s_1/s_0^2,s_1^2}(y_1'(0)-x_2(0)-1\mid x_1(0)-x_2(0)-1). \end{aligned}$$

Start $\textbf{y}(t)$ with parameters $(s_1,s_0)$ from the random initial configuration $(y_1'(0),x_2(0))$. Then the distributions of the trajectories second particle in both systems, $\{x_2(t)\}_{t\ge 0}$ and $\{y_2(t)\}_{t\ge 0}$, coincide.

#### Proof

This follows from the history rewriting processes in either of Propositions 8.4 and 8.5 since both of these processes keep the trajectory of the second particle intact. $\square$

Let us specialize to $q=0$. We consider the random walk $\tilde{H}_1^{\leftarrow }$ for rewriting history from future to past, described before Propositions 8.4. One may similarly specialize ${\tilde{H}}_1^{\rightarrow }$, but we omit this for brevity. The quantities (8.9), for $q=0$, specialize as follows

8.17 $$\begin{aligned} \texttt{d}^0_{b,c} \big \vert _{q=0} = {\left\{ \begin{array}{ll} +\infty ,& c=0;\\ \frac{1}{\beta s_0^2},& 1\le c\le b-1;\\ \frac{1}{\beta (s_0^2-s_1^2)},& c=b;\\ 0,& c=b+1, \end{array}\right. } \qquad \texttt{d}^1_{b,c} \big \vert _{q=0} = {\left\{ \begin{array}{ll} \frac{1-s_0^2}{(1+\beta )s_0^2},& c=0;\\ \frac{1}{\beta s_0^2}& 1\le c\le b-2;\\ \frac{1}{\beta (s_0^2-s_1^2)}& c=b-1;\\ 0,& c=b. \end{array}\right. }\nonumber \\ \end{aligned}$$

**Fig. 19:**
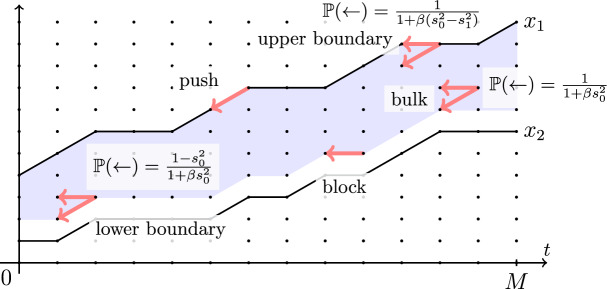
Transition probabilities for the simple random walk $x_1'(t)$ for rewriting history from future to past in the specialization $q=0$. This walk runs in reverse time inside the chamber $x_1(t)\ge x_1'(t)>x_2(t)$. Its transition probabilities differ on the boundary of the chamber, compared to the bulk. In the figure, we only list the probability of the step 0, with the probability of the step $-1$ determined by the complement formula

It follows that $x_1'(t)$, the process in reversed time living in the chamber $x_1(t)\ge x_1'(t)>x_2(t)$, is a simple random walk with location-dependent transition probabilities. Namely, in the *bulk* of the chamber, it takes a step $-1$ with probability $\frac{1}{1+1 / (\beta s_0^2)} =\frac{\beta s_0^2}{1+\beta s_0^2}$ and a step 0 with the complementary probability $\frac{1}{1+\beta s_0^2}$. At the boundary of the chamber, the probabilities need to be suitably modified, see Fig. 19 for an illustration of all cases which are determined from (8.17).

## Bijectivisation and Rewriting History in Continuous Time

In this section, we construct the rewriting history processes for *q*-TASEP and TASEP evolving in continuous time using a bijectivisation. We view the *q*-TASEP as a continuous time limit of the system with $J=1$ and $u_i=-\beta s_i$ considered in Sect. 8. Thus, we deal with is a specialization of the independent bijectivisation in Sect. 8.1.

### Limit to continuous time *q*-TASEP

Let us take a continuous time limit of the vertex model from Sect. 8.2. Recall that we had set $J=1$ and $u_i=-\beta s_i$, where $\beta >0$. For this model, the stochastic vertex weights and the cross vertex weights for the vertical Yang-Baxter equation are given in Fig. 16. Now, consider the following limit of the parameters:

- First, set $\beta s_i^2=\varepsilon \alpha _i$, for all *i*, where $\alpha _i>0$, and $\varepsilon >0$ is fixed for now.
- Send $\beta \rightarrow +\infty$ and $s_i\rightarrow 0$ so that $\varepsilon \alpha _i>0$ is fixed.
- After this, take the limit as $\varepsilon \rightarrow 0$ and rescale time from discrete to continuous as $t=\lfloor \textsf{t}/\varepsilon \rfloor$, where $\textsf{t}\in \mathbb {R}_{\ge 0}$ is the new continuous time.

**Fig. 20:**
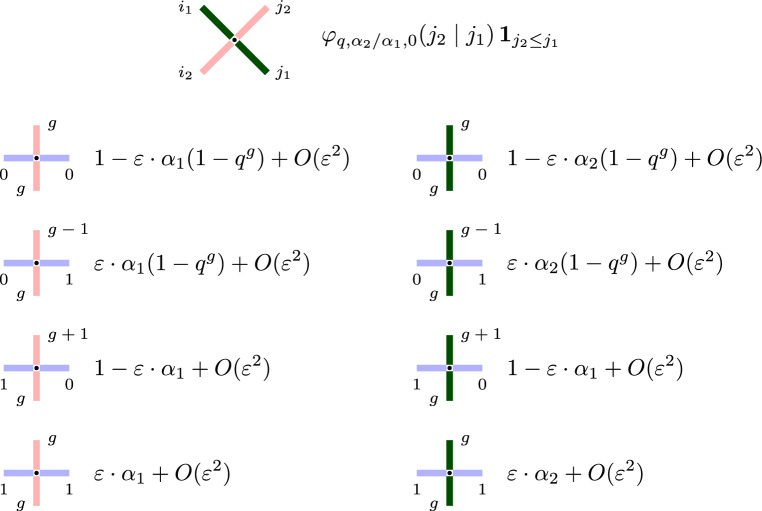
Expansions as $\varepsilon \rightarrow 0$ of the vertex weights entering the Yang-Baxter equation for the continuous time *q*-TASEP, where the time is scaled proportionally to $\varepsilon ^{-1}$. The cross vertex weights are nonnegative when $\alpha _1\ge \alpha _2>0$

These operations turn the stochastic higher spin six vertex model into the continuous time stochastic *q*-Boson model [SW98, BC14, BCPS15] with inhomogeneous rates $\alpha _i$. Indeed, the $\varepsilon \rightarrow 0$ expansions of all the vertex weights in the last operation are given in Fig. 20. We see that the cross vertex weights do not depend on $\varepsilon$, while the weights in the vertex model itself are of order $O(\varepsilon )$ or $1-O(\varepsilon )$. The weights of type $(g,0;g-1,1)$ correspond to the jump rates in the *q*-Boson model. More precisely, each stack of $g_n$ vertical arrows at location $n\in \mathbb {Z}_{\ge 0}$ (with the agreement $g_0=+\infty$) emits one horizontal arrow at rate $\alpha _n(1-q^{g_n})$. This horizontal arrow instantaneously travels horizontal distance 1 and joins the next stack of arrows at location $n+1$. This is because the probability to travel distance at least 2 is proportional to $\varepsilon ^2$, which is negligible in the continuous time limit.

The *q*-Boson system corresponds to the continuous time *q*-TASEP, where the particle $x_n$ has speed $\alpha _{n-1}$ for $n\in \mathbb {Z}_{\ge 0}$, via the gap-particle transformation given in Definition 2.4. That is, each $x_n$ jumps to the right by one at rate $\alpha _{n-1}(1-q^{g_{n-1}})$, where $g_{n-1}=x_{n-1}-x_n-1$.

### Independent bijectivisation in continuous time

Let us now write down the $\varepsilon \rightarrow 0$ expansions, under the setting of Sect. 8.1, of the transition probabilities

9.1 $$\begin{aligned} p^{\downarrow }_{i_1,j_1}[*\rightarrow 0],\qquad p^{\downarrow }_{i_1,j_1}[*\rightarrow 1],\qquad p^{\uparrow }_{i_1,j_1}[*\rightarrow 0],\qquad p^{\uparrow }_{i_1,j_1}[*\rightarrow 1] \end{aligned}$$

given by (8.5), after performing the first two steps of the specialization from Sect. 9.1. Here and below “$*$” means that the transition does not depend on the previous state as much as possible, which is a feature of the independent bijectivisation. However, see the blocking and pushing mechanisms described in Sects. 8.2 and 8.3. The resulting expansions of (9.1) would depend on *q* and the spectral parameters $\alpha _1\ge \alpha _2 > 0$. We also continue to use the notation (8.1)–(8.3) for the boundary conditions in the Yang-Baxter equation.

#### Proposition 9.1

Given the conventions explained before the proposition, we have the following $\varepsilon \rightarrow 0$ expansions of the down transition probabilities:

9.2 $$\begin{aligned} p^{\downarrow }_{00}[*\rightarrow 1]&= \textbf{1}_{c=b+1}+ \varepsilon \hspace{1pt}\textbf{1}_{c\le b}\hspace{1pt}\frac{(\alpha _1-\alpha _2 q^{b-c})(1-q^{a+b+1-c})(1-q^c)}{1-q^{b-c+1}} +O(\varepsilon ^2). \end{aligned}$$

9.3 $$\begin{aligned} p^{\downarrow }_{01}[*\rightarrow 1]&= \textbf{1}_{c=b}+ \varepsilon \hspace{1pt}\textbf{1}_{c\le b-1}\hspace{1pt}\frac{(\alpha _1-\alpha _2 q^{b-c-1})(1-q^{a+b-c})}{1-q^{b-c}} +O(\varepsilon ^2); \end{aligned}$$

9.4 $$\begin{aligned} p^{\downarrow }_{10}[*\rightarrow 1]&= \textbf{1}_{c=b+1}+ \varepsilon \hspace{1pt}\textbf{1}_{c\le b} \hspace{1pt}\frac{(\alpha _1-\alpha _2 q^{b-c})(1-q^c)}{1-q^{b+1-c}} +O(\varepsilon ^2); \end{aligned}$$

9.5 $$\begin{aligned} p^{\downarrow }_{11}[*\rightarrow 1]&= \textbf{1}_{c=b}+ \varepsilon \hspace{1pt}\textbf{1}_{c\le b-1} \hspace{1pt}\frac{\alpha _1-\alpha _2 q^{b-c-1}}{1-q^{b-c}}+O(\varepsilon ^2). \end{aligned}$$

For the up transition probabilities, we have

9.6 $$\begin{aligned} p^{\uparrow }_{00}[*\rightarrow 1]&= \textbf{1}_{c=b+1} +\varepsilon \hspace{1pt}\textbf{1}_{c\le b} \hspace{1pt}\frac{(\alpha _1-\alpha _2 q^{b-c})(1-q^a)(1-q^{b+1})}{1-q^{b-c+1}} +O(\varepsilon ^2); \end{aligned}$$

9.7 $$\begin{aligned} p^{\uparrow }_{01}[*\rightarrow 1]&= \textbf{1}_{c=b} +\varepsilon \hspace{1pt}\textbf{1}_{c\le b-1} \hspace{1pt}\frac{(\alpha _1-\alpha _2 q^{b-c-1})(1-q^a)}{1-q^{b-c}} +O(\varepsilon ^2); \end{aligned}$$

9.8 $$\begin{aligned} p^{\uparrow }_{10}[*\rightarrow 1]&= \textbf{1}_{c=b+1} +\varepsilon \hspace{1pt}\textbf{1}_{c\le b} \hspace{1pt}\frac{(\alpha _1-\alpha _2 q^{b-c})(1-q^{b+1})}{1-q^{b-c+1}} +O(\varepsilon ^2); \end{aligned}$$

9.9 $$\begin{aligned} p^{\uparrow }_{11}[*\rightarrow 1]&= \textbf{1}_{c=b}+ \varepsilon \hspace{1pt}\textbf{1}_{c\le b-1} \hspace{1pt}\frac{\alpha _1-\alpha _2 q^{b-c-1}}{1-q^{b-c}} +O(\varepsilon ^2) . \end{aligned}$$

In all cases, the complementary probabilities follow and are determined by the complement formula, e.g. $p^{\downarrow }_{i_1,j_1}[*\rightarrow 0]=1-p^{\downarrow }_{i_1,j_1}[*\rightarrow 1]$. The parameters *a*, *b*, *c* in formulas (9.2)–(9.9) satisfy $a,b\ge 0$ and $0\le c\le b+\min (a+i_1,1)-j_1$.

#### Proof

These expansions are obtained in a straightforward way using Definition 8.1 and the explicit formulas for the vertex weights after performing the first two steps of the specialization from Sect. 9.1; see Fig. 20. $\square$

In continuous time, note that during each time moment there is *at most one jump* of any of the particles, both before and after applying a rewriting history operator. This means that we can eliminate the boundary conditions $i_1=j_1=1$ which never occur in continuous time.

Next, consider the case when $i_1+j_1=1$. The order 1 terms in (9.3)–(9.4) and (9.7)–(9.8) occur only when both the old trajectory of the *n*-th particle, and one of the particles $x_{n\pm 1}$ jump at the same time, which is impossible in continuous time. The order $\varepsilon$ terms in (9.3)–(9.4) and (9.7)–(9.8) correspond to both the new trajectory of $x_n$ and one of the particles $x_{n\pm 1}$ jumping at the same time, which is also impossible in continuous time. It follows multiple events with probability of order $\varepsilon$ occur when $i_1+j_1=1$. Thus, we have that the case $i_1+j_1=1$ cannot lead to new jump in the new trajectory of the *n*-th particle since such a jump would be an event of probability $O(\varepsilon ^2)$, which vanishes in the continuous time limit.

Therefore, the independent bijectivisation in the continuous time limit is *completely determined* by the expansions (9.2) and (9.6) for $i_1=j_1=0$. In the rest of the current Sect. 9, we use this fact to describe the rewriting history processes ${\tilde{H}}_n^{\leftarrow }$ and $\tilde{H}_n^{\rightarrow }$ for the continuous time *q*-TASEP and TASEP.

### Rewriting history from future to past for a parameter swap in *q*-TASEP

Here and in the next Sect. 9.4, we describe the *q*-TASEP’s rewriting history processes $\tilde{H}_n^{\leftarrow }$ and ${\tilde{H}}_n^{\rightarrow }$ from Definition 7.10. These processes are based on the independent bijectivisation and are determined by the jump rates coming from (9.2) and (9.6), respectively.

Let us start with the process ${\tilde{H}}_n^{\leftarrow }$ of rewriting history from future to past. Fix $\textsf{M}\in \mathbb {R}_{\ge 0}$, $n\in \mathbb {Z}_{\ge 1}$, and two speeds $\alpha _{n-1}>\alpha _n>0$. Assume we have three trajectories of consecutive particles,

$$\begin{aligned} x_{n-1}(\textsf{t})>x_{n}(\textsf{t})>x_{n+1}(\textsf{t}),\qquad 0\le \textsf{t}\le \textsf{M} \end{aligned}$$

and a starting point $x_n'(\textsf{M})$ with

$$\begin{aligned} x_n(\textsf{M})\ge x_n'(\textsf{M})> x_{n+1}(\textsf{M}). \end{aligned}$$

The process $x_n'(\textsf{t})$ starts from $x_n'(\textsf{M})$ and runs in reverse time in the chamber $x_{n}(\textsf{t})\ge x_{n}'(\textsf{t})>x_{n+1}(\textsf{t})$, making jumps down by 1 in continuous time with rate

9.10 $$\begin{aligned} \textrm{rate}^{\leftarrow }_{n;\alpha _{n-1},\alpha _n}= \frac{(\alpha _{n-1}-\alpha _n q^{b-c})(1-q^{a+b+1-c})(1-q^c)}{1-q^{b-c+1}}, \end{aligned}$$

where (cf. Figure 17, left)

9.11 $$\begin{aligned} &  a :=x_{n-1}(\textsf{t}-)-x_n(\textsf{t}-)-1, \quad b :=x_n(\textsf{t}-)-x_{n+1}(\textsf{t}-)-1, \quad \nonumber \\  &  c :=x_n'(\textsf{t})-x_{n+1}(\textsf{t})-1. \end{aligned}$$

**Fig. 21:**
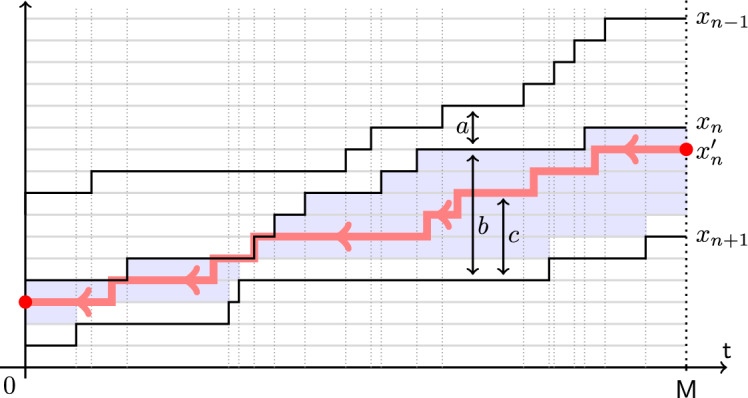
The process ${\tilde{H}}_n^{\leftarrow }$ of rewriting history from future to past for the continuous time *q*-TASEP and the parameter swap $\alpha _{n-1}\leftrightarrow \alpha _n$. The allowed chamber for the new trajectory $x_n'(\textsf{t})$ is shaded. The quantities (9.11) indicated in the figure for a particular time interval are equal to $a=1,b=5,c=3$ so that the jump rate (9.10) at that particular time interval is equal to $(\alpha _{n-1}-\alpha _n q^{2})(1-q^4)$

Note that the rate (9.10) is a piecewise constant function of the time $\textsf{t}$, and the rate changes whenever one of the particles $x_{n-1},x_{n}$, or $x_{n+1}$ makes a jump. The particle $x_n'$ is *blocked* from jumping down by $x_{n+1}$, i.e. $\textrm{rate}^{\leftarrow }_{n;\alpha _{n-1},\alpha _n}=0$, when $c=0$. The particle $x_n'$ is *pushed down* by a jump of $x_n$, i.e. $\textrm{rate}^{\leftarrow }_{n;\alpha _{n-1},\alpha _n}=+\infty$, when $c=b+1$. Note that $a+b+1-c$ is always positive, so the factor $1-q^{a+b+1-c}$ does not vanish. The blocking and pushing mechanisms are similar to the discrete time case from Sect. 8.2. See Fig. 21 for an illustration.

The process ${\tilde{H}}_n^{\leftarrow }$ described above produces a coupling of the trajectories of the *q*-TASEPs with speed sequences differing by the swap $\alpha _{n-1}\leftrightarrow \alpha _n$. Recall that, for the *q*-TASEP, the Markov swap operator ${\tilde{P}}^{(n)}$ depending on the ratio $0\le \alpha _n/\alpha _{n-1}\le 1$ acts on $\mathscr {X}$ by moving the single particle $x_n$ into a random new location $x_n'$ with probability

9.12 $$\begin{aligned} \varphi _{q,\alpha _{n}/\alpha _{n-1},0}(x_n'-x_{n+1}-1 \mid x_n-x_{n+1}-1). \end{aligned}$$

Let $\textbf{x}(0)\in \mathscr {X}$ be an initial condition, and $\varvec{\alpha }=(\alpha _0,\alpha _1,\ldots )$ be a sequence of particle speeds such that $\alpha _{n-1}>\alpha _n$. Let $\{\textbf{x}(\textsf{t})\}_{0\le \textsf{t}\le \textsf{M}}$ be the continuous time *q*-TASEP started from $\textbf{x}(0)$ with speeds $\varvec{\alpha }$. Let also $\{\textbf{y}(\textsf{t})\}_{0\le \textsf{t}\le \textsf{M}}$ be the continuous time *q*-TASEP started from the random initial condition $\delta _{\textbf{x}(0)}\tilde{P}^{(n)}$ and evolving with the speeds $\sigma _{n-1}\varvec{\alpha }$, where $\sigma _{n-1}$ is the elementary transposition $\alpha _{n-1}\leftrightarrow \alpha _n$.

#### Proposition 9.2

Given the notation above, let $\textbf{x}'(\textsf{M})$ be obtained from $\textbf{x}(\textsf{M})$ by the action of ${\tilde{P}}^{(n)}$, that is, by randomly moving $x_n(\textsf{M})$ to $x_n'(\textsf{M})$ with probability (9.12). Given the trajectories of the particles $x_j(\textsf{t})$, $j=n-1,n,n+1$, replace the old trajectory $x_n(\textsf{t})$ by the new one $x_n'(\textsf{t})$ constructed from the process ${\tilde{H}}_n^{\leftarrow }$ started from $x_n'(\textsf{M})$ and running in reverse time. Then, the resulting trajectory of the whole process $\{x_1(\textsf{t}),\ldots ,x_{n-1}(\textsf{t}),x_n'(\textsf{t}),x_{n+1}(\textsf{t}),\ldots \}_{0\le \textsf{t}\le \textsf{M}}$ is equal in distribution to the trajectory of the process $\{\textbf{y}(\textsf{t})\}_{0\le \textsf{t}\le \textsf{M}}$.

#### Proof

This is a continuous time limit of the general Theorem 7.9 and Corollary 7.11. $\square$

#### Remark 9.3

*(TASEP specialization of* ${\tilde{H}}_n^{\leftarrow }$*)* The *q*-TASEP turns into the TASEP when $q=0$. In that case, the dynamics $\tilde{H}_n^{\leftarrow }$ simplifies. Namely, the blocking and pushing mechanisms stay the same, and the jump rates (9.10) become

9.13 $$\begin{aligned} \textrm{rate}^{\leftarrow }_{n;\alpha _{n-1},\alpha _n}\Big \vert _{q=0}= \alpha _{n-1}-\alpha _n \textbf{1}_{b=c}. \end{aligned}$$

Thus, the process $x_n'(\textsf{t})$ is a Poisson random walk in the chamber $x_{n}(\textsf{t})\ge x_{n}'(\textsf{t})>x_{n+1}(\textsf{t})$, running in reverse time and jumping down with rate $\alpha _{n-1}$ in the bulk and $\alpha _{n-1}-\alpha _n$ at the top boundary of the chamber.

Clearly, Proposition 9.2 for $q=0$ holds for the process ${\tilde{H}}_n^{\leftarrow }$ with the jump rates (9.13). This proposition for $q=0$ and $n=1$ immediately implies Theorem 1.2 from the Introduction.

### Rewriting history from past to future for a parameter swap in *q*-TASEP

Let us now consider the process ${\tilde{H}}_n^{\rightarrow }$ of rewriting history from past to future. Fix $n\in \mathbb {Z}_{\ge 1}$ and two speeds $\alpha _{n-1}>\alpha _n>0$. Assume we have three trajectories of consecutive particles,

$$\begin{aligned} y_{n-1}(\textsf{t})>y_{n}(\textsf{t})>y_{n+1}(\textsf{t}), \end{aligned}$$

where $\textsf{t}$ runs over $\mathbb {R}_{\ge 0}$, and a starting point $y_n'(0)$ so that $y_n(0)\le y_n'(0)< y_{n-1}(0)$. The process $y_n'(\textsf{t})$ starts from $y_n'(0)$ and runs in forward time $\textsf{t}\in \mathbb {R}_{\ge 0}$ in the chamber $y_n(\textsf{t})\le y_n'(\textsf{t})< y_{n-1}(\textsf{t})$. In continuous time, the location of $y_n'(\textsf{t})$ jumps up by 1 with rate

9.14 $$\begin{aligned} \textrm{rate}^{\rightarrow }_{n;\alpha _{n-1},\alpha _n}= \frac{(\alpha _{n-1}-\alpha _n q^{b-c})(1-q^a)(1-q^{b+1})}{1-q^{b-c+1}}, \end{aligned}$$

where (cf. Figure 17, right)

9.15 $$\begin{aligned} &  a :=y_{n-1}(\textsf{t}-)-y_n'(\textsf{t}-)-1, \quad b :=y_n'(\textsf{t}-)-y_{n+1}(\textsf{t}-)-1, \quad \nonumber \\  &  c :=y_n(\textsf{t})-y_{n+1}(\textsf{t})-1. \end{aligned}$$

**Fig. 22:**
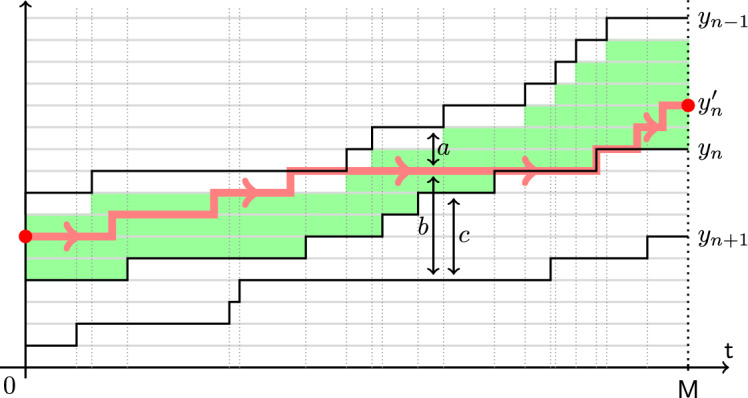
The process ${\tilde{H}}_n^{\rightarrow }$ of rewriting history from past to future for the continuous time *q*-TASEP and the parameter swap $\alpha _{n-1}\leftrightarrow \alpha _n$. The allowed chamber for the new trajectory $y_n'(\textsf{t})$ is shaded. The quantities (9.15) indicated in the figure for a particular time interval are equal to $a=1,b=4,c=3$ so that the jump rate (9.14) at the indicated time is equal to $(\alpha _{n-1}-\alpha _{n} q)(1-q)(1-q^{5})/(1-q^{2})$

The rate (9.14) is a piecewise constant function of the time $\textsf{t}$, and the rate changes whenever one of the particles $y_{n-1},y_{n}$, or $y_{n+1}$ makes a jump. The particle $y_n'$ is *blocked* from jumping up by $y_{n-1}$, i.e. $\textrm{rate}^{\rightarrow }_{n;\alpha _{n-1},\alpha _n}=0$, when $a=0$. The particle $y_n'$ is *pushed up* by a jump of $y_n$, i.e. $\textrm{rate}^{\rightarrow }_{n;\alpha _{n-1},\alpha _n}=+\infty$, when $c=b+1$. The blocking and pushing mechanisms are similar to the discrete time ones from Sect. 8.3. See Fig. 22 for an illustration.

The process ${\tilde{H}}_n^{\rightarrow }$ produces a coupling of the trajectories of the *q*-TASEPs in which speeds differ by the swap $\alpha _{n-1}\leftrightarrow \alpha _n$. Recall the notation before Proposition 9.2. The process $\textbf{x}(\textsf{t})$ is the continuous time *q*-TASEP started from a fixed initial configuration $\textbf{x}(0)\in \mathscr {X}$ and evolves with the particle speeds $\varvec{\alpha }$ so that $\alpha _{n-1}>\alpha _{n}$, Also, the process $\textbf{y}(\textsf{t})$ is the continuous time *q*-TASEP started from the random initial condition $\delta _{\textbf{x}(0)}\tilde{P}^{(n)}$ and evolves with the particle speeds $\sigma _{n-1}\varvec{\alpha }$.

#### Proposition 9.4

Fix $n\ge 1$. Given the trajectories of the particles $y_j(\textsf{t})$, $j=n-1,n,n+1$, in $\{\textbf{y}(\textsf{t})\}_{\textsf{t}\ge 0}$, replace the old trajectory $y_n(\textsf{t})$ by the new one $y_n'(\textsf{t})$ constructed from the process ${\tilde{H}}_n^{\rightarrow }$ started from $y_n'(0)=x_n(0)$. Then, the resulting trajectory of the whole process $\{y_1(\textsf{t}),\ldots ,y_{n-1}(\textsf{t}),y_n'(\textsf{t}),y_{n+1}(\textsf{t}),\ldots \}_{0\le \textsf{t}\le \textsf{M}}$ is equal in distribution with the trajectory of the process $\{\textbf{x}(\textsf{t})\}_{0\le \textsf{t}\le \textsf{M}}$.

#### Proof

This statement is also a continuous time limit of the general Theorem 7.9 and Corollary 7.11, as it was for Proposition 9.2. $\square$

#### Remark 9.5

*(TASEP specialization of* ${\tilde{H}}_n^{\rightarrow }$*)* The *q*-TASEP turns into the TASEP when $q=0$, and the dynamics ${\tilde{H}}_n^{\rightarrow }$ simplifies in that case. Namely, the blocking and pushing mechanisms stay the same, and the jump rates (9.14) become

9.16 $$\begin{aligned} \textrm{rate}^{\rightarrow }_{n;\alpha _{n-1},\alpha _n}\Big \vert _{q=0} =\alpha _{n-1}-\alpha _n\textbf{1}_{b=c}. \end{aligned}$$

Note that here the meaning of *b*, *c* differs from that in (9.13), as the process evolves forward in time $\textsf{t}$ instead of backwards in time. Thus, the process $y_n'(\textsf{t})$ is a Poisson random walk in the chamber $y_n(\textsf{t})\le y_n'(\textsf{t})< y_{n-1}(\textsf{t})$ running in forward in time and jumping up with rate $\alpha _{n-1}$ in the bulk and with rate $\alpha _{n-1}-\alpha _n$ at the bottom boundary of the chamber.

Clearly, Proposition 9.4 holds when $q=0$ for the process ${\tilde{H}}_n^{\rightarrow }$ with the jump rates (9.16). The case $n=1$ of this proposition with $q=0$ immediately implies Theorem 1.4 from the Introduction.

## Limit of Rewriting History Processes to Equal Particle Speeds

In this section, we obtain the limits of the rewriting history processes from Sect. 9 as the particle speeds $\alpha _i$ become equal. The corresponding rewriting history processes are powered by the independent bijectivisation for the intertwining relation from Theorem 5.6.

### Space of *q*-TASEP trajectories

Markov operators for rewriting history, as well as their limits which are continuous time Markov semigroups, act on the space of continuous time trajectories which we now describe.

#### Definition 10.1

Let $\mathscr {X}^{[0,\textsf{M}]}$ be the *space of trajectories of the continuous time q-TASEP* over time $0\le \textsf{t}\le \textsf{M}$. By definition, this space consists of trajectories $\mathfrak {x}=\{\textbf{x}(\textsf{t})\}_{0\le \textsf{t}\le \textsf{M}}$ satisfying the following conditions:

- For all $\textsf{t}$ we have $\textbf{x}(\textsf{t})\in \mathscr {X}$ (recall Definition 2.3).
- There exists *N* such that $x_n(\textsf{t})=-n$ for all $n>N$ and $0\le \textsf{t}\le \textsf{M}$.
- The trajectory of each particle $x_n(\textsf{t})$ is weakly increasing, piecewise constant, and makes increments of size 1.^5^
- At any time moment $0\le \textsf{t}\le \textsf{M}$, there is at most one such increment.

For each $\mathfrak {x}\in \mathscr {X}^{[0,\textsf{M}]}$, we associate the finite set $T_{\mathfrak {x}}\subset (0,\textsf{M})$ of all $\textsf{t}$ at which some particle has an increment. The space $\mathscr {X}^{[0,\textsf{M}]}$ has a natural topology in which $\mathfrak {x}$ and $\mathfrak {x}'$ are close iff $\textbf{x}(0)=\textbf{x}'(0)$, $\textbf{x}(\textsf{M})=\textbf{x}'(\textsf{M})$, all particles make all of their increments in the same order, and the increments’ times $T_{\mathfrak {x}},T_{\mathfrak {x}'}$ are close in the corresponding finite-dimensional space. In this topology, $\mathscr {X}^{[0,\textsf{M}]}$ has countably many connected components each of which may be identified with an open subset of $\mathbb {R}^d$ for a suitable *d*.

Markov operators $\tilde{\Xi }$ on $\mathscr {X}^{[0,\textsf{M}]}$ which we consider in the current Sect. 10 can map a trajectory $\mathfrak {x}$ to a random new trajectory $\mathfrak {x}'$ such that $T_{\mathfrak {x}'}=(T_{\mathfrak {x}}\setminus T^{-})\cup T^{+}$. That is, $\tilde{\Xi }$ removes a random subset $T^-\subset T_{\mathfrak {x}}$ of the existing increment times, and adds random new increment times belonging to $T^+$. The new increment times belong to continuous intervals, and are chosen randomly from probability densities with respect to the Lebesgue measure. Therefore, the operator $\tilde{\Xi }$ is determined by the transition densities

10.1 $$\begin{aligned} \frac{ \tilde{\Xi }(\mathfrak {x},\mathfrak {x}'+d\hspace{1pt}T^+)}{d\hspace{1pt}T^+}, \end{aligned}$$

which also incorporate the probabilities for the removed increment times.

### Slowdown operator for the *q*-TASEP

Assume now that the *q*-TASEP particle speeds are

10.2 $$\begin{aligned} \alpha _i=r^i,\qquad i\in \mathbb {Z}_{\ge 0}, \end{aligned}$$

with $0<r<1$. Taking $r\rightarrow 1$ leads to equal particle speeds. We consider this limit below in Sect. 10.3.

Recall from Sect. 5.3 that we denote the continuous time Markov semigroup of the *q*-TASEP with speeds (10.2) by $\{\tilde{T}_r^{\textrm{qT}}(\textsf{t})\}_{\textsf{t}\in \mathbb {R}_{\ge 0}}$, and the Markov shift operator for this process by $\tilde{B}_{r}^{\textrm{qT}}$. All these operators act on the space $\mathscr {X}$ of particle configurations. The (iterated) intertwining relation reads

10.3 $$\begin{aligned} \tilde{T}^{\textrm{qT}}_r(\textsf{t}) \bigl ( {\tilde{B}}^{\textrm{qT}}_{r} \bigr )^{m} = \bigl ( {\tilde{B}}^{\textrm{qT}}_{r} \bigr )^{m}\hspace{1pt}\tilde{T}^{\textrm{qT}}_r(r^{m}\hspace{1pt}\textsf{t}), \end{aligned}$$

where $\textsf{t}\in \mathbb {R}_{\ge 0}$ and $m\in \mathbb {Z}_{\ge 1}$ are arbitrary.

The action of ${\tilde{B}}_{r}^{\textrm{qT}}$ is, by definition, the sequential application of the Markov swap operators (denote them by ${\tilde{P}}^{(0,n)}_r$) over $n=1,2,\ldots$, see (4.6). Each swap operator ${\tilde{P}}^{(0,n)}_r$ acts by randomly moving the particle $x_n$ backwards and depends on the parameter $\alpha _n/\alpha _0=r^n$. Additionally, each swap operator ${\tilde{P}}^{(0,n)}_r$ gives rise to two rewriting history processes which we denote by $\tilde{H}_{\alpha _0,\alpha _n}^{\leftarrow } ={\tilde{H}}_{1,r^n}^{\leftarrow }$ and ${\tilde{H}}_{\alpha _0,\alpha _n}^{\rightarrow } = \tilde{H}_{1,r^n}^{\rightarrow }$, see Sect. 9.2, by the independent bijectivisation. Note that these processes depend not only on the ratio $r^n=\alpha _n/\alpha _0$ but, also, on both these parameters separately, see (9.10) and (9.14). In Sects. 10.2 and 10.3, we focus on the processes ${\tilde{H}}_{\alpha _0,\alpha _n}^{\leftarrow }$ of rewriting history from future to past, and consider the processes ${\tilde{H}}_{\alpha _0,\alpha _n}^{\rightarrow }$ below in Sect. 10.4.

Let us describe the slowdown Markov operators $\tilde{\Xi }_{m,r}^{\leftarrow }$ on the space $\mathscr {X}^{[0,\textsf{M}]}$ ((Definition 10.1).

#### Definition 10.2

Let $\mathfrak {x}=\{\textbf{x}(\textsf{t})\}_{0\le \textsf{t}\le \textsf{M}} \in \mathscr {X}^{[0,\textsf{M}]}$ and $m\in \mathbb {Z}_{\ge 1}$ be fixed. The action of $\tilde{\Xi }_{m,r}^{\leftarrow }$ on $\mathfrak {x}$ is as follows. First, apply the Markov shift operator ${\tilde{B}}^{\textrm{qT}}_r$ to $\textbf{x}(\textsf{M})$ and denote the resulting random configuration by $\textbf{x}'(\textsf{M})$. Then, apply the rewriting history Markov operator $\tilde{H}_{r^m,r^{m+n}}^{\leftarrow }$, sequentially for $n=1,2,\ldots$, to replace the old trajectory $\{x_n(\textsf{t})\}_{0\le \textsf{t}\le \textsf{M}}$ by the random new trajectory $\{x_n'(\textsf{t})\}_{0\le \textsf{t}\le \textsf{M}}$ given the following data:

$$\begin{aligned} \{x_{n-1}'(\textsf{t})\}_{0\le \textsf{t}\le \textsf{M}}, \qquad x_n'(\textsf{M}), \qquad \{x_{n+1}(\textsf{t})\}_{0\le \textsf{t}\le \textsf{M}} \end{aligned}$$

with $x_{n-1}'(\textsf{t})=+\infty$ for $n=1$, by agreement. The updates, for $n=1,2,\ldots$, eventually terminate since $x_n(t) = -n$ for $n >N$ if *N* is large enough (recall that this is the property of $\mathscr {X}^{[0,\textsf{M}]}$). The new random trajectory $\mathfrak {x}'=\{\textbf{x}'(\textsf{t})\}_{0\le \textsf{t}\le \textsf{M}}$ is, by definition, the result of applying the Markov operator $\tilde{\Xi }_{m,r}^{\leftarrow }$ to $\mathfrak {x}$.

Let us make two comments regarding Definition 10.2. First, the new initial configuration $\textbf{x}'(0)$ in $\mathfrak {x}'$ is random and, for $m=1$, it is distributed as $\delta _{\textbf{x}(0)}{\tilde{B}}^{\textrm{qT}}_r$, due to the intertwining relation. Second, there is an important difference between ${\tilde{H}}_{r^{m},r^{m+n}}^{\leftarrow }$ and $\tilde{\Xi }_{m,r}^{\leftarrow }$. The former assumes that the new terminal configuration $x_n'(\textsf{M})$ is fixed and, in the latter, the terminal configuration evolves randomly. We use different letters for these operators because of this.

We now describe the action of the operators $\tilde{\Xi }_{i,r}^{\leftarrow }$ on the *q*-TASEP measures on trajectories. Let $\mathfrak {x}=\{\textbf{x}(\textsf{t})\}_{0\le \textsf{t}\le \textsf{M}}$ be the trajectory of the continuous time *q*-TASEP with particle speeds (10.2) started from a fixed initial configuration $\textbf{x}(0)$. Fix $m\in \mathbb {Z}_{\ge 0}$, and let $\mathfrak {y}=\{\textbf{y}(\textsf{t})\}_{0\le \textsf{t}\le \textsf{M}}$ be the continuous time *q*-TASEP with the same speeds (10.2) but, instead, with random initial configuration $\delta _{\textbf{x}(0)}\bigl (\tilde{B}^{\textrm{qT}}_r\bigr )^m$.

#### Proposition 10.3

Given the above notation, apply the Markov operators $\tilde{\Xi }_{0,r}^{\leftarrow }, \tilde{\Xi }_{1,r}^{\leftarrow }, \ldots , \tilde{\Xi }_{m-1,r}^{\leftarrow }$, in this order, to the *q*-TASEP trajectory $\mathfrak {x}$. Then, the resulting trajectory has the same distribution as $\{\textbf{y}(r^m\textsf{t})\}_{0\le \textsf{t}\le \textsf{M}}$.

Note that the operators $\tilde{\Xi }_{i,r}^{\leftarrow }$ “slow down” the time evolution of *q*-TASEP by shrinking the time variable. We call $\tilde{\Xi }_{i,r}^{\leftarrow }$ the *slowdown operators* on trajectories because of this.

#### Proof of Proposition 10.3

The result follows by iterating Proposition 9.2 over all *n* and, then, repeating *m* times. The result, after the application of the composition of the operators $\tilde{\Xi }_{0,r}^{\leftarrow } \tilde{\Xi }_{1,r}^{\leftarrow } \ldots \tilde{\Xi }_{m-1,r}^{\leftarrow }$, is the *q*-TASEP with speeds $(r^m,r^{m+1},\ldots )$ and initial configuration $\delta _{\textbf{x}(0)}\bigl ({\tilde{B}}^{\textrm{qT}}_r\bigr )^m$. Additionally, note that multiplying all speeds by $r^m$ is the same as slowing down the time by the overall factor $r^m$ since our *q*-TASEP runs in continuous time. multiplying all speeds by $r^m$ is the same as slowing down the time by the overall factor $r^m$. Moreover, this does not affect the random initial configuration $\textbf{y}(0)$. This completes the proof.

### Slowdown dynamics for the homogeneous *q*-TASEP

Let us now take the limit as in Sect. 5.4,

10.4 $$\begin{aligned} r\nearrow 1,\qquad m=\lfloor (1-r)^{-1}\tau \rfloor , \end{aligned}$$

where $\tau \in \mathbb {R}_{\ge 0}$ is the continuous time parameter. In this limit, the *q*-TASEP becomes *homogeneous* with particle speeds $\alpha _i=1$ for all *i*, and the operators $\bigl ({\tilde{B}}^{\textrm{qT}}_r\bigr )^m$ turn into the continuous time Markov semigroup $\{{\tilde{B}}^{\textrm{qT}}(\tau )\}_{\tau \in \mathbb {R}_{\ge 0}}$ on $\mathscr {X}$. This semigroup corresponds to the backwards *q*-TASEP dynamics [Pet21]; we recalled the definition in Sect. 5.4. Our aim now is to extend the semigroup $B^{\textrm{qT}}(\tau )$ on $\mathscr {X}$ to continuous time Markov dynamics on the space $\mathscr {X}^{[0,\textsf{M}]}$ of trajectories by taking the limit (10.4) of the slowdown operators. The latter dynamics are denoted by $\tilde{\Xi }^{\leftarrow }(\tau )$. An interesting feature of $\tilde{\Xi }^{\leftarrow }(\tau )$, compared to $B^{\textrm{qT}}(\tau )$, is that, while the former is a *time-inhomogeneous Markov process* (its transitions depend on the time variable), the latter is time-homogeneous. See Remark 10.6 below for more discussion.

Let us first define the dynamics $\tilde{\Xi }^{\leftarrow }(\tau )$. Then, in Proposition 10.5 below, we show that these dynamics are the desired $r\rightarrow 1$ limit of the sequential application of the operators $\tilde{\Xi }_{i,r}^{\leftarrow }$.

#### Definition 10.4

Fix $\textsf{M}\in \mathbb {R}_{\ge 0}$. The continuous time *slowdown Markov dynamics* $\tilde{\Xi }^{\leftarrow }(\tau )$ acting on trajectories $\mathfrak {x}=\{\textbf{x}(\textsf{t})\}_{0\le \textsf{t}\le \textsf{M}} \in \mathscr {X}^{[0,\textsf{M}]}$ possesses two sources of independent jumps and, also, a random jump propagation mechanism. Almost surely there are only finitely many independent jumps in finite time. The independent jumps are as follows:

- (*terminal jumps*) The terminal configuration $\textbf{x}(\textsf{M})$ in $\mathfrak {x}$ evolves according to the backwards *q*-TASEP ${\tilde{B}}^{\textrm{qT}}(\tau )$. Recall, from Sect. 5.4, that this means that each particle $x_n(\textsf{M})$, $n=1,2,\ldots$, jumps down to a new location $x_n'(\textsf{M})$, where $x_{n+1}(\textsf{M})<x_n'(\textsf{M})<x_n(\textsf{M})$, with rate
  $$\begin{aligned} \frac{n\hspace{1pt}(q;q)_{x_n'(\textsf{M})-x_{n+1}(\textsf{M})-1}}{(1-q^{x_n(\textsf{M})-x_n'(\textsf{M})}) \hspace{1pt}(q;q)_{x_n(\textsf{M})-x_{n+1}(\textsf{M})-1}}. \end{aligned}$$
- (*bulk jumps*) For all $0<\textsf{t}<\textsf{M}$, each particle $x_n(\textsf{t})$ can jump down by 1 according to the following mechanism. Let
  $$\begin{aligned} T_{\mathfrak {x}}= \{ 0=\textsf{t}_0<\textsf{t}_1<\textsf{t}_2<\ldots<\textsf{t}_k<\textsf{t}_{k+1}=\textsf{M}\}. \end{aligned}$$
  For $n\ge 1$ and $1\le j\le k+1$, attach to each segment $[x_n(\textsf{t}_{j-1}),x_n(\textsf{t}_{j})]$ an independent exponential clock of (time-inhomogeneous) rate
  10.5 $$\begin{aligned} \left( x_n(\textsf{t}_j)-x_n(\textsf{t}_{j-1}) \right) \frac{n \hspace{1pt}e^{-\tau }\hspace{1pt}(1-q^{x_{n-1}(\textsf{t})-x_n(\textsf{t})})(1-q^{x_n(\textsf{t})-x_{n+1}(\textsf{t})-1})}{1-q}, \qquad \text {where }\,\, \textsf{t}\in (\textsf{t}_{j-1},\textsf{t}_j). \nonumber \\ \end{aligned}$$
  Here $\tau$ is the time variable in the dynamics $\tilde{\Xi }^{\leftarrow }(\tau )$. When the clock rings, place a uniformly random point $\textsf{t}_*\in (\textsf{t}_{j-1},\textsf{t}_j)$. Then, let the trajectory of $x_n$ make a new increment at $\textsf{t}_*$, so that $x_n'(\textsf{t}_*)=x_n(\textsf{t}_*)-1$ and $x_n'(\textsf{t}_*+)=x_n(\textsf{t}_*+)$. Note that if $x_n(\textsf{t}_*)=x_{n+1}(\textsf{t}_*)+1$, the particle $x_n(\textsf{t}_*)$ is blocked from jumping down, and the rate (10.5) vanishes, as it should.

Let us now describe the *jump propagation mechanism*. Assume that a particle $x_n(\textsf{t}_*)$, $\textsf{t}_*\in (0,\textsf{M}]$, has jumped down to $x_n'(\textsf{t}_*)$ as described above. We either have a terminal jump with $\textsf{t}_*=\textsf{M}$ or a bulk jump with a random $\textsf{t}_*<\textsf{M}$. The jump then instantaneously propagates left according to a, backwards in time, random walk in the chamber $x_{n}(\textsf{t})\ge x_{n}'(\textsf{t})>x_{n+1}(\textsf{t})$, $0\le \textsf{t}\le \textsf{t}_*$. This random walk makes jumps down by 1 in continuous time $\textsf{t}$ with rate

10.6 $$\begin{aligned} \frac{e^{-\tau }\hspace{1pt}(1- q^{b-c})(1-q^{a+b+1-c})(1-q^c)}{1-q^{b-c+1}}, \end{aligned}$$

where *a*, *b*, *c* are given in (9.11). Note that (10.6) vanishes for $b=c$. This means that, during the instantaneous jump propagation, $x_n'(\textsf{t})$ either joins the old trajectory of $x_n$ and continues to follow it until $\textsf{t}=0$, or $x_n'(\textsf{t})$ modifies the initial configuration $\textbf{x}(0)$. In particular, the new trajectory will not deviate from the old trajectory once it has deviated and joined the old trajectory. The trajectories of all other particles $x_l$, $l\ne n$, do not change during this instantaneous jump propagation.

**Fig. 23:**
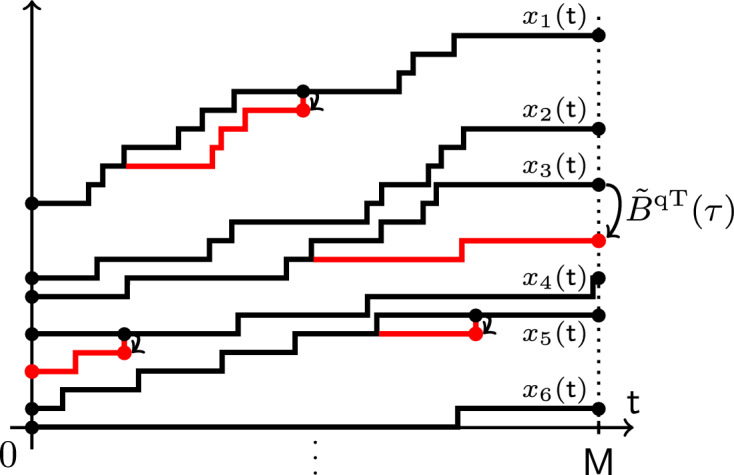
The slowdown dynamics $\tilde{\Xi }^{\leftarrow }(\tau )$ acting on trajectories. In the figure there are four possible transitions: one initiated at $\textsf{t}=\textsf{M}$ by the backwards *q*-TASEP ${\tilde{B}}^{\textrm{qT}}$, and three others initiated by bulk independent jumps. Note that one of the jump propagations modifies the initial configuration $\textbf{x}(0)$

We refer to Fig. 23 for an illustration of the dynamics $\tilde{\Xi }^{\leftarrow }(\tau )$.

#### Proposition 10.5

Fix $\tau \in \mathbb {R}_{\ge 0}$. Then, the limit

$$\begin{aligned} \lim \nolimits _{r\nearrow 1} \tilde{\Xi }_{0,r}^{\leftarrow } \tilde{\Xi }_{1,r}^{\leftarrow } \ldots \tilde{\Xi }_{m-1,r}^{\leftarrow } = \tilde{\Xi }^{\leftarrow }(\tau ), \qquad \text {with}\,\, m=\lfloor (1-r)^{-1}\tau \rfloor , \end{aligned}$$

converges in the sense of the transition densities (10.1) associated to Markov operators on $\mathscr {X}^{[0,\textsf{M}]}$.

#### Proof

Let $m=\lfloor (1-r)^{-1}\tau \rfloor$, and consider the action of the slowdown operator $\tilde{\Xi }_{m,r}^{\leftarrow }$ on the *n*-th particle. By Theorem 5.6, the action of the terminal jumps by ${\tilde{B}}^{\textrm{qT}}(\tau )$ follows. In particular, we note that the events of a terminal jump has probability of order $O(1-r)$ as $r \rightarrow 1$. It remains to consider bulk jumps and jump propagation.

Recall that ${\tilde{H}}_{r^{m},r^{m+n}}^{\leftarrow }$ replaces the old trajectory $x_n(\textsf{t})$ by a random walk $x_n'(\textsf{t})$ in reverse continuous time from $\textsf{M}$ to 0 which makes steps down by 1 at rates $\textrm{rate}^{\leftarrow }_{n;r^m,r^{m+n}}$, see (9.10). If $b=c$, the rate $\textrm{rate}^{\leftarrow }_{n;r^m,r^{m+n}}$ is of order $O(1-r)$ as $r \rightarrow 1$. Otherwise, if $b>c$, the rate $\textrm{rate}^{\leftarrow }_{n;r^m,r^{m+n}}$ is of order *O*(1) as $r \rightarrow 1$.

Note that only one event with probability $O(1-r)$ may happen in a single moment of the new continuous time $\tau$ in the Poisson-type limit for $\tilde{\Xi }_{0,r}^{\leftarrow } \tilde{\Xi }_{1,r}^{\leftarrow } \ldots \tilde{\Xi }_{m-1,r}^{\leftarrow }$ as $r\rightarrow 1$. It is either a terminal jump or a bulk jump. Recall that a terminal jump happens according to ${\tilde{B}}^{\textrm{qT}}(\tau )$.

For bulk jumps, observe that

$$\begin{aligned} \textrm{rate}^{\leftarrow }_{n;r^m,r^{m+n}}= \frac{n \hspace{1pt}e^{-\tau }\hspace{1pt}(1-q^{a+1})(1-q^{c})}{1-q} \hspace{1pt}(1-r)+O(1-r)^2, \end{aligned}$$

where *a*, *c* are given in (9.11). Therefore, during the continuous time $d\tau$, on a segment of length $d\textsf{t}$ there is a new independent bulk jump with small probability

10.7 $$\begin{aligned} \frac{n \hspace{1pt}e^{-\tau }\hspace{1pt}(1-q^{x_{n-1}(\textsf{t})-x_n(\textsf{t})})(1-q^{x_n(\textsf{t})-x_{n+1}(\textsf{t})-1})}{1-q}\hspace{1pt}d\tau \hspace{1pt}d\textsf{t}. \end{aligned}$$

Moreover, a bulk jump can happen on at most one interval. Averaging (10.7) over the segment $[x_n(\textsf{t}_{j-1}),x_n(\textsf{t}_{j})]$ where this jump happens immediately leads to the desired jump rates (10.5).

Finally, once an event with probability $O(1-r)$ occurred for a particle $x_n$ at $\textsf{t}=\textsf{t}_*$, the rest of the $x_n$’s trajectory to the left of $\textsf{t}_*$ needs to be instantaneously modified. This modification happens according to the random walk ${\tilde{H}}_{r^m,r^{m+n}}^{\leftarrow }$. This leads to further nontrivial jumps down by 1 when $b>c$. In the case that the new trajectory joins the old trajectory after a terminal or bulk jump, we will have $b=c$ and the probability that the new trajectory jumps down by one again is of order $O(1-r)$, with an overall probability of $O((1-r)^2)$ for this sequence of events. Thus, the new trajectory will not deviate from the old trajectory after deviating once and joining the old trajectory. Then, in the limit as $r\rightarrow 1$, this jump propagation turns into the Poisson random walk with rates (10.6). This completes the proof. $\square$

#### Remark 10.6

Notice that the time inhomogeneity in $\tilde{\Xi }^{\leftarrow }(\tau )$ is only present in the bulk jumps and jump propagation, but not in the terminal jumps. From the proof of Proposition 10.5, this is because the rates of bulk jumps for $x_n$ depend on both parameters $\alpha _m=r^m$, $\alpha _{m+n}=r^{m+n}$ before the $r\rightarrow 1$ limit, while for the terminal jumps they only depend on the ratio $\alpha _{m+n}/\alpha _m=\alpha _n/\alpha _0=r^n$.

From Proposition 10.5 and 10.3 we immediately get the following slowdown action of the dynamics $\tilde{\Xi }^{\leftarrow }(\tau )$ on trajectories of the homogeneous continuous time *q*-TASEP with all particles speeds equal to one:

#### Proposition 10.7

Fix $\tau \in \mathbb {R}_{\ge 0}$, and let $\mathfrak {x}=\{\textbf{x}(\textsf{t})\}_{0\le \textsf{t}\le \textsf{M}}$ and $\{\textbf{y}(\textsf{t})\}_{0\le \textsf{t}\le \textsf{M}}$ be the homogeneous continuous time *q*-TASEPs started from a fixed initial configuration $\textbf{x}(0)$ and from the random initial configuration $\delta _{\textbf{x}(0)}\tilde{B}^{\textrm{qT}}(\tau )$, respectively. Apply $\tilde{\Xi }^{\leftarrow }(\tau )$ to $\mathfrak {x}$, that is, run the slowdown dynamics (Definition 10.4) for time $\tau$ started from $\mathfrak {x}$. Then, the resulting random trajectory is distributed as $\{\textbf{y}(e^{-\tau }\textsf{t})\}_{0\le \textsf{t}\le \textsf{M}}$.

### Speedup dynamics for the *q*-TASEP with step initial configuration

The slowdown process $\tilde{\Xi }^{\leftarrow }(\tau )$ constructed above in Sect. 10.3 provides a bijectivisation of the intertwining relation of Theorem 5.6,

10.8 $$\begin{aligned} \tilde{T}^{\textrm{qT}}(\textsf{t}) \hspace{1pt}{\tilde{B}}^{\textrm{qT}}(\tau ) = {\tilde{B}}^{\textrm{qT}}(\tau ) \hspace{1pt}\tilde{T}^{\textrm{qT}}\bigl (e^{-\tau }\textsf{t}\bigr ). \end{aligned}$$

One can informally say that the slowdown process acts on identity (10.8) from the left-hand side to the right-hand side, see Proposition 10.7. The slowdown process contains, in particular, the backwards *q*-TASEP ${\tilde{B}}^{\textrm{qT}}(\tau )$ running on the terminal configuration $\textbf{x}(\textsf{M})$ of the trajectory. In this subsection we discuss a bijectivisation of (10.8) in another direction, from right to left, by means of a *speedup process* $\tilde{\Xi }^{\rightarrow }(\tau )$. To simplify notation and formulations, we only consider the action of the speedup process on trajectories with the step initial configuration

$$\begin{aligned} \textbf{y}(0)=\textbf{x}_{step}=\{\ldots ,-3,-2,-1 \}. \end{aligned}$$

#### Remark 10.8

In the general case, the initial configuration $\textbf{y}(0)$ must be random with distribution $\delta _{\textbf{x}(0)}\hspace{1pt}\tilde{B}^{\textrm{qT}}(\tau )$ where $\textbf{x}(0)\in \mathscr {X}$ is fixed. In particular, the speedup process itself would need as its input a deterministic sequence of down particle jumps at the trajectory’s initial configuration. That is, such a sequence would record the transition from $\textbf{x}(0)$ to $\textbf{y}(0)$ during time $\tau$. These deterministic jumps can be easily incorporated into the definitions and the results given below in the current Sect. 10.4, similarly to the second part of Theorem 7.9 and Proposition 9.4. However, for simplicity, we omit the discussion of this more general case and focus only on trajectories with the step initial configuration.

The Markov operator $\tilde{\Xi }^{\rightarrow }(\tau )$ of the speedup process, on trajectories with the step initial configuration, is constructed as the $r\nearrow 1$ limit of the sequential application of the corresponding speedup operators $\tilde{\Xi }_{0,r}^{\rightarrow } \tilde{\Xi }_{1,r}^{\rightarrow } \ldots \tilde{\Xi }_{m-1,r}^{\rightarrow }$, where $m=\lfloor (1-r)^{-1}\tau \rfloor$. In contrast with the slowdown operators (Definition 10.2), the action of $\tilde{\Xi }_{i,r}^{\rightarrow }$ is a sequential application of the history rewriting processes ${\tilde{H}}_{r^{-i}; r^{-i+n}}^{\rightarrow }$, where *n* decreases from a suitably large *N* down to 1. Here, *N* depends on the trajectory $\mathfrak {y}=\{\textbf{y}(\textsf{t})\}_{0\le \textsf{t}\le \textsf{M}} \in \mathscr {X}^{[0,\textsf{M}]}$ to which $\tilde{\Xi }_{i,r}^{\rightarrow }$ is applied. More precisely, *N* is determined so that $y_k(\textsf{t})=-k$ for all $\textsf{t}\in [0,\textsf{M}]$ and $k\ge N-1$, see Definition 10.1. Similarly to Proposition 10.3, one can check that the action of $\tilde{\Xi }_{0,r}^{\rightarrow } \tilde{\Xi }_{1,r}^{\rightarrow } \ldots \tilde{\Xi }_{m-1,r}^{\rightarrow }$ on a trajectory $\mathfrak {y}=\{\textbf{y}(\textsf{t})\}_{\textsf{t}\ge 0}$ of the *q*-TASEP with particle speeds $(1,r,r^2,\ldots )$ results in a trajectory of the *q*-TASEP with particle speeds $(r^{-m},r^{-m+1},r^{-m+2},\ldots )$; the initial configuration is $\textbf{x}_{step}$ in both processes. Equivalently, one can say that the action of the Markov operator $\tilde{\Xi }_{0,r}^{\rightarrow } \tilde{\Xi }_{1,r}^{\rightarrow } \ldots \tilde{\Xi }_{m-1,r}^{\rightarrow }$ speeds up the time $\textsf{t}$ in the *q*-TASEP with rates $(1,r,r^2,\ldots )$ by the factor $r^{-m}>1$.

The $r\nearrow 1$ limit of the operators $\tilde{\Xi }_{0,r}^{\rightarrow } \tilde{\Xi }_{1,r}^{\rightarrow } \ldots \tilde{\Xi }_{m-1,r}^{\rightarrow }$ in the sense of the transition densities (10.1) is obtained very similarly to Sect. 10.3, with an additional simplification coming from the step initial configuration. Therefore, here we will only define the resulting continuous time speedup process $\tilde{\Xi }^{\rightarrow }(\tau )$, and formulate an analogue of Sect. 10.7.

#### Definition 10.9

Fix $\textsf{M}\in \mathbb {R}_{\ge 0}$. The continuous time *speedup Markov dynamics* $\tilde{\Xi }^{\rightarrow }(\tau )$ acting on trajectories $\mathfrak {y}=\{\textbf{y}(\textsf{t})\} _{0\le \textsf{t}\le \textsf{M}} \in \mathscr {X}^{[0,\textsf{M}]}$ with $\textbf{y}(0)=\textbf{x}_{step}$ possesses one source of independent jumps (the *bulk jumps*), and a mechanism of random jump propagation. Let

$$\begin{aligned} T_{\mathfrak {y}}=\{0=\textsf{t}_0<\textsf{t}_1<\ldots<\textsf{t}_k <\textsf{t}_{k+1}=\textsf{M}\}. \end{aligned}$$

For $n\ge 1$ and $1\le j\le k+1$, attach to each segment $[y_n(\textsf{t}_{j-1}),y_n(\textsf{t}_j)]$ an independent exponential clock of (time-inhomogeneous) rate

10.9 $$\begin{aligned} &  \left( y_n(\textsf{t}_j)-y_n(\textsf{t}_{j-1}) \right) \frac{n\hspace{1pt}e^{\tau } (1-q^{y_n(\textsf{t})-y_{n+1}(t)}) (1-q^{y_{n-1}(\textsf{t})-y_{n}(\textsf{t})-1})}{1-q},\nonumber \\ &  \qquad \text {where }\,\, \textsf{t}\in (\textsf{t}_{j-1},\textsf{t}_{j}). \end{aligned}$$

Here, $\tau$ is the time variable in the dynamics $\tilde{\Xi }^{\rightarrow }(\tau )$. When the clock rings, place a uniformly random point $\textsf{t}_*\in (\textsf{t}_{j-1},\textsf{t}_j)$, and let the trajectory of $y_n$ make a new increment at $\textsf{t}_*$. In particular, $y_n'(\textsf{t}_*)=y_n(\textsf{t}_*)+1$ and $y_n'(\textsf{t}_*-)=y_n(\textsf{t}_*-)$. Note that if $y_n(\textsf{t}_*)=y_{n-1}(\textsf{t}_*)-1$, the particle $y_n(\textsf{t}_*)$ is blocked from jumping up, and the rate (10.9) vanishes, as it should. Almost surely there are only finitely many independent jumps in finite time.

Let us now describe the *jump propagation*. If a particle $y_n(\textsf{t}_*)$ has jumped up to $y_n'(\textsf{t}_*)$, then the new trajectory of $y_n'$ coincides with that of $y_n$ for $\textsf{t}<\textsf{t}_*$. To the right of $\textsf{t}_*$, instantaneously (in $\tau$) continue the new trajectory $y_n'$ according to a Poisson simple random walk in forward time $\textsf{t}$ in the chamber $y_n(\textsf{t})\le y_n'(\textsf{t})< y_{n-1}(\textsf{t})$ which makes jumps up by 1 in continuous time $\textsf{t}$ with rate

10.10 $$\begin{aligned} \frac{e^{\tau }\hspace{1pt}(1-q^{b-c})(1-q^a)(1-q^{b+1})}{1-q^{b-c+1}}, \end{aligned}$$

where *a*, *b*, *c* are given in (9.15). During this instantaneous jump propagation, $y_n'(\textsf{t})$ either joins the old trajectory of $y_n$ and continues to follow it till $\textsf{t}=\textsf{M}$, or modifies the terminal configuration $\textbf{y}(\textsf{M})$. In particular, the new trajectory will not deviate from the old trajectory once it has deviated and joined the old trajectory. The trajectories of all other particles $y_l$, $l\ne n$, are no affected by this instantaneous jump propagation.

The jump rates (10.9) and (10.10) in the speedup process $\tilde{\Xi }^{\rightarrow }(\tau )$ are obtained in the $r\rightarrow 1$ expansion of the jump rates (9.14) (with $\alpha _{n-1}=r^{-m}$, $\alpha _n=r^{-m+n}$) for $b=c$ and $b>c$, respectively. The argument here is very similar to the proof of Proposition 10.5.

The speedup process acts on trajectories of the homogeneous *q*-TASEP with the step initial configuration as follows:

#### Proposition 10.10

Let $\mathfrak {y}=\{\textbf{y}(\textsf{t})\}_{\textsf{t}\ge 0}$ be the homogeneous continuous time *q*-TASEP started from $\textbf{x}_{step}$. Let $\tau ,\textsf{M}\in \mathbb {R}_{\ge 0}$. Apply the speedup operator $\tilde{\Xi }^{\rightarrow }(\tau )$ to $\mathfrak {y}$ on $[0,\textsf{M}]$. Then, the resulting random trajectory is distrubuted as $\{\textbf{y}(e^{\tau }\textsf{t})\}_{0\le \textsf{t}\le \textsf{M}}$.

### Slowdown and speedup dynamics for the homogeneous TASEP

The slowdown and speedup processes $\tilde{\Xi }^{\leftarrow }(\tau )$ and $\tilde{\Xi }^{\rightarrow }(\tau )$ for the homogeneous TASEP, with particle speeds $\alpha _i=1$ for all *i*, are obtained by setting $q=0$ in the processes from Definitions 10.4 and 10.9, respectively. This greatly simplifies the dynamics. In this subsection we provide the necessary definitions.

We start with the slowdown process $\tilde{\Xi }^{\leftarrow }(\tau )$ acting on the space of trajectories $\mathscr {X}^{[0,\textsf{M}]}$ (Definition 10.1), where $\textsf{M}\in \mathbb {R}_{>0}$ is fixed:

#### Definition 10.11

The continuous time slowdown Markov process $\{\tilde{\Xi }^{\leftarrow }(\tau )\}_{0\le \tau \le \tau _0}$ for TASEP acts on a trajectory $\mathfrak {x}=\{\textbf{x}(\textsf{t}\mathsf )\}_{0\le \textsf{t}\le \textsf{M}} \in \mathscr {X}^{[0,\textsf{M}]}$ as follows:

- (*terminal jumps*) Run the backwards Hammersley dynamics at the terminal configuration $\textbf{x}(\textsf{M})$. In particular, each particle $\textsf{x}_n(\textsf{M})$, $n=1,2,\ldots$, jumps down to a new location $\textsf{x}_n'(\textsf{M})$, with $x_{n+1}(\textsf{M})<x_n'(\textsf{M})<x_n(\textsf{M})$ and rate *n* per available location to land. This process is time-homogeneous in $\tau$, and it is called the backwards Hammersley process.
- (*bulk jumps*) Take an independent two-dimensional Poisson process $\mathfrak {P}_{[n]}$ in $[0,\textsf{M}]\times [0,\tau _0]$, for each $n\ge 1$, with inhomogeneous rate $e^{-\tau }$. As $\tau$ increases from 0 to $\tau _0$, each point $(\textsf{t}_*,\tau _*)$ of $\mathfrak {P}_{[n]}$ generates a jump down by 1 of the trajectory $\{x_n(\textsf{t})\}_{0\le \textsf{t}\le \textsf{M}}$ at $\textsf{t}=\textsf{t}_*$. This means that we set $x_n'(\textsf{t}_*)=x_n(\textsf{t}_*)-1$ and $x_n'(\textsf{t}_*+)=x_n(\textsf{t}_*+)$ if $x_n(\textsf{t}_*)>x_{n+1}(\textsf{t}_*)+1$. Otherwise, if $x_n(\textsf{t}_*)=x_{n+1}(\textsf{t}_*)+1$, the jump down is blocked, and the trajectory $\mathfrak {x}$ is not changed.
- (*jump propagation*) Replace, instantaneously at the same moment $\tau =\tau _*$, the old trajectory of $x_n$ by the new one, $x_n'$, for all $\textsf{t}\le \textsf{t}_*$, where $\textsf{t}_*\le \textsf{M}$ is the time a terminal or a bulk jump which occurred. The new trajectory $x_n'$ starts from $x_n'(\textsf{t}_*)$ and evolves in backwards time $\textsf{t}$ in the chamber $x_{n}(\textsf{t})\ge x_{n}'(\textsf{t})>x_{n+1}(\textsf{t})$, $0\le \textsf{t}\le \textsf{t}_*$. The dynamics of $x_n'(\textsf{t})$ is that of the Poisson simple random walk which makes jumps down at rate $e^{-\tau _*}$, and gets absorbed by the top wall $x_n(\textsf{t})$ of the chamber once it reaches it. In other words, $x_n'(\textsf{t})$ either joins the old trajectory of $x_n$ and continues to follow it till $\textsf{t}=0$, or it modifies the initial configuration $\textbf{x}(0)$. The trajectories of all other particles $x_l$, $l\ne n$, do not change during this instantaneous jump propagation.

The slowdown process $\tilde{\Xi }^{\leftarrow }(\tau )$ satisfies the $q=0$ version of Proposition 10.7, that is, where the *q*-TASEP is replaced by the TASEP.

Let us now describe the speedup process for TASEP. As in Sect. 10.4, we restrict attention only to trajectories $\mathfrak {y}=\{\textbf{y}(\textsf{t})\}_{0\le \textsf{t}\le \textsf{M}} \in \mathscr {X}^{[0,\textsf{M}]}$ with the step initial configuration $\textbf{y}(0)=\textbf{x}_{step}$ (see Remark 10.8 for more discussion). The $q=0$ speedup process is given as follows:

#### Definition 10.12

The continuous time process $\tilde{\Xi }^{\rightarrow }(\tau )$ for TASEP acts on trajectories $\mathfrak {y}=\{\textbf{y}(\textsf{t})\}_{0\le \textsf{t}\le \textsf{M}}$ with the step initial configuration as follows:

- (*bulk jumps*) Take an independent two-dimensional Poisson process $\mathfrak {P}_{[n]}$ in $[0,\textsf{M}]\times [0,\tau _0]$, for each $n\ge 1$, of inhomogeneous rate $e^{\tau }$. As $\tau$ increases from 0 to $\tau _0$, each point $(\textsf{t}_*,\tau _*)$ of $\mathfrak {P}_{[n]}$ generates a jump up by 1 of the trajectory $\{t_n(\textsf{t})\}_{0\le \textsf{t}\le \textsf{M}}$ at $\textsf{t}=\textsf{t}_*$. This means that $y_n'(\textsf{t}_*)=y_n(\textsf{t}_*)+1$ and $y_n'(\textsf{t}_*-)=y_n(\textsf{t}_*-)$ unless this jump is blocked, when $y_n(\textsf{t}_*)=y_{n-1}(\textsf{t}_*)-1$). In the case of blocking, the trajectory of $y_n$ is not changed.
- (*jump propagation*) Replace, instantaneously at the same moment $\tau =\tau _*$, the trajectory of $y_n$ with a new trajectory $y_n'$. If a particle $y_n(\textsf{t}_*)$ has jumped up by 1 to $y_n'(\textsf{t}_*)$, then the new trajectory of $y_n'$ coincides with that of $y_n$ for $\textsf{t}<\textsf{t}_*$. To the right of $\textsf{t}_*$, continue the new trajectory $y_n'$ according to a Poisson simple random walk in forward time $\textsf{t}$ in the chamber $y_n(\textsf{t})\le y_n'(\textsf{t})< y_{n-1}(\textsf{t})$. The random walk makes a jump up by 1 in continuous time $\textsf{t}$ at rate $e^{\tau _*}$ and gets absorbed by the bottom wall $y_n(t)$ of the chamber once it reaches it. In particular, $y_n'$ either joins the old trajectory of $y_n$ and continues to follow it till $\textsf{t}=\textsf{M}$, or it modifies the terminal configuration $\textbf{y}(\textsf{M})$. The trajectories of all other particles $y_l$, $l\ne n$, are no affected by this instantaneous jump propagation.

The process $\tilde{\Xi }^{\rightarrow }(\tau )$ satisfies the $q=0$ version of Proposition 10.10, that is, the same statement where the word “*q*-TASEP” is replaced by “TASEP”.

The action of the speedup dynamics on the first particle produces an interesting coupling between standard Poisson processes on the positive half-line with different slopes (here by “slope” we mean the slope of the counting function of the Poisson process, which is usually referred to as “rate”, “intensity”, or “density”). First, observe that the trajectory $\{y_1(\textsf{t})\}_{\textsf{t}\ge 0}$ of the first TASEP particle is the continuous time Poisson simple random walk with slope 1. By Proposition 10.10 for $q=0$, after running the speedup process $\tilde{\Xi }^{\rightarrow }$ for time $\tau >0$, the resulting trajectory $\{y_1'(\textsf{t})\}_{\textsf{t} \ge 0}$ of the first particle is the continuous time Poisson simple random walk with slope $e^\tau$. Moreover, note that the trajectory of the first particle evolves *independently* from all other particles, under the speedup dynamics. This independence is a result of setting $q=0$, since for $q>0$ the jump rates in $\tilde{\Xi }^{\rightarrow }$ depend on the second trajectory $y_2(\textsf{t})$.

Let us now describe the evolution of the trajectory for the first particle. We may assume that $\textsf{M}=+\infty$ and give the description for the full trajectories, that is, where $\textsf{t}\in [0,+\infty )$. Change the time of the speedup process as $\hat{\tau }=e^{\tau }-1$. Then, the two-dimensional Poisson process $\mathfrak {P}_{[1]}$ of rate $e^\tau$, with $0\le \tau \le \tau _0$, turns into the homogeneous two-dimensional Poisson process $\hat{\mathfrak {P}}_{[1]}$ of rate 1, with $0\le \tau \le \hat{\tau }_0=e^{\tau _0}-1$.

**Fig. 24:**
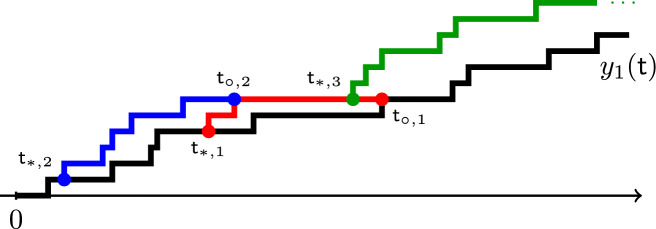
Three consecutive jumps in the process $\tilde{\Xi }^{\rightarrow }$ acting on the Poisson simple random walk $y_1(\textsf{t})$. Each *i*-th jump originates from $\textsf{t}_{*,i}$, which are the horizontal coordinates of the points of a two-dimensional Poisson process $\hat{\mathfrak {P}}_{[1]}$, and terminates at $\textsf{t}_{\circ ,i}\in [0,+\infty ]$. One can say that the trajectory grows by instantaneous “avalanches”

For each point $(\textsf{t}_*,\hat{\tau }_*)$ of $\hat{\mathfrak {P}}_{[1]}$, set $y_1'(\textsf{t}_*)=y_1(\textsf{t}_*)+1$. Then, instantaneously replace a piece of the trajectory of the first particle for $\textsf{t}\ge \textsf{t}_*$ by an independent Poisson random walk in continuous time $\textsf{t}$ of slope $\hat{\tau }_*+1$. In particular, $y_1'$ is started from $y_1'(\textsf{t}_*)$, and the new walk continues until it reaches the old trajectory of $y_1$ at some time $\textsf{t}_\circ \in [0,+\infty ]$. Note that the case $\textsf{t}_{\circ }= \infty$ happens with positive probability. Then, if $\textsf{t}_\circ =\infty$, we independently resample the whole trajectory to the right of $\textsf{t}_*$ by a trajectory with the new slope (jump rate) $\hat{\tau }_*+1$. We may think that each jump of the trajectory is an instantaneous “avalanch” whose slope $\hat{\tau }_*+1$ grows with time. This resembles the avalanch processes in, e.g., [PPH03, LD22], but we will not explore this connection further in the present paper. See Fig. 24 for an illustration of the process.

Proposition 10.10 for $q=0$ immediately implies the following coupling of Poisson processes with different slopes:

#### Proposition 10.13

Take a Poisson simple random walk of slope 1. Apply the dynamics on single-particle trajectories described above, evolving in continuous time $\hat{\tau }$. Then, the distribution of the resulting trajectory at each time $\hat{\tau }$ is the Poisson simple random walk of slope $\hat{\tau }+1$.

We obtain a Markov chain which preserves the Poisson process on [0, 1] as a corollary. The construction of this Markov process is based on the fact that, after one jump in the process $\tilde{\Xi }^{\rightarrow }$, we can dilate the horizontal line to decrease the slope of the Poisson simple random walk back to slope 1.

#### Definition 10.14

*(Markov chain preserving the Poisson random walk on [0, 1]).* Let $y_1(\textsf{t})$ be a trajectory of the Poisson random walk of slope 1 on [0, 1] with $y_1(0)=0$. Independently, sample two random variables: $\textsf{t}_*\in [0,1]$ with uniform distribution, and $\zeta >0$ with exponential distribution with parameter 1.

1. If $\textsf{t}_*>1 / (1+\zeta )$, do nothing and denote $Y_1(\textsf{t})=y_1(\textsf{t})$. This event happens with probability $1-e \Gamma (0,1)\approx 0.4$, where $\Gamma (0,1)=\int _1^\infty e^{-t}t^{-1}dt$ is the incomplete Gamma function.
2. If $\textsf{t}_*\le 1 / (1+\zeta )$, start a new independent Poisson random walk $y_1'(\textsf{t})$ of slope $\zeta +1$ from the location $(\textsf{t}_*,y_1(\textsf{t}_*)+1)$ and running in forward time $\textsf{t}\in [\textsf{t}_*, 1 / (1+\zeta )]$. When the new walk reaches the old trajectory of $y_1(\textsf{t})$ or $\textsf{t}$ reaches the coordinate $1 / (1+\zeta )$, stop the update. Denote the resulting trajectory by $Y_1(\textsf{t})$, $0\le \textsf{t}\le 1$.

Forget the configuration of $Y_1(\textsf{t})$ for $\textsf{t}\in (1 / (1+\zeta ), 1]$ after the update in both cases. Then, dilate the segment $[0,1 / (1+\zeta )]$ by means of multiplication by $1+\zeta$. In particular, the result of the application of the Markov transition operator is, by definition, the trajectory $Z_1(\textsf{t})=Y_1(\textsf{t} / (1+\zeta ))$, $0\le \textsf{t}\le 1$.

#### Corollary 10.15

Let $\{y_1(\textsf{t})\}_{0\le \textsf{t}\le 1}$, be a trajectory of the Poisson simple random walk on [0, 1] with $y_1(0)=0$. Then, the trajectory $\{Z_1(\textsf{t})\}_{0\le \textsf{t}\le 1}$ described in Definition 10.14 has the same distribution as $\{y_1(\textsf{t})\}_{0\le \textsf{t}\le 1}$.

To the best of our knowledge, Proposition 10.13 and Corollary 10.15 are new. It is possible to isolate the proof of Proposition 10.13 from the rest of the paper. That proof would essentially follow by iterating the Yang-Baxter equation and taking Poisson-type limits. It would be interesting to find direct proofs of Proposition 10.13 and Corollary 10.15 which would not rely on discrete models and Poisson limits.

## Data Availability

Data sharing not applicable to this article as no datasets were generated or analyzed during the current study.

## Footnotes

1 Note that we shift the indices for a better correspondence between particle systems and vertex models.

2 That is, the random time $\xi$ after which the clock rings is distributed as $\textrm{Prob}(\xi >s)=$ $e^{-\lambda s}, s>0$, where $\lambda =\alpha _{n-1}(1-q^{x_n-x_{n+1}-1})$ is the rate.

3 These two cases do not exhaust the full range of parameters $(q,\mu ,\nu )$ for which the weights are nonnegative. See, e.g., [BP18a, Section 6.6.1]: for additional cases leading to nonnegative weights.

4 An intertwining relation for general $\nu$ is also readily obtained in a continuous time limit from the shift operator for the *q*-Hahn TASEP, but in the present paper we omit this statement, as well as its limit to the beta polymer as in [Pet21, Section 6].

5 The word *jump* refers to jumps under continuous time Markov processes on spaces of trajectories, throughout the current Sect. 10. When a particle in a given trajectory changes its position (which is a piecewise constant function of $\textsf{t}$), we refer to this as an *increment* of this particle’s coordinate.
